# Mechanistic Insight into the Ring-Opening Polymerization of *ε*-Caprolactone and *L*-Lactide Using Ketiminate-Ligated Aluminum Catalysts

**DOI:** 10.3390/polym11091530

**Published:** 2019-09-19

**Authors:** Ya-Fan Lin, Nai-Yuan Jheng

**Affiliations:** 1 Department of Fragrance and Cosmetic Science, Kaohsiung Medical University, Kaohsiung 80708, Taiwan; 2 Department of Medicinal and Applied Chemistry, Kaohsiung Medical University, Kaohsiung 80708, Taiwan

**Keywords:** DFT calculation, ring-opening polymerization, *L*-lactides, *ε*-caprolactone, aluminum complexes, ketiminate ligands

## Abstract

The reactivity and the reaction conditions of the ring-opening polymerization of *ε*-caprolactone (*ε*-CL) and *L*-lactide (LA) initiated by aluminum ketiminate complexes have been shown differently. Herein, we account for the observation by studying the mechanisms on the basis of density functional theory (DFT) calculations. The calculations show that the ring-opening polymerization of *ε*-CL and LA are rate-determined by the benzoxide insertion and the C–O bond cleavage step, respectively. Theoretical computations suggest that the reaction temperature of *L*–LA polymerization should be higher than that of *ε*-CL one, in agreement with the experimental data. To provide a reasonable interpretation of the experimental results and to give an insight into the catalyst design, the influence of the electronic, steric, and thermal effects on the polymerization behaviors will be also discussed in this study.

## 1. Introduction

Utilization of the polyester such as poly(*ε*-caprolactone) (PCL) and poly(*L*-lactide) (PLA) as eco-friendly materials has been paid more and more attention [1,2,3,4,5,6]. Therefore, development of efficient synthetic strategies becomes of great interest to chemists. Among these strategies, ring-opening polymerization (ROP) catalyzed by the less toxic and inexpensive aluminum complexes is one of the useful and well-controlled methods [7,8,9,10,11,12,13,14,15,16,17,18,19,20,21,22,23,24,25,26,27,28,29,30]. The ring-opening polymerization catalyzed by an organometallic complex usually proceeds through a coordination-insertion mechanism [31,32]. Recent works have revealed that coordination number [33,34,35], the size effect of the chelate rings [36], steric and electronic effects of the auxiliary ligands surrounding the aluminum complexes [22,25,34,37,38,39,40,41,42,43,44] as well as the co-initiators would alter the reactivity and the properties of the products. Besides the reaction conditions would be very dependent on the ring size and the bulkiness of the monomers [45,46]. For example, *L*-lactide has been found to be bulkier than caprolactone since the Al–O bond enthalpy of the former are weaker than the later [47]. Several kinds of Al complexes as the initiators for the ring opening polymerization have been designed, and different factors that influence the reactivity have been discussed separately. Nevertheless, systematically studying the structures-activity relationship of the catalysts remains ongoing.

**Figure 1:**
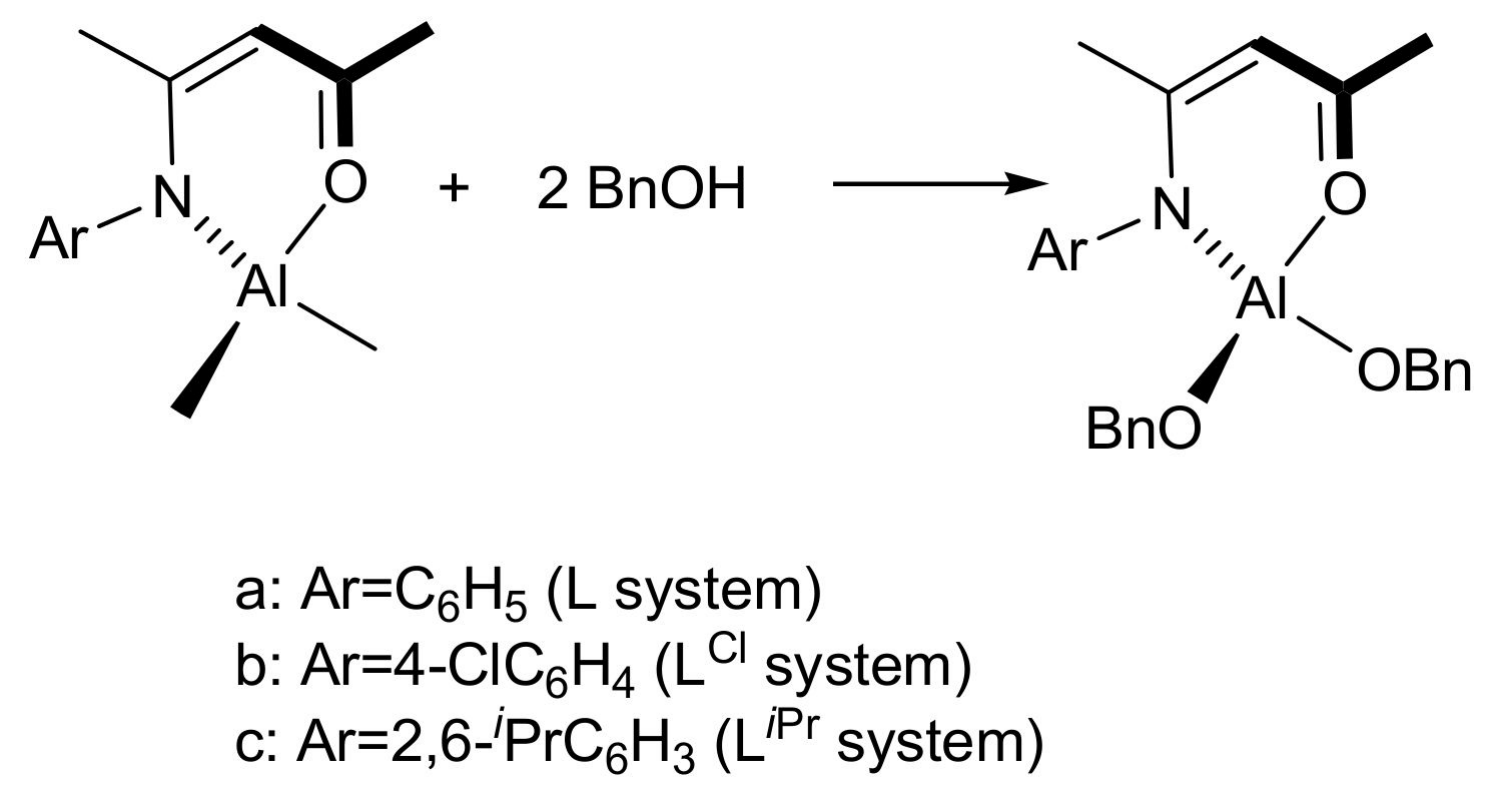
A series of aluminum ketiminate complexes bearing different substituents for the ring-opening polymerization of *ε*-caprolactone and *L*-lactide.

**Figure 2:**
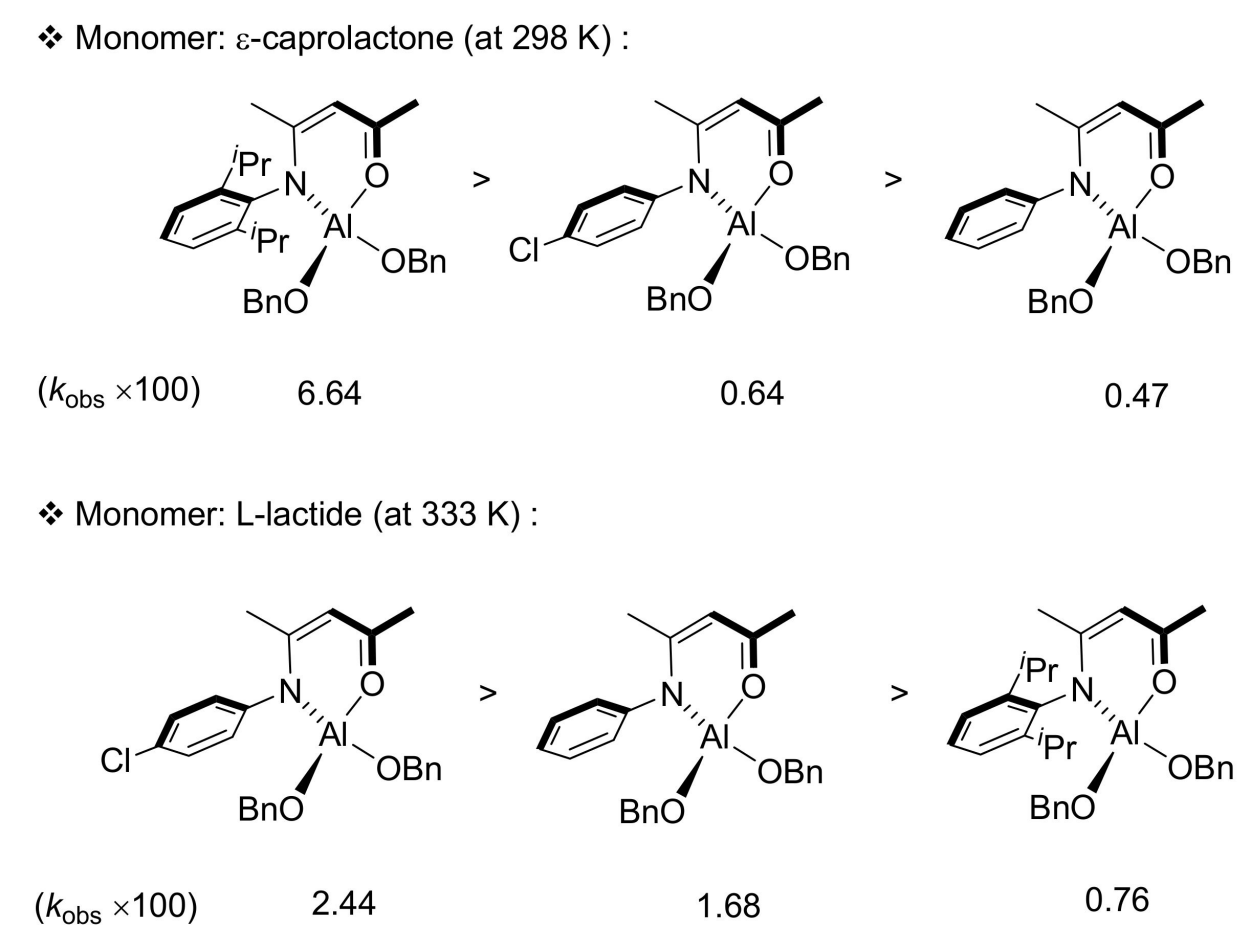
Experimental data summary for the ring-opening polymerization of *ε*-caprolactone and *L*-lactide initiated by aluminum ketiminate complexes.

In 2015, Chen et al. reported a series of aluminum complexes bearing ketiminate ligands to process the ring-opening polymerization of both *ε*-caprolactone (*ε*-CL) and *L*-lactide (*L*-LA) (Figure 1) [48,49]. By screening the pendant groups on the ketiminate skeleton of the aluminum complexes, the steric, electronic and chelating effects of the ring-opening polymerization of *ε*-caprolactone and *L*-lactide could be delineated by comparison of the experimental results. Previous experimental data has revealed that in the ketiminate-ligated Al systems, ring-opening polymerization of *ε*-caprolactone can proceed at room temperature. Nevertheless, if using *L*-lactide as a monomer, similar reactions cannot occur until raising up temperature to 333 K. Besides, the *k*_obs_ values of the ring-opening polymerization are influenced by the steric and electronic effects on the pendant N donors of kind of Al complex. It is worth noting that the orders of *k*_obs_ values in the polymerization of *ε*-caprolactone and *L*-LA have different tendencies. For examples, catalysts with the bulky isopropyl substituents facilitates the polymerization of *ε*-caprolactone, but formation of polylactide is not promoted by this kind Al complex (Figure 2). To get more insight into the mechanism, we decided to employ density functional theory (DFT) calculation to compare the two ring-opening polymerization systems catalyzed by the Al complex bearing a ketiminate ligand. Although only few cases of tetradentate Al catalysts have been evaluated by DFT calculations [50,51,52], it has been proven to be a useful tool to model the reaction intermediates and comprehend the reaction pathways of the tris- and penta-coordinate Al complexes [29,42,44,52,53,54,55,56,57,58,59,60,61,62]. Our aims in this theoretical study are: (a) to realize why polymerization of *ε*-caprolactone and *L*-lactide require different reaction temperatures; (b) to understand how the steric and electronic effects on the pendant group of the Al complexes would alter the catalytic abilities. To answer the above questions, we tried to compute the entire reaction pathways and estimate the activation barriers of the rate-determining steps. For the thermodynamic consideration, we also evaluated the bond enthalpy for the monomer coordination to the Al center. In addition, we chose L, L^Cl^, and L*^i^*^Pr^ (Figure 1) as pendant groups of the catalysts to evaluate the electronic and steric effects in both poly(*ε*-caprolactone) and poly(*L*-lactide) synthesis.

## 2. Materials and Methods 

All the energies and the geometries for the intermediates and transition states were evaluated using the Gaussian 16 package (Gaussian Inc., Pittsburgh, PA, USA) [63]. The Becke 3-parameter Lee-Yang Parr (B3LYP) hybrid functional combined with the D3 empirical dispersion correction developed by Grimme and the 6–31g(d) basis set was chosen as the calculation level [64,65,66,67,68,69,70,71]. The polarizable continuum model (PCM) was employed to describe the solvent effect of toluene [72]. To identify all the stationary-point structures, all reaction species were subjected to perform the frequency computation. All the resting states show real values in all types of motional frequencies, while the species with only one imaginary frequency were confirmed as transition states. Finally, the intrinsic reaction coordinate (IRC) or Quasi-IRC calculations were employed to make sure the connection between intermediates and the transition state [73,74,75,76]. The optimized Cartesian coordinates for all structures are provided in the Supplementary Materials.

## 3. Results and Discussion

**Figure 3:**
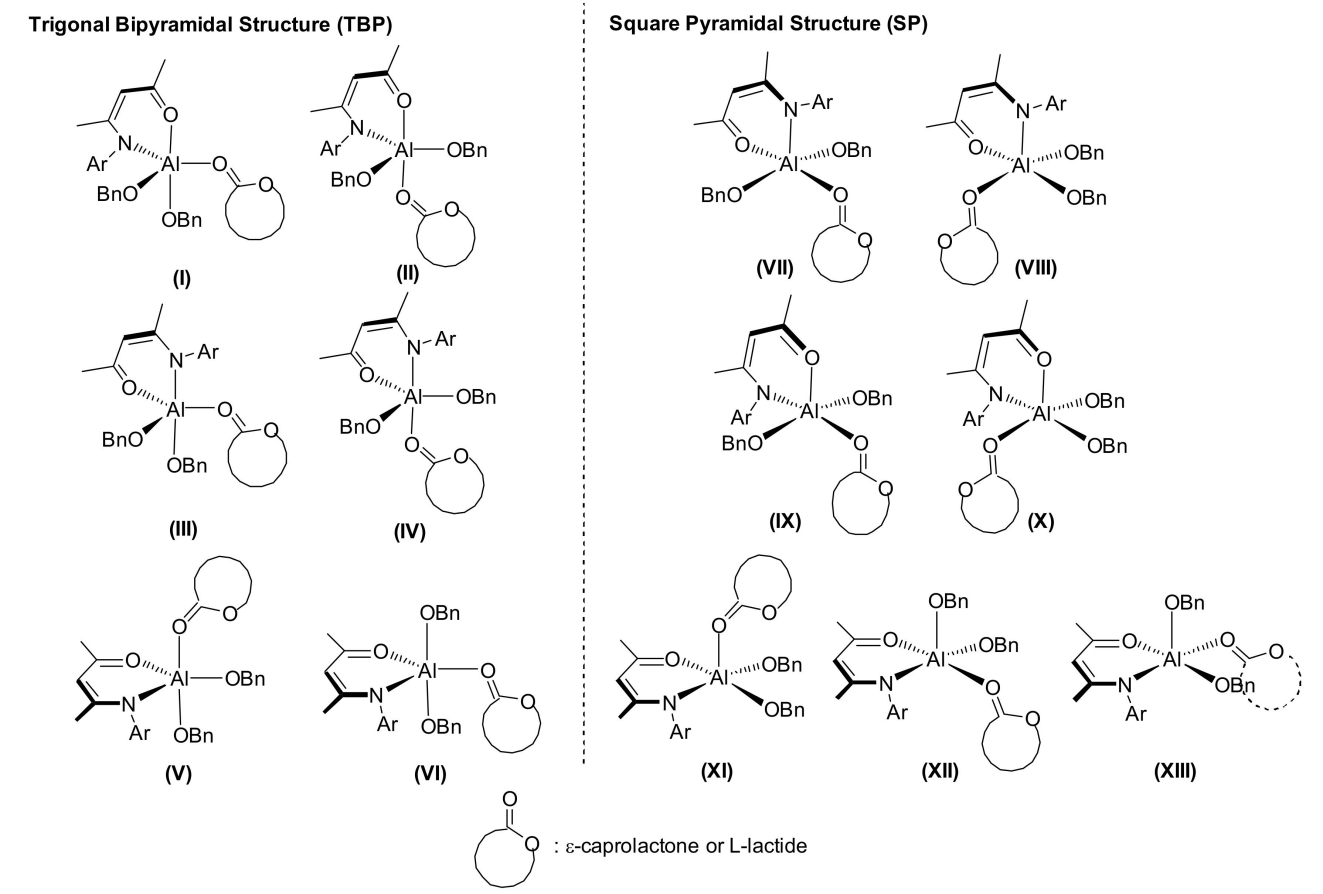
Thirteen possible initial geometries considered in this study.

**Scheme 1:**
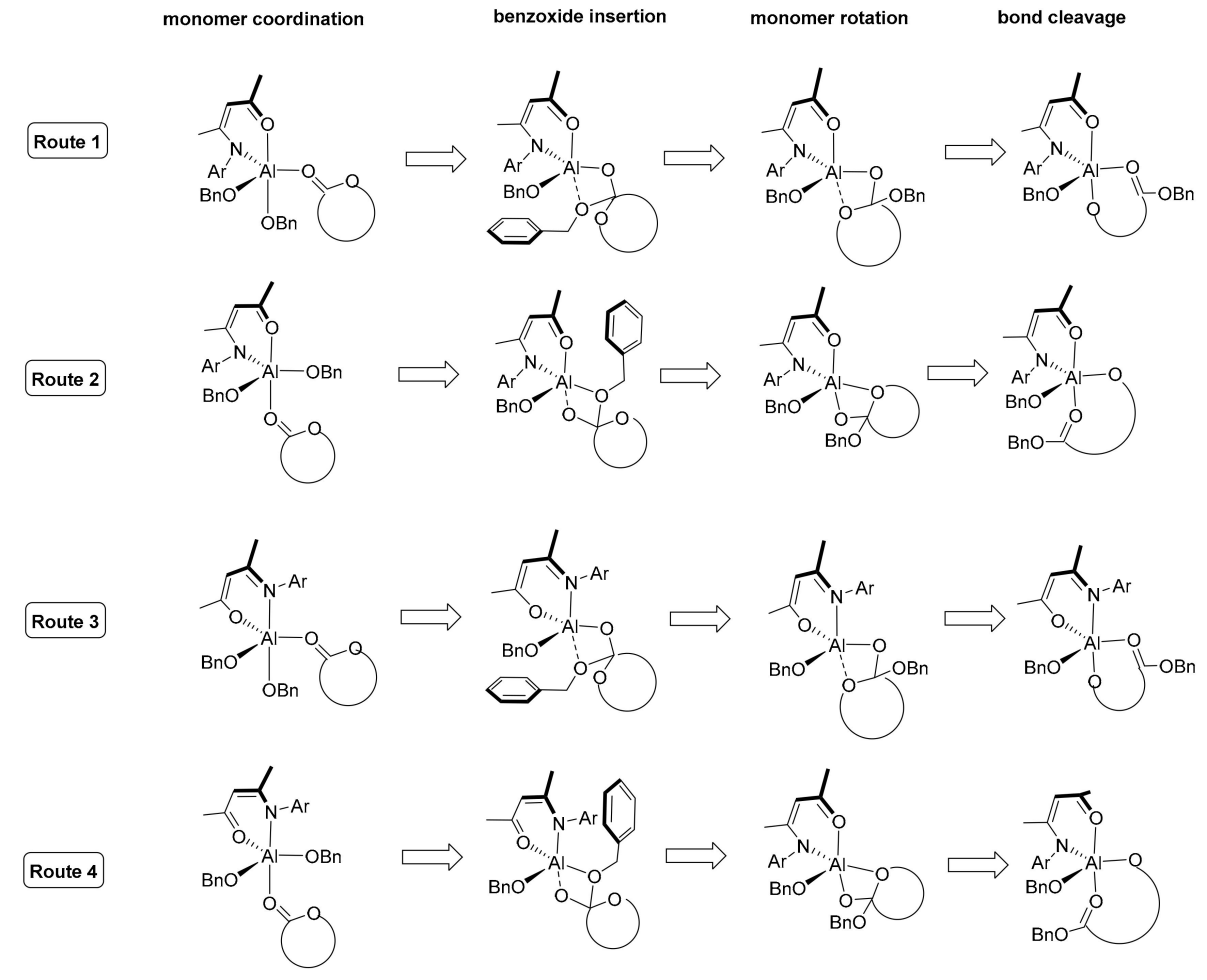
Four possible routes starting from four different triangular bipyramidal (TBP) monomer-coordinated aluminum isomers and follow the coordination-insertion mechanism.

Ring-opening polymerization of *ε*-caprolactone and *L*-lactide catalyzed by Al complexes would follow the coordination-insertion mechanism. The process starts from monomer coordination to the Al center, followed by benzoxide insertion to the C=O group of the monomer. Then, the resultant intermediate adjusts to the proper conformation to facilitate the breakage of C–O bond, completing the first ring-opening process. In the ketiminate-ligated Al systems, the monomer is approaching the Al center to give a penta-coordinate species. Considering 13 possible isomers can be generated, all of them were submitted as initial geometries and optimized by DFT calculation. (Figure 3). Calculation results show that only isomer I, II, III, and IV can be obtained as the optimal geometries, so mechanisms starting from isomer V–XIII are not considered. In order to obtain a more comprehensive view of the ROP mechanism, we discussed the four possible pathways from four triangular bipyramidal (TBP) isomer I–IV as the starting species (Scheme 1). By scanning the reaction coordinates, we confirmed that for the species N donor resides in the axial site (isomer III and IV), the monomer tends to be driven away from the aluminum center when benzoxide is approaching. As a result, Route 3 and 4 in Scheme 1 can be ruled out. The mechanistic calculations were concentrated on the pathways starting from I and II, which are initiated from the coordination isomers with the axial O donor (Route 1 and 2: the starting species is the isomer that monomer respectively coordinates on the equatorial and axial site).

### 3.1. Ring-Opening Polymerization of ε-Caprolactone

**Figure 4:**
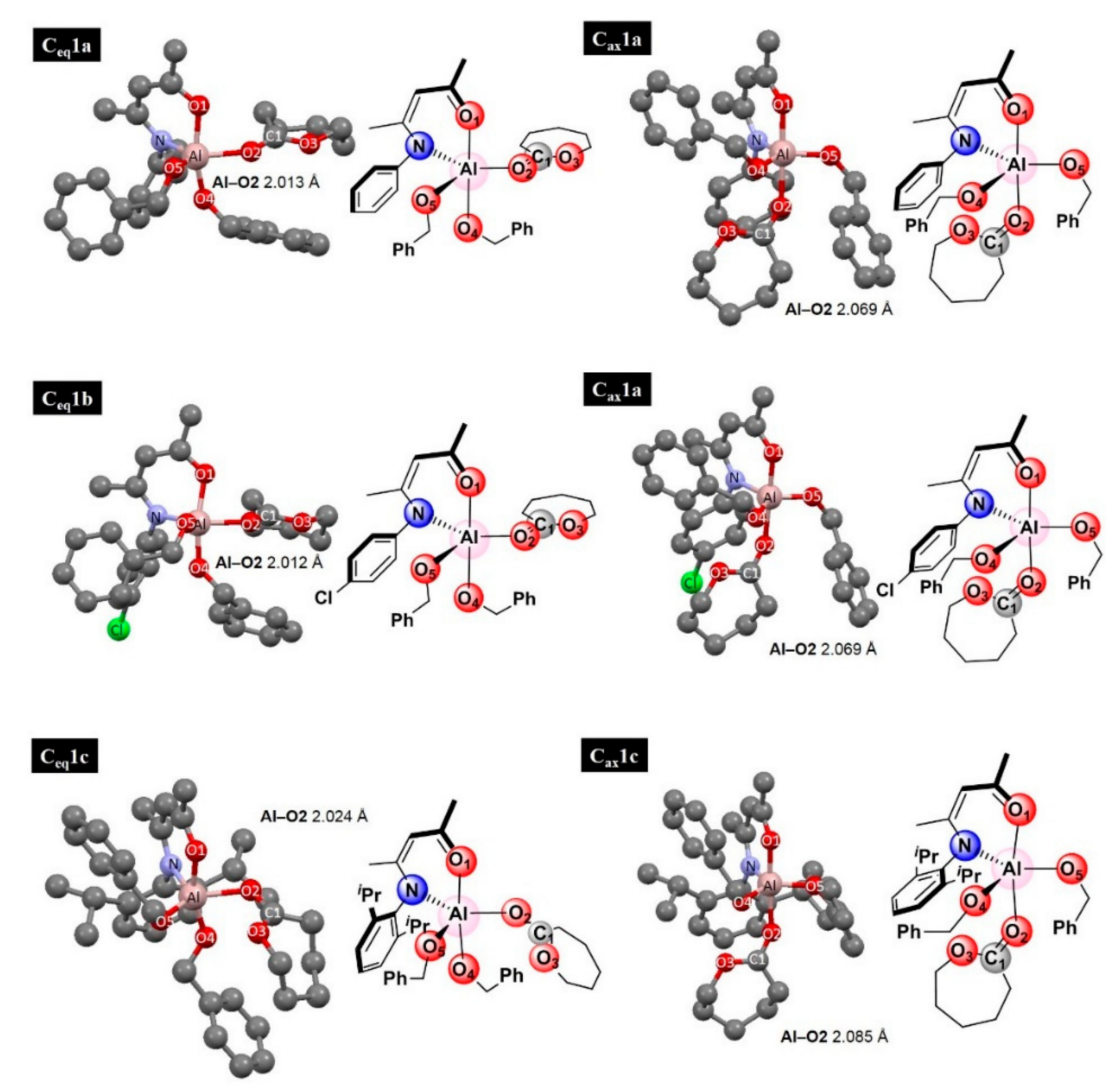
*ε*-Caprolactone coordinated aluminum complexes which bear L, L^Cl^, and L^iPr^ ligand systems and arrange in a TBP fashion.

Computing results showed that *ε*-caprolactone can occupy either equatorial or axial sites to give coordinated species in all three systems with different pendant groups. The *ε*-caprolactone equatorially and axially coordinated isomers are respectively denoted as C_eq_1a–c and C_ax_1a-c (a: L, b: L^Cl^, c: L^ipr^; Figure 4). The Al–O2 (Al–O_CL_) bond lengths in C_eq_1a, C_eq_1b, and C_eq_1c are respectively 2.013, 2.012, and 2.024 Å, shorter than those in C_ax_1a–c (C_ax_1a and C_ax_1b: 2.069; C_ax_1c: 2.085 Å). However, the bond enthalpies of Al-caprolactone in C_ax_1a–c (C_ax_1a: −12.7; C_ax_1b: −13.4; C_ax_1c: −9.1 kcal/mol) are slightly larger than those in C_eq_1a–c (C_eq_1a: −9.4; C_eq_1b: −10.4; C_eq_1c: −7.5 kcal/mol), showing coordination of *ε*-caprolactone at the axial site is energetically favorable. Both the equatorial and axial caprolactone coordination orientation are affected by the steric effect. Bulky substituents of isopropyl groups would hinder the monomer coordination. As a result, the bond enthalpies in the L*^i^*^Pr^ systems are the smallest among the three systems. On the other hand, the fact the Al–O2 bond enthalpies in the L^Cl^ systems are largest can be attributed to the chloro substituent, whose electron withdrawing property would make the Al center more positive and favorably binding to the *ε*-caprolactone.

**Figure 5:**
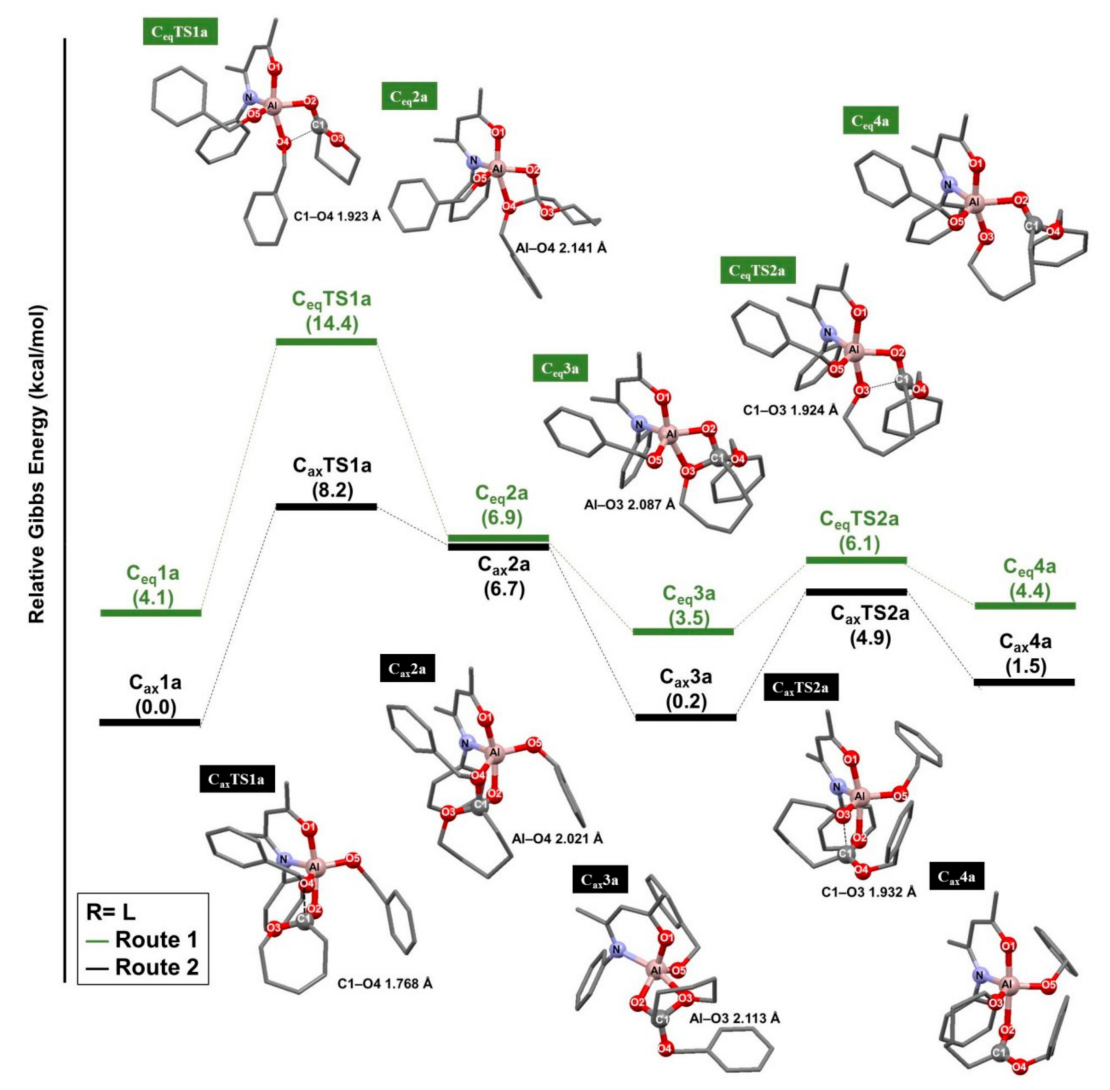
Reaction profile and optimized structures of the L system. (Monomer: *ε*-caprolactone).

**Figure 6:**
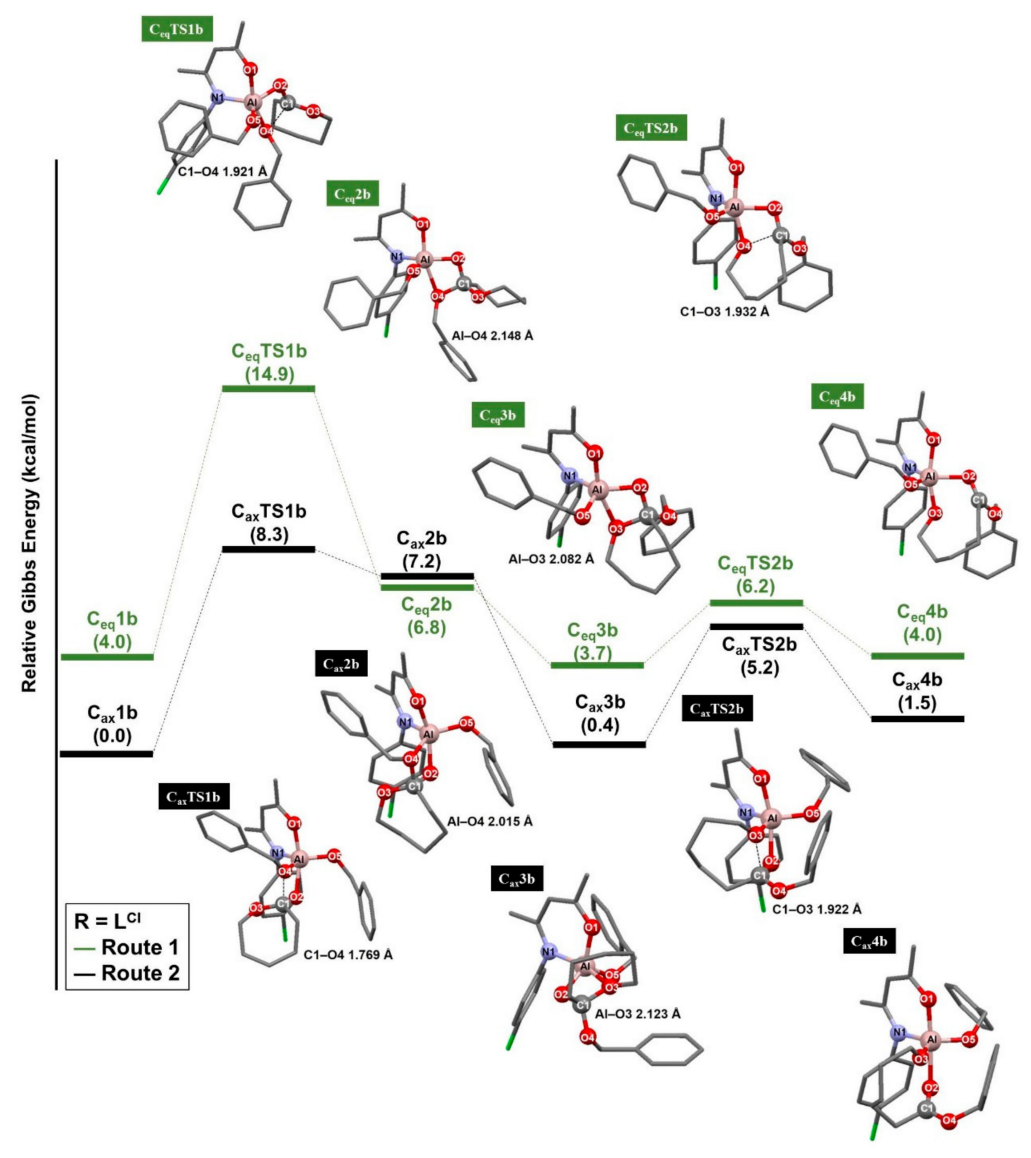
Reaction profile and optimized structures of the L^Cl^ system. (Monomer: *ε*-caprolactone).

**Figure 7:**
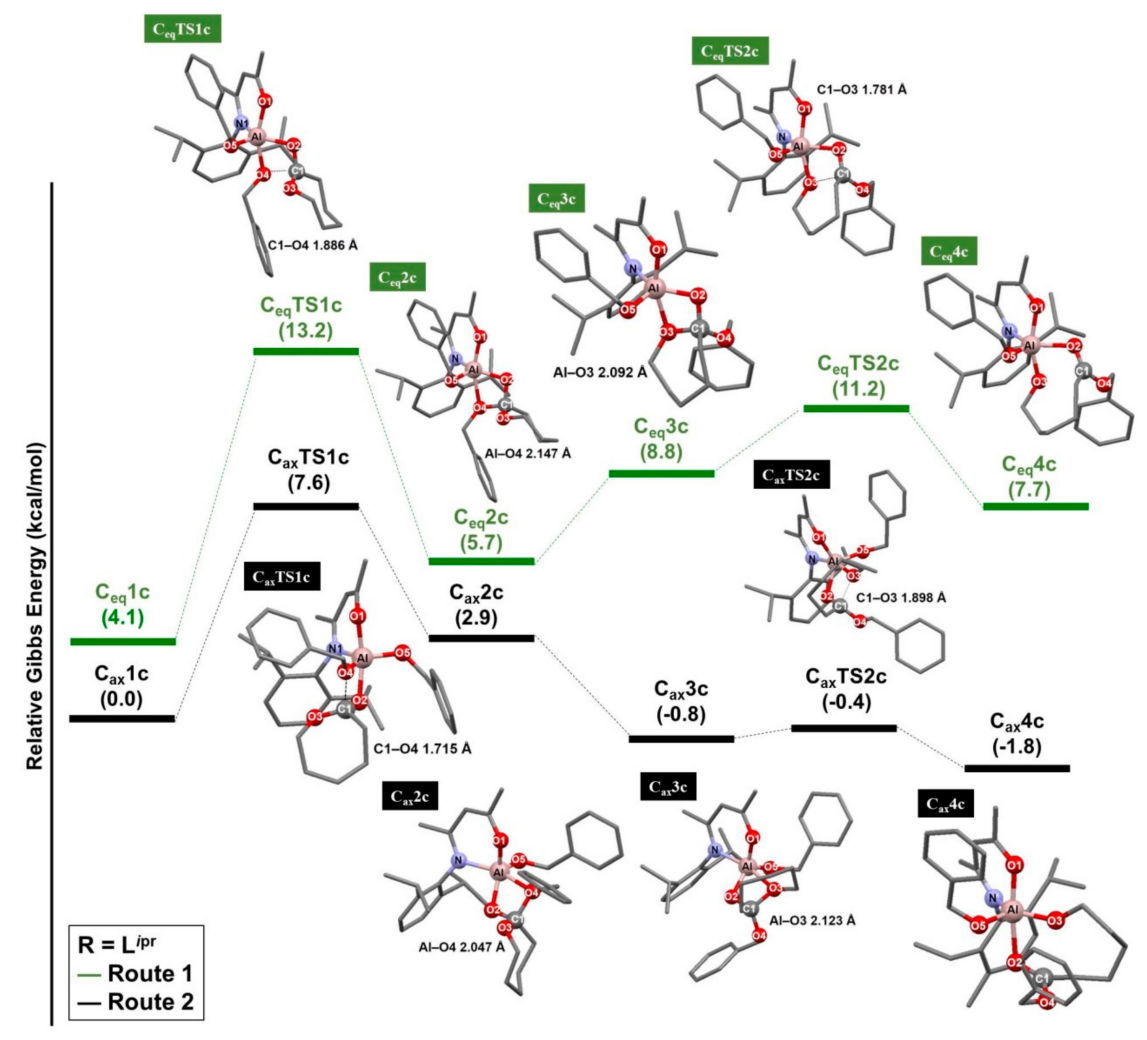
Reaction profile and optimized structures of the L*^i^*^Pr^ system. (Monomer: *ε*-caprolactone).

The Gibbs free energy profiles are shown in Figure 5, Figure 6 and Figure 7. The calculations reveal that the benzoxide insertion is the rate determining step of the *ε*-caprolactone ring-opening polymerization. The relative Gibbs energies in Route 1 are computed as 14.4 kcal/mol for C_eq_TS1a, 14.9 for C_eq_TS1b, and 13.2 for C_eq_TS1c (The differences in barrier height between C_eq_1a–c and C_eq_TS1a–c are 10.3, 10.9, and 9.1 kcal/mol). On the other hand, the activation Gibbs energy barriers of C_ax_TS1a, C_ax_TS1b and C_ax_TS1c are 8.2, 8.3, and 7.6 kcal/mol, respectively. The distances of C1–O4 in C_eq_TS1a, C_eq_TS1b and C_eq_TS1c are 1.923, 1.921, and 1.886 Å, while they are 1.768, 1.769, and 1.715 Å in C_ax_TS1a, C_ax_TS1b and C_ax_TS1c. The shorter distances of C1–O4 in C_eq_TS1c and C_ax_TS1c are probably due to the steric congestion imposed by the isopropyl groups. The benzoxide insertion steps which give the products of C_eq_2a–c (Route 1) and C_ax_2a–c (Route 2) were evaluated as endergonic reactions. Noteworthily, the L*^i^*^Pr^ systems of both routes require least input of energies to proceed the insertion step, probably because of the release of crowded space with the benzoxide group approaching to the *ε*-caprolactone [77]. The distances between Al and O4 of the inserted benzoxide in C_eq_2a–c are similar (2.141, 2.148, and 2.147 Å for C_eq_2a, C_eq_2b and C_eq_2c), while the aforementioned distance in C_ax_2c (2.047 Å) is longer than those in C_ax_2a (2.021 Å) and C_ax_2b (2.015 Å). After the benzoxide insertion, the Al-O2 bond in C_eq_2a–c/C_ax_2a–c rotates to yield C_eq_3a–c/C_ax_3a–c, which hold the structures in proper orientations to facilitate the C1-O3 bond breaking. Although C_eq_2a–c/C_eq_3a–c and C_ax_2a–c/C_ax_3a–c can be viewed as two pairs of linkage isomers, the computing result showed that the free energy differences between C_eq_3a–c/C_ax_3a–c and C_eq_2a–c/C_ax_2a–c are very dependent on their configurations and substituents. For the less bulky L and L^Cl^ ligand systems, the free energies of C_eq_3a–b and C_ax_3a–b are more favored than those of C_eq_2a–b and C_ax_2a–b with around 3 and 6.5 kcal/mol, respectively. For the L*^i^*^Pr^ ligand systems, 3.1 kcal/mol is required to drive C_eq_2c to C_eq_3c, while the pathway from C_ax_2c to C_ax_3c is exergonic by 3.7 kcal/mol. Al and O3 are separated with the distance of 2.087, 2.082, and 2.092 Å in C_eq_3a, C_eq_3b, and C_eq_3c and 2.113, 2.123, and 2.123 Å in C_ax_3a, C_ax_3b, and C_ax_3c. The distances reveal the Al…O3 interaction in C_eq_3a–c/C_ax_3a–c, driving the C1–O3 bond cleavage to complete the ring opening. C_eq_TS2a–c and C_ax_TS2a–c represent the possible transition-state structures for the ring-opening steps. The C1–O3 are elongated to 1.924, 1.932, and 1.898 Å in C_eq_TS2a, C_eq_TS2b, and C_eq_TS2c. The corresponding distances in C_ax_TS2a, C_ax_TS2b, and C_ax_TS2c are 1.932, 1.922, and 1.781 Å. The reactions then generate the final products C_eq_4a–c and C_ax_4a–c by conquering small energy barriers of ~2 (Route 1) and ~0.5–5 (Route 2) kcal/mol, respectively. In C_eq_4a–c and C_ax_4a–c, the C1–O3 of *ε*-caprolactone has been broken and reformed to a nine-membered ring with Al. Consequently, the coordination–insertion ring-opening reactions are slightly endergonic by less than 2 kcal/mol in both routes of L and L^Cl^ system. For the cases of L*^i^*^Pr^ systems, Route 1 is predicted to be 3.6 kcal/mol endergonic, whereas Route 2 is 1.8 kcal/mol exergonic.

Comparing the reaction barriers, Route 2 requires much lower free energy than Route 1 to make the reaction take place, indicating that coordination of *ε*-caprolactone in the axial position is the better direction to undergo nucleophilic addition. The corresponding structures of the transition states in Route 2 display a C1…O4 distance about 1.75 Å, shorter than those in Route 1 (about 1.92 Å). The shorter distance of C1…O4 in Route 2 also supports the fact that the benzoxide fragment can attack the carbonyl group of *ε*-caprolactone more effectively. As a result, Route 2 is a preferential pathway for the ring-opening polymerization of *ε*-caprolactone. Moreover, the activation barriers in the L^ipr^ system is about 1 kcal/mol smaller than those in the L and L^Cl^ systems, in consistency with the experimental results. It can be attributed to the sterically encumbered groups obliging the benzoxide fragment to get closer to the *ε*-caprolactone thus speeding up the occurrence of the insertion step, which determines the rate of the ring-opening polymerization of *ε*-caprolactone.

### 3.2. Ring-Opening Polymerization of L-Lactide

**Figure 8:**
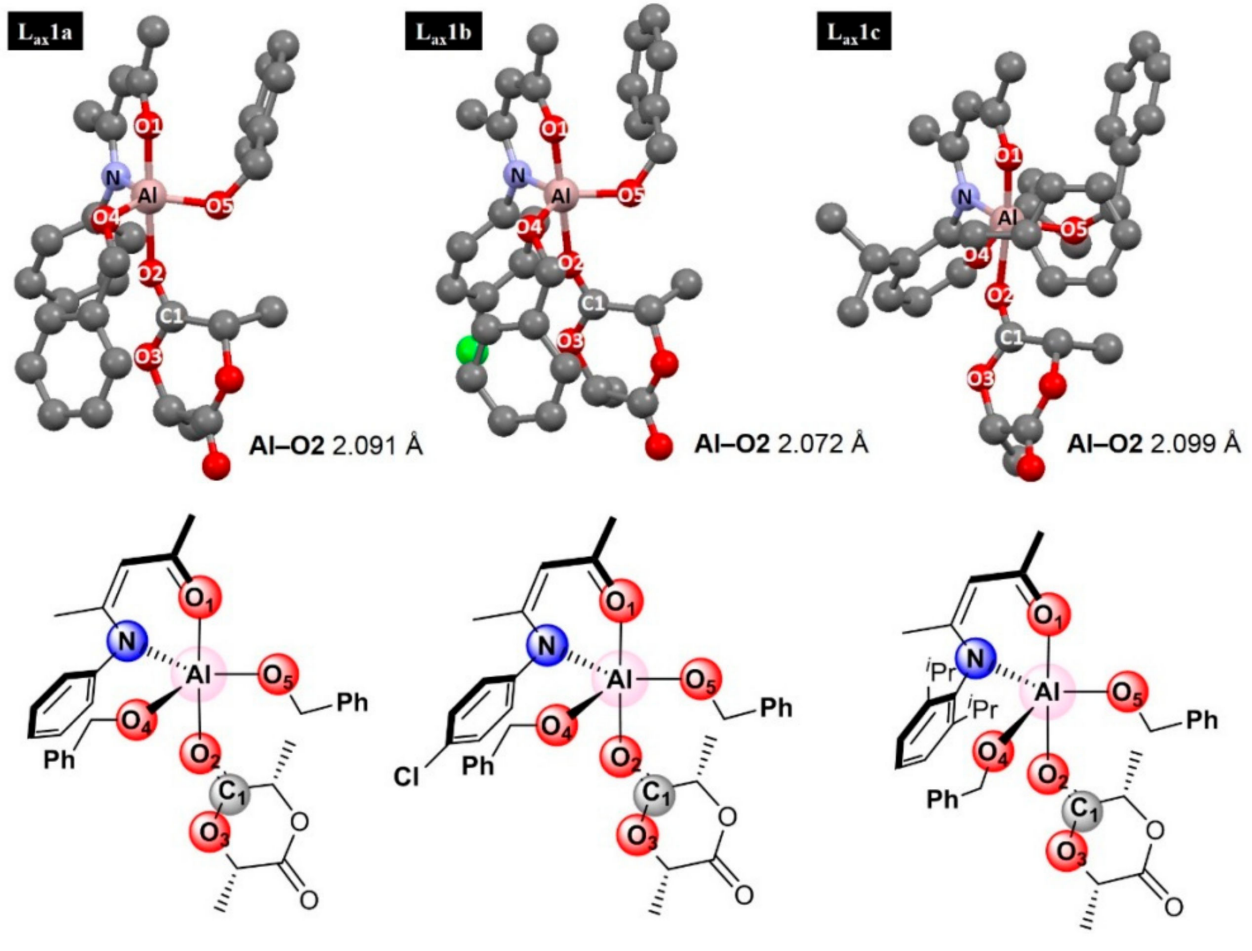
*L*-lactide coordinated aluminum complexes which bear L, L^Cl^, and L^iPr^ ligand systems and arrange in a TBP fashion.

The ring-opening polymerization mechanism of *L*-lactide shows different characteristics compared to *ε*-caprolactone polymerization. Although *L*-lactide can approach the Al center from the equatorial site, the results of the scan calculation show that benzoxide cannot attack the carbonyl group from this orientation. Hence, Route 2 is the only possible pathway for the ring-opening polymerization of *L*-lactide. The Al–O2 (Al–O_LA_) bond lengths of L_ax_1a, L_ax_1b, and L_ax_1c are 2.091, 2.072, and 2.099 Å, respectively (Figure 8). The values are slightly longer than those in C_ax_1a–c, probably because of the bulkier monomer of *L*-lactide. The bond enthalpies of Al–O2 in L_ax_1a–c are calculated to be larger than those in C_ax_1a−c (L_ax_1a: −12.6 kcal/mol; L_ax_1b: −13.9 kcal/mol; L_ax_1c: –12.3 kcal/mol). Among these species, the electron withdrawing inductive effect in L_ax_1b would lead to the more stable Al–O2 bond. Nevertheless, the calculation result in L_ax_1c implies that the steric hindrance does not exhibit obvious influence on the Al–O2 bond enthalpy.

**Figure 9:**
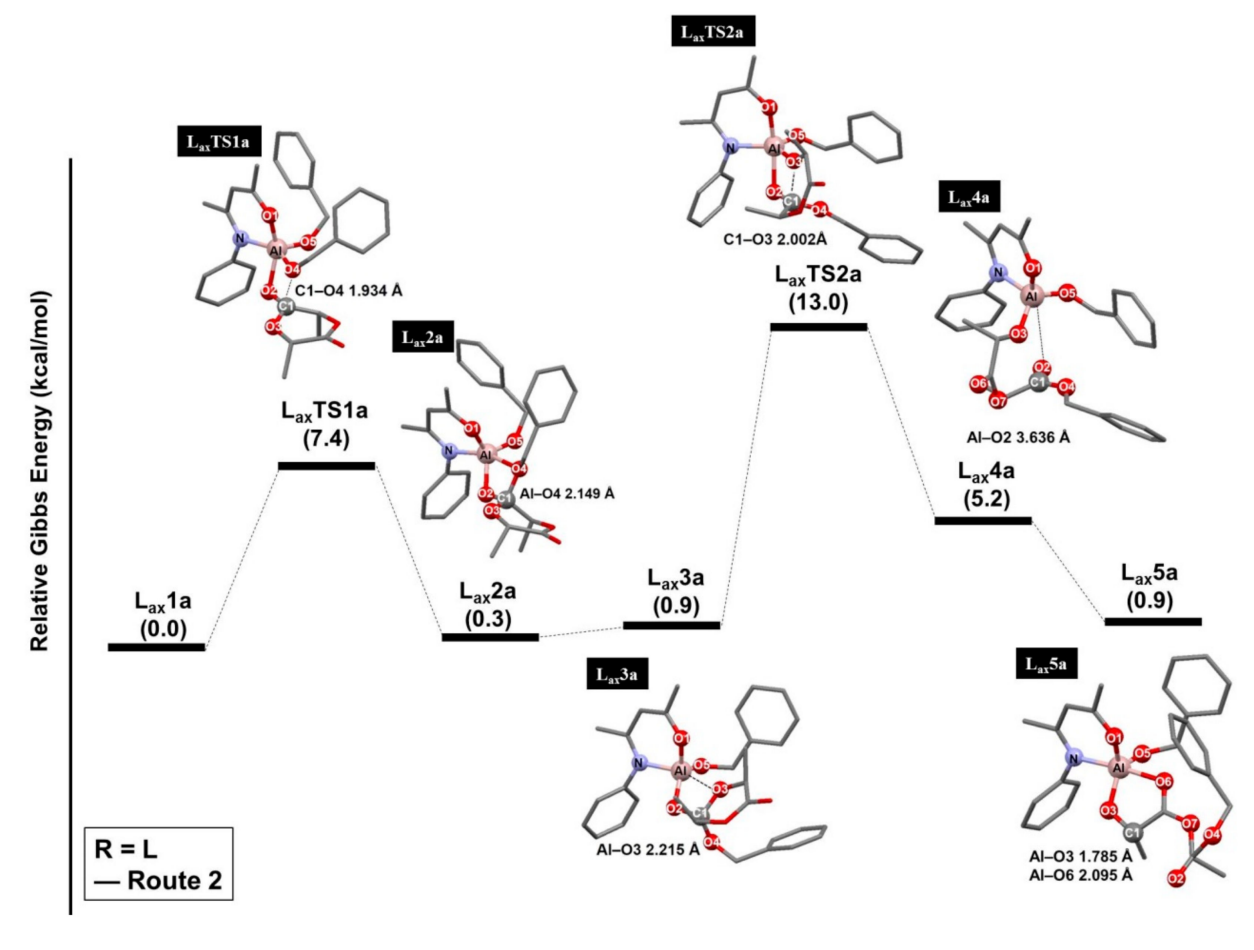
Reaction profile and optimized structures of the L system. (Monomer: *L*-lactide).

**Figure 10:**
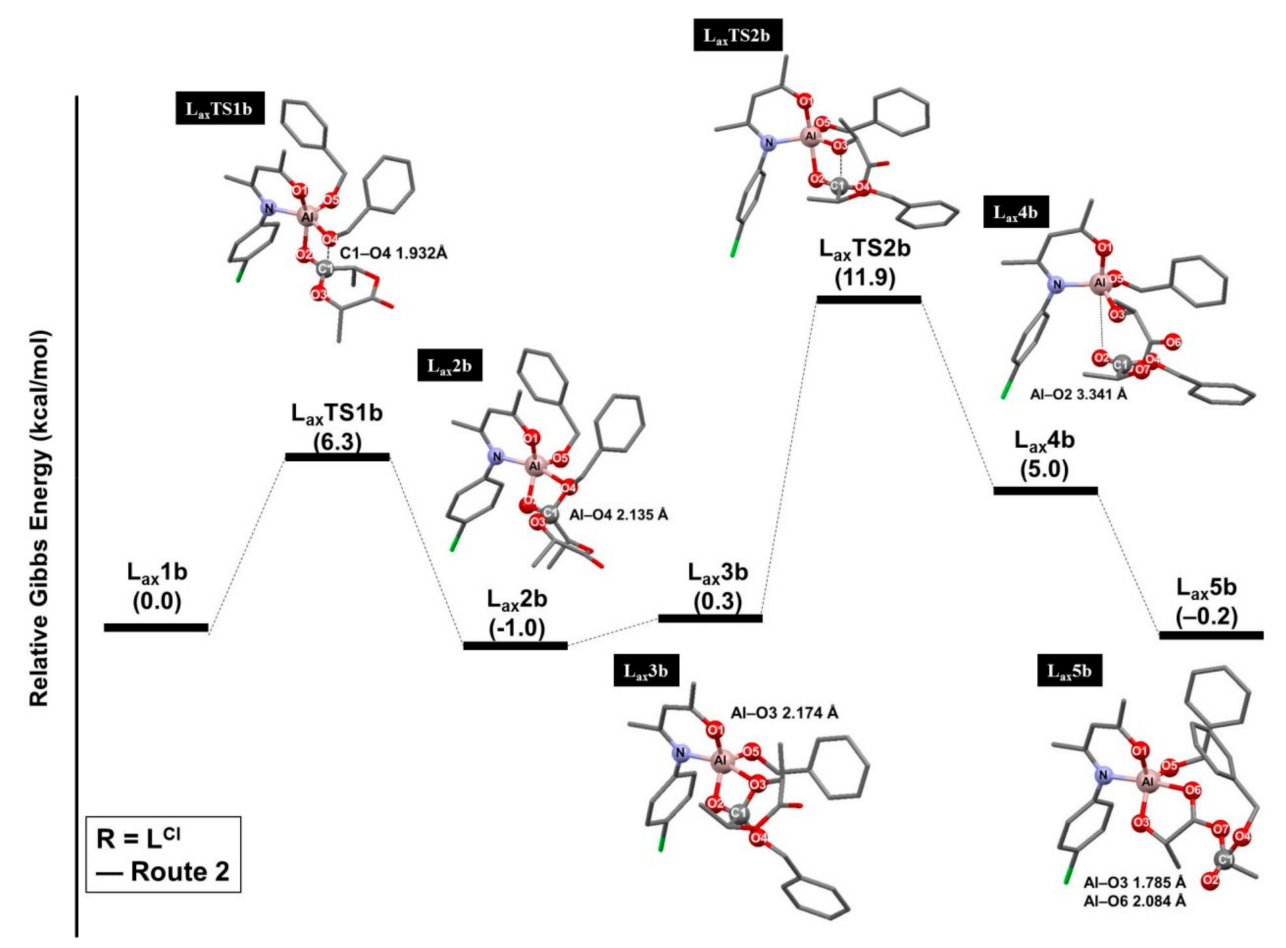
Reaction profile and optimized structures of the L^Cl^ system. (Monomer: *L*-lactide).

**Figure 11:**
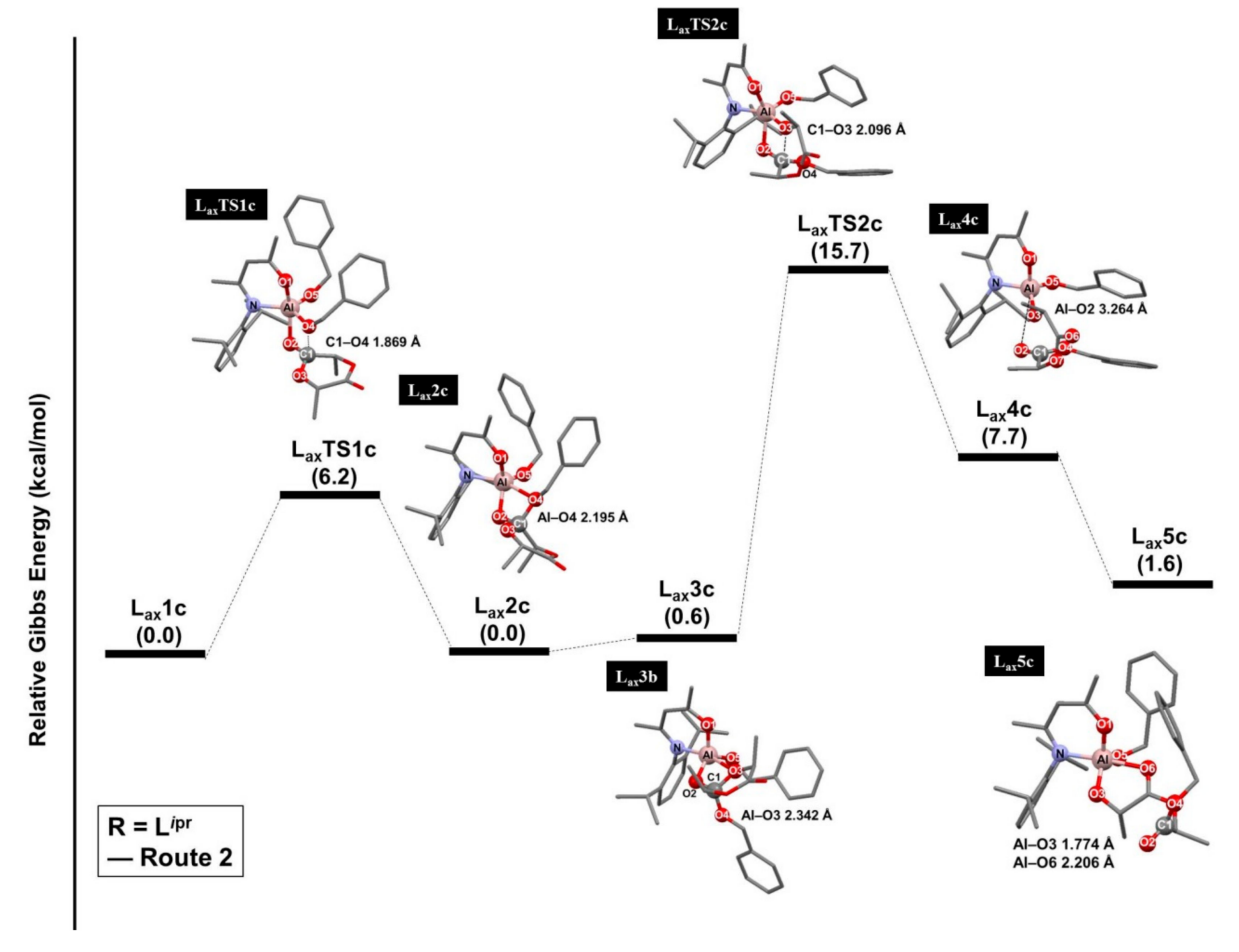
Reaction profile and optimized structures of the L*^i^*^Pr^ system. (Monomer: *L*-lactide).

DFT calculated Gibbs energy profiles of the proposed mechanism for the L_ax_1a-c catalyzed ring-opening polymerization of L-lactide are shown in Figure 9, Figure 10 and Figure 11. Different from the case of *ε*-caprolactone, the cleavage of the C–O bond is the rate-determining step in the ring-opening polymerization of L-lactide. The activation barriers generated from the nucleophilic addition of benzoxide to the carbonyl group on the L-lactide are about 7 kcal/mol, similar to the case of the ring-opening polymerization of *ε*-caprolactone. The corresponding transition states are determined to be a distorted square pyramidal structure, and the C1…O4 distances are separated by 1.934 Å in L_ax_TS1a, 1.932 Å in L_ax_TS1b, and 1.869 Å in L_ax_TS1c. The bulky pendant groups in L_ax_TS1c would lead to confined space and therefore result in the shorter C1…O4 distance. The intermediates produced from the insertion step are the species L_ax_2a–c with Al…O4 distances of 2.149, 2.135, and 2.195 Å in L_ax_2a, L_ax_2b, and L_ax_2c, respectively. L_ax_2a–c then undergo a bond rotation and yield L_ax_3a–c, the conformations more suitable for C–O cleavage. Compared to L_ax_2a–c, the Al…O3 distances in L_ax_3a–c are shortened while C1–O4 bonds are elongated. Noteworthily, the Al…O3 distance in L_ax_3c (2.342 Å) is much longer than in L_ax_3a (2.215 Å) and L_ax_3b (2.174 Å). The breakage of the C1–O3 bond involves the transition states L_ax_TS2a–c which lie 12.1, 11.6, and 15.1 kcal/mol above L_ax_3a, L_ax_3b, and L_ax_3c, respectively. The highest potential Gibbs energies of L_ax_TS2a–c reveal the C1–O3 breaking step determines the rate of the ring opening polymerization of L-lactide. The calculation results show that the C1–O3 distances are 2.002, 2.001, and 2.069 Å in L_ax_TS2a, L_ax_TS2b, and L_ax_TS2c, respectively. Following the intrinsic reaction coordinate, L_ax_TS2a–c give rise to the unstable intermediates L_ax_4a–c which are better described as a distorted tetrahedral species. Finally, L_ax_4a–c rearrange to the conformations of L_ax_5a–c, which adopt a TBP structure with the bidentate coordination of the ring-open *L*-lactide moiety.

The theoretical prediction shows that the L*^i^*^Pr^ system has the largest activation barrier among the three catalysts. Therefore, it would initiate the ring-opening polymerization of L-lactide more slowly, in agreement with the experimental results. The reaction is rate-determined by the ring opening step, the produced fragment of which would occupy more space. As a consequence, the reaction will be retarded by the bulky substituents. On the other hand, the reaction barriers of the ring-opening polymerization of *L*-lactide are estimated to be about twice larger than those of *ε*-caprolactone. The computational results have elucidated the fact that the ring-opening polymerization of *L*-lactide need to proceed at higher temperature in comparison to the case of *ε*-caprolactone.

## 4. Conclusions

In this study, we have shown that the ring-opening polymerization of *ε*-caprolactone is rate-determined by the step of benzoxide insertion, while the bond cleavage step is the slowest step in the case of ring-opening polymerization of L-lactide. Besides, theoretical analysis revealed that the reaction barriers of the ring-opening polymerization of L-lactide are higher than those of *ε*-caprolactone, corresponding to the experimental results. Finally, the pendant groups on the ketiminate ligand of the aluminum complexes play an essential role in catalyzing the ring-opening polymerization. In the *ε*-caprolactone ring-opening polymerization system, the crucial transition state is the stage benzoxide attacking the caprolactone. When the ketimate ligand bears more bulky groups, the congestion can be more largely released. Consequently, the reaction barrier is lowered to facilitate the occurrence of insertion when the pendant groups are more bulky. On the contrary, when L-lactides are loaded as monomers, the aluminum complexes supported by bulky groups are not conducive to the ring-opening polymerization. Since the rate-determining step of bond cleavage would lead to the occupation of space, the bulky substituents would go against the reaction to prevent from losing space. The calculation results are in agreement with the experimental data.

## Supplementary Materials

The optimized structures involved in this study are available online at https://www.mdpi.com/2073-4360/11/9/1530/s1, Table S1: Cartesian coordinate of Ceq1a, Table S2: Cartesian coordinate of CeqTS1a, Table S3: Cartesian coordinate of Ceq2a, Table S4: Cartesian coordinate of Ceq3a, Table S5: Cartesian coordinate of CeqTS2a, Table S6: Cartesian coordinate of Ceq4a, Table S7: Cartesian coordinate of Cax1a, Table S8: Cartesian coordinate of CaxTS1a, Table S9: Cartesian coordinate of Cax2a, Table S10: Cartesian coordinate of Cax3a, Table S11: Cartesian coordinate of CaxTS2a, Table S12: Cartesian coordinate of Cax4a, Table S13: Cartesian coordinate of Ceq1b, Table S14: Cartesian coordinate of CeqTS1b, Table S15: Cartesian coordinate of Ceq2b, Table S16: Cartesian coordinate of Ceq3b, Table S17: Cartesian coordinate of CeqTS2b, Table S18: Cartesian coordinate of Ceq5b, Table S19: Cartesian coordinate of Cax1b, Table S20: Cartesian coordinate of CaxTS1b, Table S21: Cartesian coordinate of Cax2b, Table S22: Cartesian coordinate of Cax3b, Table S23: Cartesian coordinate of CaxTS2b, Table S24: Cartesian coordinate of Cax4b, Table S25: Cartesian coordinate of Ceq1c, Table S26: Cartesian coordinate of CeqTS1c, Table S27: Cartesian coordinate of Ceq2c, Table S28: Cartesian coordinate of Ceq3c, Table S29: Cartesian coordinate of CeqTS2c, Table S30: Cartesian coordinate of Ceq4c, Table S31: Cartesian coordinate of Cax1c, Table S32: Cartesian coordinate of CaxTS1c, Table S33: Cartesian coordinate of Cax2c, Table S34: Cartesian coordinate of Cax3c, Table S35: Cartesian coordinate of CaxTS2c, Table S36: Cartesian coordinate of Cax4c, Table S37: Cartesian coordinate of Lax1a, Table S38: Cartesian coordinate of LaxTS1a, Table S39: Cartesian coordinate of Lax2a, Table S40: Cartesian coordinate of Lax3a, Table S41: Cartesian coordinate of LaxTS2a, Table 42: Cartesian coordinate of Lax4a, Table S43: Cartesian coordinate of Lax5a, Table S44: Cartesian coordinate of Lax1b, Table S45: Cartesian coordinate of LaxTS1b, Table S46: Cartesian coordinate of Lax2b, Table S47: Cartesian coordinate of Lax3b, Table S48: Cartesian coordinate of LaxTS2b, Table S49: Cartesian coordinate of Lax4b, Table S50: Cartesian coordinate of Lax5a, Table S51: Cartesian coordinate of Lax1c, Table S52: Cartesian coordinate of LaxTS1c, Table S53: Cartesian coordinate of Lax2c, Table S54: Cartesian coordinate of Lax3c, Table S55: Cartesian coordinate of LaxTS2c, Table S56: Cartesian coordinate of Lax4c, Table S57: Cartesian coordinate of Lax5c.
