# Supplementary material for: Mechanistic Insight into the Ring-Opening Polymerization of ε-Caprolactone and L-Lactide Using Ketiminate-Ligated Aluminum Catalysts
Source: Polymers (Basel). 2019 Sep 19;11(9):1530. doi: 10.3390/polym11091530 (PMC6780811; doi:10.3390/polym11091530)
Supplement: Supplementary file 1 [file polymers-11-01530-s001.pdf]

polymers

MDPI

# Mechanistic Insight into the Ring-Opening Polymerization of *ε*-Caprolactone and *L*-Lactide Using Ketiminate-Ligated Aluminum Catalysts

## Supplementary

## Theoretical Details

**Table S1.** Cartesian coordinate of **$C_{eq}$1a.**

| The number of atom coordinates (angstroms) | X | Y | Z |
|---|---|---|---|
| 13 | −0.323482 | 0.337649 | −0.65403 |
| 8 | 1.161713 | 0.223533 | −1.820921 |
| 8 | −1.394612 | 0.900602 | 0.640559 |
| 7 | 0.764338 | −0.677639 | 0.682025 |
| 6 | 2.544291 | −0.959982 | −3.332665 |
| 1 | 3.139089 | −0.058571 | −3.520829 |
| 1 | 3.188545 | −1.839713 | −3.407058 |
| 1 | 1.777173 | −1.007113 | −4.114377 |
| 6 | 1.870645 | −0.843508 | −1.990405 |
| 6 | 2.050853 | −1.808955 | −1.008855 |
| 1 | 2.677598 | −2.662678 | −1.234701 |
| 6 | 1.593687 | −1.655371 | 0.322813 |
| 6 | 2.088544 | −2.648696 | 1.351084 |
| 1 | 1.237863 | −3.115995 | 1.858173 |
| 1 | 2.69737 | −3.426135 | 0.884771 |
| 1 | 2.685224 | −2.150845 | 2.122173 |
| 6 | 0.52331 | −0.494529 | 2.08722 |
| 6 | −0.585004 | −1.077837 | 2.709352 |
| 1 | −1.274097 | −1.665196 | 2.11676 |
| 6 | −0.800923 | −0.874897 | 4.071296 |
| 1 | −1.667152 | −1.328451 | 4.54579 |
| 6 | 0.079138 | −0.087704 | 4.822273 |
| 6 | 1.182286 | 0.497525 | 4.198596 |
| 1 | 1.870375 | 1.115929 | 4.769144 |
| 6 | 1.403925 | 0.292588 | 2.83431 |
| 1 | 2.258185 | 0.744332 | 2.337666 |
| 6 | −2.38566 | 1.879894 | 0.625012 |
| 1 | −2.565771 | 2.298939 | −0.378829 |
| 1 | −3.344955 | 1.431372 | 0.944368 |
| 6 | −2.058807 | 3.022349 | 1.574813 |

| | | | |
|---|---|---|---|
| 6 | −1.421512 | 2.745183 | 2.792053 |
| 1 | −1.150942 | 1.719927 | 3.016539 |
| 6 | −1.114889 | 3.772575 | 3.68469 |
| 1 | −0.612791 | 3.537861 | 4.620389 |
| 6 | −1.446382 | 5.096175 | 3.376712 |
| 1 | −1.207597 | 5.898017 | 4.070915 |
| 6 | −2.087347 | 5.380447 | 2.168211 |
| 1 | −2.346411 | 6.406545 | 1.917295 |
| 6 | −2.384777 | 4.348491 | 1.271752 |
| 1 | −2.864665 | 4.57824 | 0.322459 |
| 8 | −1.340435 | −0.638347 | −1.716506 |
| 6 | −2.590878 | −1.138457 | −1.323274 |
| 1 | −3.103524 | −1.531776 | −2.218056 |
| 1 | −3.246221 | −0.352768 | −0.914088 |
| 6 | −2.495314 | −2.259852 | −0.297354 |
| 6 | −1.419918 | −3.156878 | −0.332254 |
| 1 | −0.650617 | −3.020489 | −1.085871 |
| 6 | −1.316852 | −4.18389 | 0.60712 |
| 1 | −0.471341 | −4.867022 | 0.569835 |
| 6 | −2.291876 | −4.332464 | 1.59825 |
| 1 | −2.208277 | −5.126833 | 2.335449 |
| 6 | −3.371253 | −3.4465 | 1.637802 |
| 1 | −4.130366 | −3.546297 | 2.409854 |
| 6 | −3.468026 | −2.417905 | 0.696512 |
| 1 | −4.298643 | −1.716649 | 0.74682 |
| 6 | 0.582842 | 3.161007 | −0.971917 |
| 6 | 1.853549 | 2.933022 | −0.190173 |
| 6 | 1.832515 | 3.592028 | 1.208036 |
| 6 | 2.109265 | 5.0995 | 1.192084 |
| 6 | 1.123717 | 5.92326 | 0.354015 |
| 6 | 1.07213 | 5.543124 | −1.121703 |
| 1 | 2.70262 | 3.311028 | −0.774614 |
| 1 | 0.875015 | 3.379261 | 1.694181 |
| 1 | 3.127575 | 5.271589 | 0.813148 |
| 1 | 0.111091 | 5.844353 | 0.764918 |
| 1 | 1.980539 | 1.853211 | −0.107102 |
| 1 | 2.60258 | 3.09833 | 1.811094 |
| 1 | 2.091747 | 5.471328 | 2.223341 |
| 1 | 1.408193 | 6.98217 | 0.404073 |
| 1 | 0.575744 | 6.317146 | −1.708328 |
| 1 | 2.069808 | 5.378269 | −1.544566 |
| 8 | −0.208464 | 2.256507 | −1.253584 |

| | | | |
|---|---|---|---|
| 8 | 0.245381 | 4.372305 | −1.382589 |
| 1 | −0.098273 | 0.071742 | 5.882404 |

**Table S2.** Cartesian coordinate of **$C_{eq}$TS1a.**

| The number of atom coordinates (angstroms) | X | Y | Z |
|---|---|---|---|
| 13 | −0.047683 | 0.376727 | −0.751961 |
| 8 | 0.445172 | −0.906732 | −2.035728 |
| 8 | −0.216154 | 2.063351 | 0.186357 |
| 7 | 0.727928 | −0.658712 | 0.730169 |
| 6 | 1.254225 | −1.892753 | −1.915219 |
| 6 | 1.570972 | −2.628715 | −3.192175 |
| 1 | 0.640147 | −3.009557 | −3.628131 |
| 1 | 1.999856 | −1.926861 | −3.916114 |
| 1 | 2.263395 | −3.460187 | −3.040023 |
| 6 | 1.791569 | −2.312066 | −0.698238 |
| 1 | 2.442271 | −3.177217 | −0.708958 |
| 6 | 1.478589 | −1.757604 | 0.559489 |
| 6 | 2.016539 | −2.491861 | 1.770439 |
| 1 | 1.190139 | −2.861125 | 2.387376 |
| 1 | 2.633041 | −3.340319 | 1.469167 |
| 1 | 2.612059 | −1.82958 | 2.406231 |
| 6 | 0.412554 | −0.296718 | 2.090045 |
| 6 | −0.759454 | −0.782523 | 2.679974 |
| 1 | −1.416046 | −1.422152 | 2.102056 |
| 6 | −1.067415 | −0.457802 | 4.002704 |
| 1 | −1.983257 | −0.839908 | 4.445232 |
| 6 | −0.205839 | 0.347657 | 4.750264 |
| 6 | 0.966276 | 0.83105 | 4.162977 |
| 1 | 1.642142 | 1.463312 | 4.732094 |
| 6 | 1.274066 | 0.511188 | 2.840261 |
| 1 | 2.188214 | 0.877894 | 2.388417 |
| 6 | −1.354467 | 2.903885 | 0.091511 |
| 1 | −1.169186 | 3.674024 | −0.670295 |
| 1 | −2.201338 | 2.319051 | −0.281514 |
| 6 | −1.727984 | 3.565283 | 1.401186 |
| 6 | −1.505291 | 2.919447 | 2.62101 |
| 1 | −1.022646 | 1.952161 | 2.619403 |
| 6 | −1.888413 | 3.517001 | 3.822374 |
| 1 | −1.700573 | 2.998019 | 4.758789 |
| 6 | −2.507394 | 4.769496 | 3.820335 |
| 1 | −2.804971 | 5.236498 | 4.755672 |
| 6 | −2.738366 | 5.420392 | 2.605707 |

| | | | |
|---|---|---|---|
| 1 | −3.215612 | 6.397125 | 2.592192 |
| 6 | −2.348279 | 4.821583 | 1.405833 |
| 1 | −2.525522 | 5.336217 | 0.462976 |
| 8 | −1.764941 | 0.116602 | −1.045842 |
| 6 | −2.811232 | −0.150066 | −0.166416 |
| 1 | −3.757813 | 0.208846 | −0.606631 |
| 1 | −2.704766 | 0.394377 | 0.790834 |
| 6 | −2.976342 | −1.62773 | 0.171781 |
| 6 | −2.192197 | −2.608676 | −0.441477 |
| 1 | −1.47771 | −2.306322 | −1.198107 |
| 6 | −2.324888 | −3.952321 | −0.079199 |
| 1 | −1.70123 | −4.70202 | −0.560518 |
| 6 | −3.248084 | −4.332868 | 0.895835 |
| 1 | −3.348337 | −5.377194 | 1.180185 |
| 6 | −4.044228 | −3.357981 | 1.506436 |
| 1 | −4.76713 | −3.642815 | 2.267116 |
| 6 | −3.906941 | −2.017669 | 1.145829 |
| 1 | −4.517778 | −1.260305 | 1.635038 |
| 6 | 1.292569 | 2.57349 | −0.892447 |
| 6 | 2.373415 | 2.709231 | 0.166624 |
| 6 | 2.096242 | 3.602184 | 1.382327 |
| 6 | 2.15022 | 5.106218 | 1.101328 |
| 6 | 1.150602 | 5.577309 | 0.040282 |
| 6 | 1.383629 | 4.986912 | −1.347489 |
| 1 | 3.277513 | 3.045885 | −0.363726 |
| 1 | 1.12278 | 3.327891 | 1.796848 |
| 1 | 3.165543 | 5.381419 | 0.778242 |
| 1 | 0.124143 | 5.370258 | 0.360164 |
| 1 | 2.573469 | 1.686149 | 0.488414 |
| 1 | 2.846749 | 3.362487 | 2.146416 |
| 1 | 1.961906 | 5.649931 | 2.035105 |
| 1 | 1.230515 | 6.667079 | −0.069345 |
| 1 | 0.849433 | 5.565934 | −2.103061 |
| 1 | 2.450699 | 4.998149 | −1.604158 |
| 8 | 1.239575 | 1.466801 | −1.56423 |
| 8 | 0.879397 | 3.645619 | −1.575891 |
| 1 | −0.445046 | 0.598609 | 5.779875 |

**Table S3.** Cartesian coordinate of **$C_{eq}$2a.**

| The number of atom coordinates (angstroms) | X | Y | Z |
|---|---|---|---|
| 13 | 0.428558 | 0.22589 | −0.787086 |
| 8 | 1.534854 | −0.637712 | −1.996662 |

| | | | |
|---|---|---|---|
| 8 | −0.28204 | 1.675766 | 0.618848 |
| 7 | 0.834452 | −1.035479 | 0.646414 |
| 6 | 1.779969 | −1.901217 | −2.007876 |
| 6 | 2.345568 | −2.436978 | −3.296375 |
| 1 | 1.63097 | −2.251933 | −4.106551 |
| 1 | 3.260687 | −1.887325 | −3.544583 |
| 1 | 2.568048 | −3.505558 | −3.247487 |
| 6 | 1.581985 | −2.735498 | −0.909464 |
| 1 | 1.837066 | −3.781923 | −1.019407 |
| 6 | 1.214093 | −2.291371 | 0.380681 |
| 6 | 1.269626 | −3.312414 | 1.495895 |
| 1 | 0.270491 | −3.461003 | 1.918912 |
| 1 | 1.643489 | −4.270371 | 1.130172 |
| 1 | 1.914158 | −2.968964 | 2.311029 |
| 6 | 0.629633 | −0.691283 | 2.029371 |
| 6 | −0.59547 | −0.941072 | 2.655752 |
| 1 | −1.408371 | −1.369278 | 2.082768 |
| 6 | −0.752296 | −0.661947 | 4.014498 |
| 1 | −1.705617 | −0.867871 | 4.493488 |
| 6 | 0.30443 | −0.12245 | 4.752199 |
| 6 | 1.517367 | 0.157202 | 4.116265 |
| 1 | 2.340847 | 0.590815 | 4.67714 |
| 6 | 1.680251 | −0.124293 | 2.758112 |
| 1 | 2.621352 | 0.078966 | 2.25546 |
| 6 | −1.626486 | 2.071938 | 0.966572 |
| 1 | −2.143198 | 2.420502 | 0.07118 |
| 1 | −2.081436 | 1.134903 | 1.299239 |
| 6 | −1.675501 | 3.113522 | 2.052635 |
| 6 | −1.011997 | 2.898143 | 3.265835 |
| 1 | −0.456998 | 1.979308 | 3.410039 |
| 6 | −1.039755 | 3.867439 | 4.266948 |
| 1 | −0.512597 | 3.692076 | 5.201303 |
| 6 | −1.740417 | 5.061339 | 4.069126 |
| 1 | −1.759983 | 5.818671 | 4.848356 |
| 6 | −2.41129 | 5.278824 | 2.863888 |
| 1 | −2.953041 | 6.206713 | 2.70056 |
| 6 | −2.37415 | 4.308993 | 1.858883 |
| 1 | −2.87376 | 4.489196 | 0.910293 |
| 8 | −1.066958 | −0.089725 | −1.618928 |
| 6 | −2.432409 | −0.229062 | −1.377338 |
| 1 | −2.923313 | −0.507691 | −2.324956 |

| | | | |
|---|---|---|---|
| 1 | −2.897007 | 0.719784 | −1.067703 |
| 6 | −2.763769 | −1.295731 | −0.343816 |
| 6 | −2.093368 | −2.52638 | −0.370011 |
| 1 | −1.322075 | −2.690197 | −1.116535 |
| 6 | −2.387265 | −3.517601 | 0.565903 |
| 1 | −1.855418 | −4.465367 | 0.533994 |
| 6 | −3.360475 | −3.294304 | 1.546573 |
| 1 | −3.585167 | −4.064004 | 2.280054 |
| 6 | −4.037603 | −2.073868 | 1.578335 |
| 1 | −4.79166 | −1.887586 | 2.338757 |
| 6 | −3.738593 | −1.082861 | 0.637366 |
| 1 | −4.264298 | −0.130689 | 0.672334 |
| 6 | 0.656607 | 2.617763 | −0.040286 |
| 6 | 1.60883 | 3.274785 | 0.988172 |
| 6 | 1.33247 | 4.724467 | 1.413008 |
| 6 | 1.652661 | 5.771124 | 0.331947 |
| 6 | 0.657895 | 5.809124 | −0.835585 |
| 6 | 0.490041 | 4.475204 | −1.557317 |
| 1 | 2.609141 | 3.220777 | 0.543545 |
| 1 | 0.297886 | 4.842046 | 1.744678 |
| 1 | 2.664458 | 5.585898 | −0.059572 |
| 1 | −0.329718 | 6.117624 | −0.467249 |
| 1 | 1.620936 | 2.616703 | 1.862014 |
| 1 | 1.961749 | 4.929884 | 2.289038 |
| 1 | 1.680183 | 6.765639 | 0.796792 |
| 1 | 0.977085 | 6.563099 | −1.568839 |
| 1 | −0.147367 | 4.594328 | −2.439396 |
| 1 | 1.450542 | 4.06719 | −1.894829 |
| 8 | 1.332839 | 1.772309 | −0.860929 |
| 8 | −0.178449 | 3.518094 | −0.72303 |
| 1 | 0.179655 | 0.092723 | 5.80973 |

**Table S4.** Cartesian coordinate of **$C_{eq}$3a.**

| The number of atom coordinates (angstroms) | X | Y | Z |
|---|---|---|---|
| 13 | −0.04825 | 0.527757 | −0.481118 |
| 8 | 0.912934 | −0.525644 | −1.66322 |
| 8 | 0.735531 | 3.983643 | 0.388979 |
| 7 | 0.417504 | −0.578612 | 1.056068 |
| 6 | 1.300557 | −1.745464 | −1.507686 |
| 6 | 1.823145 | −2.417462 | −2.748371 |
| 1 | 2.672763 | −1.846062 | −3.139386 |
| 1 | 2.132846 | −3.449117 | −2.567099 |

| | | | |
|---|---|---|---|
| 1 | 1.044177 | −2.405142 | −3.518511 |
| 6 | 1.26219 | −2.413714 | −0.28985 |
| 1 | 1.576882 | −3.44866 | −0.273402 |
| 6 | 0.862205 | −1.839838 | 0.93317 |
| 6 | 0.950928 | −2.724886 | 2.157498 |
| 1 | 1.700502 | −2.351769 | 2.862656 |
| 1 | −0.006758 | −2.738539 | 2.686971 |
| 1 | 1.212291 | −3.746501 | 1.877365 |
| 6 | 0.18505 | −0.101294 | 2.392814 |
| 6 | −1.101502 | −0.11027 | 2.939954 |
| 1 | −1.928153 | −0.472964 | 2.338369 |
| 6 | −1.30901 | 0.329467 | 4.249528 |
| 1 | −2.312702 | 0.31738 | 4.66592 |
| 6 | −0.23397 | 0.772761 | 5.022396 |
| 6 | 1.051247 | 0.78498 | 4.475555 |
| 1 | 1.890671 | 1.144725 | 5.063016 |
| 6 | 1.260084 | 0.353795 | 3.165725 |
| 1 | 2.255756 | 0.358246 | 2.731217 |
| 6 | 1.72552 | 3.530582 | 1.331992 |
| 1 | 1.648046 | 2.448827 | 1.456543 |
| 1 | 2.716247 | 3.756785 | 0.922583 |
| 6 | 1.504955 | 4.231278 | 2.648281 |
| 6 | 2.33938 | 5.270682 | 3.069698 |
| 1 | 3.173626 | 5.57541 | 2.441692 |
| 6 | 2.108722 | 5.920338 | 4.285756 |
| 1 | 2.765396 | 6.726811 | 4.601657 |
| 6 | 1.035449 | 5.534532 | 5.090809 |
| 1 | 0.854506 | 6.038251 | 6.036848 |
| 6 | 0.196692 | 4.494907 | 4.676623 |
| 1 | −0.634419 | 4.180224 | 5.302553 |
| 6 | 0.431592 | 3.848953 | 3.464332 |
| 1 | −0.205395 | 3.030236 | 3.147306 |
| 8 | −1.685889 | 0.297682 | −1.084485 |
| 6 | −2.105763 | −0.690214 | −1.991275 |
| 1 | −1.500287 | −0.685194 | −2.911494 |
| 1 | −3.138605 | −0.455398 | −2.297771 |
| 6 | −2.076884 | −2.093548 | −1.40304 |
| 6 | −1.688264 | −3.197573 | −2.168618 |
| 1 | −1.412742 | −3.053892 | −3.211504 |
| 6 | −1.629798 | −4.475148 | −1.605853 |
| 1 | −1.315507 | −5.320027 | −2.213668 |
| 6 | −1.956628 | −4.662734 | −0.261634 |

| | | | |
|---|---|---|---|
| 1 | −1.900048 | −5.653097 | 0.18267 |
| 6 | −2.360168 | −3.56757 | 0.508592 |
| 1 | −2.623493 | −3.705425 | 1.554715 |
| 6 | −2.425083 | −2.297288 | −0.062674 |
| 1 | −2.729495 | −1.439379 | 0.528135 |
| 6 | 0.072809 | 3.008512 | −0.338999 |
| 6 | −0.765983 | 3.707566 | −1.416491 |
| 6 | −1.503292 | 4.986129 | −1.003707 |
| 6 | −2.719004 | 4.754592 | −0.100381 |
| 6 | −2.414699 | 3.97258 | 1.184442 |
| 6 | −2.120455 | 2.487442 | 0.971932 |
| 1 | −1.455128 | 2.957712 | −1.821937 |
| 1 | −0.793905 | 5.654068 | −0.503251 |
| 1 | −3.495197 | 4.215246 | −0.6636 |
| 1 | −1.590519 | 4.438532 | 1.733668 |
| 1 | −0.04552 | 3.939476 | −2.207788 |
| 1 | −1.834079 | 5.500747 | −1.915177 |
| 1 | −3.151773 | 5.727345 | 0.16784 |
| 1 | −3.296352 | 4.016803 | 1.838334 |
| 1 | −2.266543 | 1.943984 | 1.907707 |
| 1 | −2.783254 | 2.055017 | 0.215512 |
| 8 | 0.869368 | 2.031938 | −0.850312 |
| 8 | −0.758311 | 2.164383 | 0.602382 |
| 1 | −0.396793 | 1.115519 | 6.040042 |

**Table S5.** Cartesian coordinate of **C$_{eq}$TS2a.**

| The number of atom coordinates (angstroms) | X | Y | Z |
|---|---|---|---|
| 13 | −0.046888 | 0.642566 | −0.413124 |
| 8 | 1.011547 | −0.368143 | −1.59428 |
| 8 | 0.872536 | 4.070575 | 0.316745 |
| 7 | 0.426901 | −0.567136 | 1.063742 |
| 6 | 1.329896 | −1.612257 | −1.517905 |
| 6 | 1.848996 | −2.227372 | −2.791178 |
| 1 | 2.701128 | −1.643929 | −3.157285 |
| 1 | 2.150499 | −3.269425 | −2.66154 |
| 1 | 1.068403 | −2.173556 | −3.559016 |
| 6 | 1.224365 | −2.361365 | −0.351331 |
| 1 | 1.493831 | −3.408521 | −0.391729 |
| 6 | 0.834004 | −1.834506 | 0.894155 |
| 6 | 0.892197 | −2.771332 | 2.081164 |
| 1 | 1.594056 | −2.408452 | 2.83882 |
| 1 | −0.090334 | −2.835456 | 2.559454 |

| | | | |
|---|---|---|---|
| 1 | 1.195753 | −3.772408 | 1.770422 |
| 6 | 0.238459 | −0.127215 | 2.419428 |
| 6 | −1.011126 | −0.218753 | 3.039289 |
| 1 | −1.848549 | −0.61861 | 2.47708 |
| 6 | −1.167528 | 0.192369 | 4.364767 |
| 1 | −2.143749 | 0.119649 | 4.836756 |
| 6 | −0.077091 | 0.688626 | 5.082211 |
| 6 | 1.173022 | 0.776881 | 4.465272 |
| 1 | 2.024003 | 1.173662 | 5.011081 |
| 6 | 1.329912 | 0.373042 | 3.1394 |
| 1 | 2.297955 | 0.435261 | 2.649872 |
| 6 | 1.875192 | 3.651327 | 1.285865 |
| 1 | 1.866459 | 2.5634 | 1.34958 |
| 1 | 2.847839 | 3.977345 | 0.906425 |
| 6 | 1.543932 | 4.283259 | 2.609388 |
| 6 | 2.282277 | 5.362523 | 3.103873 |
| 1 | 3.117596 | 5.752163 | 2.526355 |
| 6 | 1.953164 | 5.94091 | 4.332547 |
| 1 | 2.534 | 6.778257 | 4.710005 |
| 6 | 0.877741 | 5.44338 | 5.071303 |
| 1 | 0.620427 | 5.892312 | 6.027161 |
| 6 | 0.135553 | 4.36456 | 4.580755 |
| 1 | −0.694988 | 3.96422 | 5.155764 |
| 6 | 0.467524 | 3.7865 | 3.356925 |
| 1 | −0.091262 | 2.94059 | 2.972483 |
| 8 | −1.584973 | 0.3852 | −1.247302 |
| 6 | −1.931754 | −0.661372 | −2.109529 |
| 1 | −1.201197 | −0.783886 | −2.926091 |
| 1 | −2.892235 | −0.41085 | −2.591772 |
| 6 | −2.082286 | −1.998192 | −1.395516 |
| 6 | −1.752661 | −3.201524 | −2.028178 |
| 1 | −1.384107 | −3.184022 | −3.052246 |
| 6 | −1.866344 | −4.419756 | −1.354655 |
| 1 | −1.595142 | −5.344123 | −1.858911 |
| 6 | −2.30925 | −4.449204 | −0.030504 |
| 1 | −2.387411 | −5.394643 | 0.500058 |
| 6 | −2.651981 | −3.252913 | 0.606853 |
| 1 | −3.003771 | −3.266167 | 1.63602 |
| 6 | −2.543562 | −2.040304 | −0.074564 |
| 1 | −2.797713 | −1.105068 | 0.414792 |
| 6 | 0.356854 | 3.154228 | −0.497078 |
| 6 | −0.623698 | 3.736192 | −1.491727 |

| | | | |
|---|---|---|---|
| 6 | −1.522834 | 4.87369 | −0.997496 |
| 6 | −2.749853 | 4.435747 | −0.18537 |
| 6 | −2.466782 | 3.739731 | 1.156298 |
| 6 | −2.077917 | 2.261465 | 1.070065 |
| 1 | −1.209296 | 2.902408 | −1.890833 |
| 1 | −0.92392 | 5.580836 | −0.41216 |
| 1 | −3.375188 | 3.775498 | −0.804221 |
| 1 | −1.696559 | 4.287533 | 1.712632 |
| 1 | 0.015602 | 4.095901 | −2.310722 |
| 1 | −1.877106 | 5.421775 | −1.879869 |
| 1 | −3.352488 | 5.333217 | 0.009513 |
| 1 | −3.381957 | 3.78366 | 1.763529 |
| 1 | −2.181737 | 1.820501 | 2.070772 |
| 1 | −2.767264 | 1.73379 | 0.397331 |
| 8 | 1.047411 | 2.11252 | −0.831413 |
| 8 | −0.739306 | 2.048193 | 0.646952 |
| 1 | −0.200974 | 1.010022 | 6.112378 |

**Table S6.** Cartesian coordinate of **$C_{eq}$4a.**

| The number of atom coordinates (angstroms) | X | Y | Z |
|---|---|---|---|
| 13 | −0.193602 | 0.671856 | −0.169588 |
| 8 | 0.866953 | −0.321522 | −1.366233 |
| 8 | 1.071253 | 4.246577 | −0.049571 |
| 7 | 0.2684 | −0.625185 | 1.269638 |
| 6 | 1.347874 | −1.512337 | −1.294336 |
| 6 | 1.935609 | −2.046537 | −2.574825 |
| 1 | 2.729311 | −1.374632 | −2.920964 |
| 1 | 2.339097 | −3.055701 | −2.462314 |
| 1 | 1.159925 | −2.054973 | −3.348576 |
| 6 | 1.355944 | −2.26885 | −0.130761 |
| 1 | 1.785159 | −3.261773 | −0.168658 |
| 6 | 0.845437 | −1.820837 | 1.104563 |
| 6 | 0.980134 | −2.768855 | 2.277587 |
| 1 | 1.383475 | −2.258398 | 3.157115 |
| 1 | −0.002462 | −3.162439 | 2.558725 |
| 1 | 1.627619 | −3.610514 | 2.024156 |
| 6 | −0.18609 | −0.29417 | 2.593262 |
| 6 | −1.391937 | −0.805199 | 3.084423 |
| 1 | −1.974338 | −1.471426 | 2.45758 |
| 6 | −1.834389 | −0.457618 | 4.36201 |
| 1 | −2.773965 | −0.859305 | 4.732095 |
| 6 | −1.079921 | 0.408525 | 5.15563 |

| | | | |
|---|---|---|---|
| 6 | 0.123678 | 0.922233 | 4.663722 |
| 1 | 0.71594 | 1.604633 | 5.266651 |
| 6 | 0.56968 | 0.570088 | 3.390181 |
| 1 | 1.495534 | 0.976335 | 2.995599 |
| 6 | 2.246034 | 3.955702 | 0.775694 |
| 1 | 2.39458 | 2.875288 | 0.783816 |
| 1 | 3.09316 | 4.431295 | 0.275696 |
| 6 | 2.013858 | 4.511066 | 2.153789 |
| 6 | 2.806254 | 5.545163 | 2.660034 |
| 1 | 3.599704 | 5.968035 | 2.047943 |
| 6 | 2.583205 | 6.036378 | 3.949425 |
| 1 | 3.205047 | 6.838551 | 4.337512 |
| 6 | 1.558547 | 5.501497 | 4.732302 |
| 1 | 1.381392 | 5.886732 | 5.733033 |
| 6 | 0.760436 | 4.47041 | 4.22559 |
| 1 | −0.038792 | 4.048136 | 4.828621 |
| 6 | 0.988744 | 3.972364 | 2.943902 |
| 1 | 0.377528 | 3.173679 | 2.535318 |
| 8 | −1.723059 | 0.453531 | −1.035281 |
| 6 | −2.003659 | −0.507198 | −2.016417 |
| 1 | −1.267663 | −0.487114 | −2.836338 |
| 1 | −2.979995 | −0.263345 | −2.469575 |
| 6 | −2.069186 | −1.924997 | −1.466515 |
| 6 | −1.617599 | −3.019142 | −2.211722 |
| 1 | −1.212091 | −2.855723 | −3.208272 |
| 6 | −1.665389 | −4.313212 | −1.687287 |
| 1 | −1.299166 | −5.150041 | −2.277046 |
| 6 | −2.167397 | −4.52896 | −0.40251 |
| 1 | −2.197718 | −5.533213 | 0.012304 |
| 6 | −2.633686 | −3.44324 | 0.345588 |
| 1 | −3.035555 | −3.604058 | 1.343664 |
| 6 | −2.586485 | −2.154313 | −0.185841 |
| 1 | −2.932536 | −1.301962 | 0.390658 |
| 6 | 0.46838 | 3.268705 | −0.689681 |
| 6 | −0.74568 | 3.700383 | −1.466202 |
| 6 | −1.677526 | 4.731203 | −0.804547 |
| 6 | −2.815767 | 4.162901 | 0.06365 |
| 6 | −2.454461 | 3.638182 | 1.466754 |
| 6 | −1.982497 | 2.183539 | 1.543688 |
| 1 | −1.28704 | 2.793591 | −1.747813 |
| 1 | −1.08304 | 5.445316 | −0.222898 |
| 1 | −3.33708 | 3.373675 | −0.496466 |

| | | | |
|---|---|---|---|
| 1 | −1.693717 | 4.287527 | 1.92101 |
| 1 | −0.32843 | 4.127683 | −2.391265 |
| 1 | −2.139051 | 5.307219 | −1.616231 |
| 1 | −3.543453 | 4.976605 | 0.18748 |
| 1 | −3.347867 | 3.717255 | 2.103764 |
| 1 | −1.922057 | 1.899316 | 2.606226 |
| 1 | −2.742443 | 1.53639 | 1.077474 |
| 8 | 0.984104 | 2.131699 | −0.81132 |
| 8 | −0.729128 | 2.009388 | 0.932153 |
| 1 | −1.427117 | 0.68424 | 6.147568 |

**Table S7.** Cartesian coordinate of **C$_{ax}$1a.**

| The number of atom coordinates (angstroms) | X | Y | Z |
|---|---|---|---|
| 13 | 0.387295 | −0.037568 | −1.140796 |
| 8 | 0.725461 | −1.613658 | −2.102435 |
| 8 | 1.689917 | 0.832852 | −1.939349 |
| 7 | 0.837728 | −0.933194 | 0.570738 |
| 6 | 0.89951 | −2.816792 | −1.695564 |
| 6 | 0.944038 | −3.865575 | −2.776813 |
| 1 | 1.119747 | −4.869528 | −2.382986 |
| 1 | 1.730708 | −3.613094 | −3.496571 |
| 1 | −0.007615 | −3.855621 | −3.319922 |
| 6 | 1.053301 | −3.164078 | −0.356341 |
| 1 | 1.205322 | −4.209096 | −0.118612 |
| 6 | 1.049818 | −2.248927 | 0.713721 |
| 6 | 1.312626 | −2.827186 | 2.089416 |
| 1 | 2.058464 | −2.239105 | 2.632174 |
| 1 | 0.396534 | −2.817001 | 2.689364 |
| 1 | 1.659889 | −3.859334 | 2.014521 |
| 6 | 0.910588 | −0.121382 | 1.75344 |
| 6 | −0.13833 | −0.102906 | 2.679424 |
| 6 | −0.067828 | 0.721125 | 3.803274 |
| 1 | −0.883456 | 0.720227 | 4.521968 |
| 6 | 1.042147 | 1.546238 | 4.004659 |
| 6 | 2.087565 | 1.533304 | 3.076631 |
| 1 | 2.955024 | 2.171124 | 3.223267 |
| 6 | 2.02461 | 0.701131 | 1.95872 |
| 6 | 2.245373 | 2.090638 | −1.734692 |
| 1 | 2.477789 | 2.268861 | −0.669601 |
| 1 | 3.212709 | 2.140277 | −2.264642 |
| 6 | 1.401139 | 3.270566 | −2.206404 |
| 6 | 0.185021 | 3.096815 | −2.872988 |

| | | | |
|---|---|---|---|
| 1 | −0.176991 | 2.092481 | −3.063894 |
| 6 | −0.58036 | 4.203221 | −3.252909 |
| 1 | −1.528032 | 4.04884 | −3.763841 |
| 6 | −0.139344 | 5.49935 | −2.978013 |
| 1 | −0.736921 | 6.358331 | −3.272879 |
| 6 | 1.081447 | 5.682382 | −2.320946 |
| 1 | 1.438399 | 6.686078 | −2.101865 |
| 6 | 1.841932 | 4.575344 | −1.941748 |
| 1 | 2.787583 | 4.723447 | −1.421573 |
| 8 | −1.297226 | 0.116151 | −1.671106 |
| 6 | −2.085999 | −0.910396 | −2.200163 |
| 1 | −1.63484 | −1.362307 | −3.098463 |
| 1 | −3.047809 | −0.475217 | −2.524839 |
| 6 | −2.381943 | −2.025883 | −1.203893 |
| 6 | −2.553671 | −3.348605 | −1.627632 |
| 1 | −2.470975 | −3.58506 | −2.686938 |
| 6 | −2.813969 | −4.369114 | −0.709781 |
| 1 | −2.933105 | −5.392357 | −1.057836 |
| 6 | −2.905054 | −4.078317 | 0.653144 |
| 1 | −3.098461 | −4.87149 | 1.370892 |
| 6 | −2.743456 | −2.758767 | 1.084997 |
| 1 | −2.817625 | −2.522716 | 2.144982 |
| 6 | −2.486423 | −1.74235 | 0.163919 |
| 1 | −2.33831 | −0.719643 | 0.492689 |
| 6 | −0.973949 | 2.290821 | 0.377163 |
| 6 | −0.95223 | 3.794136 | 0.477734 |
| 6 | −0.996861 | 4.305218 | 1.936233 |
| 6 | −2.396886 | 4.29119 | 2.561886 |
| 6 | −3.049945 | 2.904911 | 2.635401 |
| 6 | −3.234965 | 2.222057 | 1.283716 |
| 1 | −1.792001 | 4.206133 | −0.096476 |
| 1 | −0.300069 | 3.715851 | 2.545909 |
| 1 | −3.050526 | 4.9592 | 1.982323 |
| 1 | −2.463532 | 2.235082 | 3.275929 |
| 1 | −0.037177 | 4.115489 | −0.02003 |
| 1 | −0.619909 | 5.33418 | 1.934181 |
| 1 | −2.341984 | 4.712318 | 3.573232 |
| 1 | −4.042929 | 2.99758 | 3.093333 |
| 1 | −3.924533 | 1.379889 | 1.359557 |
| 1 | −3.616874 | 2.9096 | 0.520999 |
| 8 | 0.002067 | 1.678161 | −0.049989 |
| 8 | −2.020495 | 1.594052 | 0.795679 |

| | | | |
|---|---|---|---|
| 1 | 2.825676 | 0.688959 | 1.225758 |
| 1 | −1.005825 | −0.72943 | 2.50219 |
| 1 | 1.094101 | 2.189824 | 4.878505 |

**Table S8.** Cartesian coordinate of **$C_{ax}$TS1a.**

| The number of atom coordinates (angstroms) | X | Y | Z |
|---|---|---|---|
| 13 | 0.647224 | 0.186784 | −0.586075 |
| 8 | 0.530446 | −1.336143 | −1.656702 |
| 8 | 1.985409 | 0.937353 | −1.434631 |
| 7 | 1.233322 | −0.865122 | 0.958452 |
| 6 | 0.590853 | −2.581132 | −1.327721 |
| 6 | 0.230114 | −3.550007 | −2.422278 |
| 1 | 0.434637 | −4.587845 | −2.148364 |
| 1 | 0.779495 | −3.29484 | −3.334635 |
| 1 | −0.839916 | −3.445876 | −2.639364 |
| 6 | 0.939814 | −3.033743 | −0.060347 |
| 1 | 0.966657 | −4.103316 | 0.104992 |
| 6 | 1.292003 | −2.196162 | 1.02099 |
| 6 | 1.758797 | −2.866131 | 2.295069 |
| 1 | 2.804041 | −2.611678 | 2.50107 |
| 1 | 1.176642 | −2.516357 | 3.153716 |
| 1 | 1.671247 | −3.95187 | 2.222784 |
| 6 | 1.741235 | −0.107794 | 2.071406 |
| 6 | 0.889273 | 0.320677 | 3.094125 |
| 6 | 1.398923 | 1.07163 | 4.152485 |
| 1 | 0.732461 | 1.403669 | 4.944328 |
| 6 | 2.754707 | 1.410702 | 4.190635 |
| 6 | 3.599525 | 0.999937 | 3.158005 |
| 1 | 4.652281 | 1.269252 | 3.172537 |
| 6 | 3.09453 | 0.242732 | 2.098359 |
| 6 | 2.463066 | 2.233074 | −1.175856 |
| 1 | 2.569358 | 2.426541 | −0.097071 |
| 1 | 3.47428 | 2.317566 | −1.608798 |
| 6 | 1.592779 | 3.325784 | −1.775654 |
| 6 | 0.937899 | 3.117748 | −2.995831 |
| 1 | 1.052616 | 2.158145 | −3.491101 |
| 6 | 0.128645 | 4.11179 | −3.549044 |
| 1 | −0.379107 | 3.932013 | −4.493762 |
| 6 | −0.03522 | 5.33369 | −2.889539 |
| 1 | −0.669801 | 6.106303 | −3.316192 |
| 6 | 0.619043 | 5.551798 | −1.674181 |
| 1 | 0.49084 | 6.494533 | −1.147659 |

| | | | |
|---|---|---|---|
| 6 | 1.424447 | 4.552237 | −1.123221 |
| 1 | 1.90906 | 4.716099 | −0.16305 |
| 8 | −1.173883 | 0.511538 | −0.950221 |
| 6 | −2.215288 | −0.11731 | −1.666866 |
| 1 | −1.883083 | −0.259801 | −2.702416 |
| 1 | −3.066713 | 0.578578 | −1.700999 |
| 6 | −2.661443 | −1.449088 | −1.090937 |
| 6 | −3.367815 | −2.342124 | −1.908109 |
| 1 | −3.572662 | −2.075499 | −2.943397 |
| 6 | −3.799579 | −3.573026 | −1.412538 |
| 1 | −4.341518 | −4.256689 | −2.060919 |
| 6 | −3.523687 | −3.930131 | −0.089471 |
| 1 | −3.850534 | −4.892027 | 0.296403 |
| 6 | −2.819638 | −3.045186 | 0.728007 |
| 1 | −2.592158 | −3.317082 | 1.755679 |
| 6 | −2.393575 | −1.810567 | 0.232531 |
| 1 | −1.841972 | −1.127327 | 0.867783 |
| 6 | −1.18265 | 1.732969 | 0.327608 |
| 6 | −1.761716 | 2.949608 | −0.369087 |
| 6 | −2.017835 | 4.107816 | 0.614747 |
| 6 | −3.373862 | 4.012142 | 1.330856 |
| 6 | −3.555625 | 2.772408 | 2.219479 |
| 6 | −3.302225 | 1.440403 | 1.515503 |
| 1 | −2.690783 | 2.691142 | −0.889583 |
| 1 | −1.195893 | 4.153419 | 1.34065 |
| 1 | −4.168015 | 4.018815 | 0.569513 |
| 1 | −2.879776 | 2.819483 | 3.083265 |
| 1 | −1.034617 | 3.236062 | −1.126145 |
| 1 | −1.980239 | 5.04524 | 0.048401 |
| 1 | −3.527195 | 4.910025 | 1.942898 |
| 1 | −4.57947 | 2.759764 | 2.616386 |
| 1 | −3.652458 | 0.604773 | 2.126324 |
| 1 | −3.815664 | 1.380091 | 0.549167 |
| 8 | 0.094872 | 1.657811 | 0.508864 |
| 8 | −1.890412 | 1.190376 | 1.360838 |
| 1 | 3.736639 | −0.075266 | 1.281983 |
| 1 | −0.168842 | 0.093213 | 3.027341 |
| 1 | 3.146803 | 2.00018 | 5.014987 |

**Table S9.** Cartesian coordinate of **C$_{ax}$2a.**

| The number of atom coordinates (angstroms) | X | Y | Z |
|---|---|---|---|
| 13 | 0.612868 | 0.286765 | −0.441541 |

| | | | |
|---|---|---|---|
| 8 | 0.388998 | −1.148796 | −1.588305 |
| 8 | 2.000821 | 0.972637 | −1.270174 |
| 7 | 1.045595 | −0.869942 | 1.064846 |
| 6 | 0.736472 | −2.380008 | −1.425132 |
| 6 | 0.548461 | −3.258459 | −2.631949 |
| 1 | 0.904825 | −4.278211 | −2.467987 |
| 1 | 1.07533 | −2.820595 | −3.486974 |
| 1 | −0.518356 | −3.286327 | −2.880048 |
| 6 | 1.221946 | −2.898162 | −0.228922 |
| 1 | 1.485345 | −3.948014 | −0.204093 |
| 6 | 1.327832 | −2.171622 | 0.976977 |
| 6 | 1.768733 | −2.929995 | 2.209697 |
| 1 | 2.560757 | −2.388465 | 2.736335 |
| 1 | 0.937007 | −3.036242 | 2.914317 |
| 1 | 2.128669 | −3.926782 | 1.947336 |
| 6 | 1.091005 | −0.230861 | 2.351014 |
| 6 | 0.035421 | −0.387325 | 3.254905 |
| 6 | 0.056523 | 0.283421 | 4.477177 |
| 1 | −0.769252 | 0.158725 | 5.172846 |
| 6 | 1.126354 | 1.122238 | 4.800813 |
| 6 | 2.176625 | 1.283176 | 3.894135 |
| 1 | 3.009661 | 1.938101 | 4.134909 |
| 6 | 2.16091 | 0.608897 | 2.672159 |
| 6 | 2.271615 | 2.344937 | −1.364735 |
| 1 | 2.291606 | 2.829531 | −0.375446 |
| 1 | 3.279502 | 2.470868 | −1.795206 |
| 6 | 1.278149 | 3.101571 | −2.237754 |
| 6 | 0.538577 | 2.437538 | −3.223516 |
| 1 | 0.686394 | 1.370599 | −3.359398 |
| 6 | −0.388899 | 3.129428 | −4.005876 |
| 1 | −0.960847 | 2.596559 | −4.762038 |
| 6 | −0.585491 | 4.499896 | −3.81841 |
| 1 | −1.312149 | 5.037846 | −4.421869 |
| 6 | 0.159334 | 5.173845 | −2.846366 |
| 1 | 0.011285 | 6.239275 | −2.687773 |
| 6 | 1.081827 | 4.476866 | −2.06434 |
| 1 | 1.640615 | 5.002784 | −1.292641 |
| 8 | −1.322258 | 0.728285 | −0.821903 |
| 6 | −2.553839 | 0.193972 | −1.343993 |
| 1 | −2.589003 | 0.434532 | −2.410726 |
| 1 | −3.365751 | 0.72377 | −0.837416 |
| 6 | −2.670438 | −1.294496 | −1.117481 |

| | | | |
|---|---|---|---|
| 6 | −3.074814 | −2.134839 | −2.158741 |
| 1 | −3.281231 | −1.714566 | −3.140568 |
| 6 | −3.201817 | −3.511519 | −1.951635 |
| 1 | −3.516185 | −4.154142 | −2.769873 |
| 6 | −2.906953 | −4.05951 | −0.702311 |
| 1 | −2.989537 | −5.131271 | −0.542819 |
| 6 | −2.501307 | −3.222091 | 0.341452 |
| 1 | −2.266976 | −3.642981 | 1.315951 |
| 6 | −2.395195 | −1.847339 | 0.139885 |
| 1 | −2.08481 | −1.188331 | 0.94335 |
| 6 | −1.291087 | 1.74901 | 0.265212 |
| 6 | −1.798661 | 3.098739 | −0.255731 |
| 6 | −1.265888 | 4.280969 | 0.563641 |
| 6 | −1.830269 | 4.383554 | 1.985358 |
| 6 | −1.687818 | 3.104036 | 2.825492 |
| 6 | −2.580907 | 1.950609 | 2.358696 |
| 1 | −2.896162 | 3.095449 | −0.282152 |
| 1 | −0.176192 | 4.187001 | 0.603127 |
| 1 | −2.897379 | 4.64962 | 1.935912 |
| 1 | −0.648278 | 2.76223 | 2.855812 |
| 1 | −1.437628 | 3.190164 | −1.283017 |
| 1 | −1.48475 | 5.207147 | 0.018098 |
| 1 | −1.331445 | 5.212373 | 2.504795 |
| 1 | −1.982996 | 3.328687 | 3.859408 |
| 1 | −2.695384 | 1.2247 | 3.169529 |
| 1 | −3.582674 | 2.336839 | 2.112345 |
| 8 | 0.019363 | 1.723214 | 0.548321 |
| 8 | −2.103192 | 1.160166 | 1.262454 |
| 1 | 2.962655 | 0.739429 | 1.951059 |
| 1 | −0.805197 | −1.017584 | 2.982551 |
| 1 | 1.138075 | 1.650526 | 5.750097 |

**Table S10.** Cartesian coordinate of **C$_{ax}$3a.**

| The number of atom coordinates (angstroms) | X | Y | Z |
|---|---|---|---|
| 13 | 0.159196 | 0.80063 | 0.64081 |
| 8 | −0.514693 | −0.478277 | −0.455646 |
| 8 | 1.375432 | 1.799189 | −0.113941 |
| 7 | 1.280699 | −0.477859 | 1.660154 |
| 6 | −0.385476 | −1.777748 | −0.373589 |
| 6 | −1.212316 | −2.536768 | −1.375514 |
| 1 | −1.076447 | −3.618072 | −1.29968 |
| 1 | −0.948803 | −2.209458 | −2.388144 |

| | | | |
|---|---|---|---|
| 1 | −2.271922 | −2.295743 | −1.227355 |
| 6 | 0.43044 | −2.424692 | 0.528159 |
| 1 | 0.45967 | −3.506829 | 0.491402 |
| 6 | 1.281187 | −1.784442 | 1.483624 |
| 6 | 2.204868 | −2.684756 | 2.272672 |
| 1 | 3.175653 | −2.750747 | 1.767233 |
| 1 | 2.382993 | −2.299443 | 3.279004 |
| 1 | 1.791431 | −3.694175 | 2.338431 |
| 6 | 2.220044 | 0.137554 | 2.555867 |
| 6 | 1.751766 | 0.807459 | 3.692621 |
| 6 | 2.656801 | 1.45608 | 4.534879 |
| 1 | 2.288399 | 1.9716 | 5.417803 |
| 6 | 4.023153 | 1.449808 | 4.245657 |
| 6 | 4.48346 | 0.790798 | 3.102991 |
| 1 | 5.542183 | 0.791562 | 2.85753 |
| 6 | 3.587718 | 0.137826 | 2.257859 |
| 6 | 1.986861 | 1.584722 | −1.36464 |
| 1 | 1.279722 | 1.14906 | −2.089965 |
| 1 | 2.298741 | 2.558536 | −1.772247 |
| 6 | 3.199002 | 0.677561 | −1.250539 |
| 6 | 4.466629 | 1.204829 | −0.978974 |
| 1 | 4.587484 | 2.282774 | −0.899532 |
| 6 | 5.567795 | 0.36528 | −0.797157 |
| 1 | 6.545715 | 0.791458 | −0.58811 |
| 6 | 5.411652 | −1.021364 | −0.878207 |
| 1 | 6.266413 | −1.677092 | −0.734449 |
| 6 | 4.149585 | −1.559606 | −1.148333 |
| 1 | 4.017841 | −2.636814 | −1.215522 |
| 6 | 3.055404 | −0.712877 | −1.336146 |
| 1 | 2.075649 | −1.136645 | −1.539504 |
| 8 | −1.837921 | 3.446683 | 1.983799 |
| 6 | −0.628644 | 4.208545 | 1.810912 |
| 1 | 0.248471 | 3.562194 | 1.897546 |
| 1 | −0.618785 | 4.904428 | 2.656827 |
| 6 | −0.626534 | 4.968583 | 0.502444 |
| 6 | 0.418929 | 4.826602 | −0.415389 |
| 1 | 1.222871 | 4.131958 | −0.195103 |
| 6 | 0.397211 | 5.529173 | −1.62343 |
| 1 | 1.212805 | 5.40683 | −2.332082 |
| 6 | −0.671171 | 6.375847 | −1.92674 |
| 1 | −0.688797 | 6.919046 | −2.868211 |
| 6 | −1.720593 | 6.519561 | −1.013606 |

| | | | |
|---|---|---|---|
| 1 | −2.556234 | 7.176268 | −1.242673 |
| 6 | −1.694439 | 5.821236 | 0.193565 |
| 1 | −2.514457 | 5.921317 | 0.900639 |
| 6 | −1.812817 | 2.121811 | 1.533723 |
| 6 | −3.211275 | 1.557964 | 1.814349 |
| 6 | −3.607103 | 0.348934 | 0.952448 |
| 6 | −4.169711 | 0.722176 | −0.433216 |
| 6 | −3.155663 | 1.293301 | −1.436473 |
| 6 | −2.358245 | 2.482819 | −0.91466 |
| 1 | −3.944683 | 2.36288 | 1.691013 |
| 1 | −2.750995 | −0.326454 | 0.843038 |
| 1 | −4.984978 | 1.447396 | −0.292124 |
| 1 | −2.436846 | 0.517026 | −1.719932 |
| 1 | −3.206479 | 1.291735 | 2.875954 |
| 1 | −4.380298 | −0.212667 | 1.49103 |
| 1 | −4.629295 | −0.169642 | −0.880115 |
| 1 | −3.68269 | 1.603425 | −2.349294 |
| 1 | −1.73223 | 2.926579 | −1.693741 |
| 1 | −2.994646 | 3.276112 | −0.508557 |
| 8 | −0.797757 | 1.366303 | 2.053181 |
| 8 | −1.448999 | 2.067293 | 0.116237 |
| 1 | 3.937471 | −0.354791 | 1.359026 |
| 1 | 0.686861 | 0.828555 | 3.892877 |
| 1 | 4.72274 | 1.961372 | 4.901153 |

**Table S11.** Cartesian coordinate of **$C_{ax}$TS2a.**

| The number of atom coordinates (angstroms) | X | Y | Z |
|---|---|---|---|
| 13 | 0.462917 | 1.307651 | 0.230532 |
| 8 | 0.098226 | 0.308616 | −1.318268 |
| 8 | 1.883042 | 2.179051 | −0.335444 |
| 7 | 0.874177 | −0.341791 | 1.239529 |
| 6 | −0.086877 | −0.957265 | −1.468944 |
| 6 | −0.586445 | −1.374254 | −2.82801 |
| 1 | −0.707931 | −2.456203 | −2.919433 |
| 1 | 0.11342 | −1.022352 | −3.594678 |
| 1 | −1.547959 | −0.885438 | −3.023863 |
| 6 | 0.175671 | −1.895639 | −0.479307 |
| 1 | 0.028191 | −2.94125 | −0.718654 |
| 6 | 0.718713 | −1.587128 | 0.793641 |
| 6 | 1.173361 | −2.755112 | 1.641721 |
| 1 | 2.248771 | −2.670346 | 1.834436 |
| 1 | 0.677539 | −2.762177 | 2.616571 |

| | | | |
|---|---|---|---|
| 1 | 0.979698 | −3.704288 | 1.138696 |
| 6 | 1.5046 | −0.159899 | 2.525235 |
| 6 | 0.766202 | −0.273973 | 3.708033 |
| 6 | 1.385436 | −0.072716 | 4.941162 |
| 1 | 0.803735 | −0.163222 | 5.854846 |
| 6 | 2.744148 | 0.24856 | 5.002395 |
| 6 | 3.477044 | 0.367072 | 3.820446 |
| 1 | 4.5337 | 0.618982 | 3.852682 |
| 6 | 2.861997 | 0.165265 | 2.583453 |
| 6 | 2.737982 | 1.802356 | −1.391916 |
| 1 | 2.177703 | 1.361211 | −2.230168 |
| 1 | 3.237108 | 2.706122 | −1.77464 |
| 6 | 3.788682 | 0.814564 | −0.918229 |
| 6 | 4.947201 | 1.264267 | −0.272018 |
| 1 | 5.120981 | 2.333953 | −0.17978 |
| 6 | 5.862169 | 0.359375 | 0.270348 |
| 1 | 6.7551 | 0.725261 | 0.770989 |
| 6 | 5.626869 | −1.015412 | 0.175199 |
| 1 | 6.334294 | −1.721515 | 0.602055 |
| 6 | 4.478343 | −1.475659 | −0.473647 |
| 1 | 4.287643 | −2.543152 | −0.553853 |
| 6 | 3.570188 | −0.564998 | −1.017992 |
| 1 | 2.670982 | −0.929022 | −1.506679 |
| 8 | −1.032516 | 4.174548 | 1.637917 |
| 6 | −0.058366 | 4.856592 | 0.797392 |
| 1 | 0.868762 | 4.283096 | 0.762169 |
| 1 | 0.121645 | 5.80659 | 1.308814 |
| 6 | −0.639873 | 5.072245 | −0.579053 |
| 6 | 0.006469 | 4.569793 | −1.712743 |
| 1 | 0.920561 | 3.998206 | −1.587385 |
| 6 | −0.54365 | 4.764875 | −2.981614 |
| 1 | −0.035649 | 4.365331 | −3.855606 |
| 6 | −1.747037 | 5.458141 | −3.126518 |
| 1 | −2.177224 | 5.606093 | −4.113755 |
| 6 | −2.398966 | 5.959816 | −1.995876 |
| 1 | −3.336137 | 6.50031 | −2.101904 |
| 6 | −1.84474 | 5.769947 | −0.729812 |
| 1 | −2.353714 | 6.152533 | 0.15156 |
| 6 | −0.884023 | 2.860597 | 1.851452 |
| 6 | −2.0169 | 2.333883 | 2.711651 |
| 6 | −2.529387 | 0.913441 | 2.413297 |
| 6 | −3.624951 | 0.799492 | 1.328743 |

| | | | |
|---|---|---|---|
| 6 | −3.147394 | 0.682693 | −0.127213 |
| 6 | −2.366253 | 1.89053 | −0.635567 |
| 1 | −2.843698 | 3.049475 | 2.675423 |
| 1 | −1.687885 | 0.258841 | 2.164435 |
| 1 | −4.310892 | 1.654689 | 1.419603 |
| 1 | −2.509007 | −0.206511 | −0.226297 |
| 1 | −1.608562 | 2.363036 | 3.730773 |
| 1 | −2.948273 | 0.52932 | 3.351959 |
| 1 | −4.225442 | −0.091987 | 1.552813 |
| 1 | −4.01936 | 0.522571 | −0.777432 |
| 1 | −2.095841 | 1.759116 | −1.691297 |
| 1 | −2.970245 | 2.806774 | −0.562679 |
| 8 | 0.274404 | 2.316476 | 1.869548 |
| 8 | −1.193358 | 2.082638 | 0.119444 |
| 1 | 3.424754 | 0.262118 | 1.664456 |
| 1 | −0.289573 | −0.519208 | 3.656953 |
| 1 | 3.224704 | 0.408119 | 5.963822 |

**Table S12.** Cartesian coordinate of **$C_{ax}$4a.**

| The number of atom coordinates (angstroms) | X | Y | Z |
|---|---|---|---|
| 13 | 0.414909 | 1.108852 | −0.056746 |
| 8 | 0.126301 | 0.107604 | −1.622915 |
| 8 | 1.787686 | 2.055161 | −0.645044 |
| 7 | 0.900209 | −0.515651 | 0.963373 |
| 6 | 0.134185 | −1.162207 | −1.818418 |
| 6 | −0.250026 | −1.602969 | −3.207588 |
| 1 | −0.237697 | −2.689251 | −3.324841 |
| 1 | 0.43908 | −1.154239 | −3.932637 |
| 1 | −1.251561 | −1.224737 | −3.441961 |
| 6 | 0.495537 | −2.086785 | −0.845126 |
| 1 | 0.504236 | −3.134158 | −1.119336 |
| 6 | 0.910446 | −1.75595 | 0.466254 |
| 6 | 1.429268 | −2.898974 | 1.313887 |
| 1 | 2.463031 | −2.694814 | 1.613733 |
| 1 | 0.84913 | −3.013915 | 2.233974 |
| 1 | 1.401942 | −3.839413 | 0.760459 |
| 6 | 1.370982 | −0.350736 | 2.316013 |
| 6 | 0.510418 | −0.580254 | 3.393905 |
| 6 | 0.958326 | −0.385534 | 4.70131 |
| 1 | 0.282635 | −0.565652 | 5.533507 |
| 6 | 2.268294 | 0.03982 | 4.938165 |
| 6 | 3.1264 | 0.262686 | 3.858854 |

| | | | |
|---|---|---|---|
| 1 | 4.147752 | 0.592039 | 4.030792 |
| 6 | 2.682664 | 0.069298 | 2.550115 |
| 6 | 2.741512 | 1.647241 | −1.59731 |
| 1 | 2.281538 | 1.073339 | −2.416244 |
| 1 | 3.191767 | 2.546838 | −2.045436 |
| 6 | 3.832733 | 0.810616 | −0.953066 |
| 6 | 4.838282 | 1.423337 | −0.193358 |
| 1 | 4.862702 | 2.508439 | −0.12469 |
| 6 | 5.790064 | 0.65937 | 0.484211 |
| 1 | 6.563695 | 1.150462 | 1.069238 |
| 6 | 5.745266 | −0.736811 | 0.414973 |
| 1 | 6.481947 | −1.333954 | 0.94596 |
| 6 | 4.749192 | −1.3583 | −0.341475 |
| 1 | 4.706771 | −2.44312 | −0.401604 |
| 6 | 3.803102 | −0.586953 | −1.021426 |
| 1 | 3.021285 | −1.077234 | −1.595567 |
| 8 | −0.563786 | 4.176819 | 1.738593 |
| 6 | 0.194883 | 4.707425 | 0.60107 |
| 1 | 1.029734 | 4.04439 | 0.372504 |
| 1 | 0.570049 | 5.668262 | 0.964649 |
| 6 | −0.72841 | 4.880207 | −0.577731 |
| 6 | −0.416787 | 4.296202 | −1.809163 |
| 1 | 0.475042 | 3.682941 | −1.891275 |
| 6 | −1.269266 | 4.465537 | −2.901893 |
| 1 | −1.021988 | 4.001961 | −3.853286 |
| 6 | −2.443316 | 5.210029 | −2.769413 |
| 1 | −3.109741 | 5.334954 | −3.618949 |
| 6 | −2.762383 | 5.789444 | −1.537831 |
| 1 | −3.676225 | 6.367336 | −1.42698 |
| 6 | −1.904765 | 5.629148 | −0.449344 |
| 1 | −2.153076 | 6.07491 | 0.510699 |
| 6 | −0.37045 | 2.929892 | 2.131434 |
| 6 | −1.339008 | 2.500347 | 3.203841 |
| 6 | −2.404252 | 1.518792 | 2.661947 |
| 6 | −3.413317 | 2.121853 | 1.647638 |
| 6 | −3.546964 | 1.290558 | 0.353889 |
| 6 | −2.428272 | 1.614432 | −0.652116 |
| 1 | −1.815254 | 3.383676 | 3.638739 |
| 1 | −1.880675 | 0.678314 | 2.202852 |
| 1 | −3.111469 | 3.141214 | 1.377594 |
| 1 | −3.527338 | 0.221322 | 0.610375 |
| 1 | −0.747992 | 1.99584 | 3.975499 |

| | | | |
|---|---|---|---|
| 1 | −2.938545 | 1.11857 | 3.532863 |
| 1 | −4.394714 | 2.21006 | 2.12991 |
| 1 | −4.517912 | 1.478842 | −0.12525 |
| 1 | −2.388627 | 0.841001 | −1.437015 |
| 1 | −2.666979 | 2.567111 | −1.15207 |
| 8 | 0.539424 | 2.205232 | 1.709923 |
| 8 | −1.211504 | 1.753457 | 0.014495 |
| 1 | 3.339651 | 0.25103 | 1.708657 |
| 1 | −0.50722 | −0.905266 | 3.199336 |
| 1 | 2.616459 | 0.194385 | 5.955793 |

**Table S13.** Cartesian coordinate of **C$_{eq}$1b.**

| The number of atom coordinates (angstroms) | X | Y | Z |
|---|---|---|---|
| 13 | −0.302745 | 0.335164 | −0.669722 |
| 8 | 1.181754 | 0.209368 | −1.83432 |
| 8 | −1.351459 | 0.909236 | 0.638086 |
| 7 | 0.783293 | −0.689489 | 0.665764 |
| 6 | 2.556018 | −0.980112 | −3.348434 |
| 1 | 3.153783 | −0.080904 | −3.537394 |
| 1 | 3.196618 | −1.862343 | −3.423678 |
| 1 | 1.787495 | −1.024216 | −4.128934 |
| 6 | 1.884526 | −0.86087 | −2.005657 |
| 6 | 2.061031 | −1.828703 | −1.024498 |
| 1 | 2.682746 | −2.685576 | −1.251971 |
| 6 | 1.606926 | −1.673412 | 0.306562 |
| 6 | 2.095525 | −2.668676 | 1.335556 |
| 1 | 1.242306 | −3.134312 | 1.840135 |
| 1 | 2.702824 | −3.448072 | 0.870821 |
| 1 | 2.692808 | −2.173769 | 2.108257 |
| 6 | 0.542492 | −0.501676 | 2.067323 |
| 6 | −0.568227 | −1.076671 | 2.69325 |
| 1 | −1.258692 | −1.669814 | 2.108853 |
| 6 | −0.79925 | −0.862086 | 4.049693 |
| 1 | −1.66476 | −1.300455 | 4.534813 |
| 6 | 0.084836 | −0.062031 | 4.776425 |
| 6 | 1.196312 | 0.519221 | 4.17154 |
| 1 | 1.871456 | 1.14285 | 4.7476 |
| 6 | 1.418954 | 0.290958 | 2.812342 |
| 1 | 2.277069 | 0.738928 | 2.320282 |
| 6 | −2.355566 | 1.877351 | 0.622271 |
| 1 | −2.529133 | 2.303951 | −0.379186 |
| 1 | −3.312215 | 1.413578 | 0.926597 |

| | | | |
|---|---|---|---|
| 6 | −2.050571 | 3.012384 | 1.587407 |
| 6 | −1.45109 | 2.724374 | 2.821192 |
| 1 | −1.196811 | 1.695771 | 3.047518 |
| 6 | −1.162237 | 3.742862 | 3.729369 |
| 1 | −0.693197 | 3.498426 | 4.679559 |
| 6 | −1.474749 | 5.070658 | 3.418977 |
| 1 | −1.2511 | 5.866507 | 4.124882 |
| 6 | −2.078731 | 5.366584 | 2.194503 |
| 1 | −2.323743 | 6.39594 | 1.942959 |
| 6 | −2.358609 | 4.342346 | 1.283318 |
| 1 | −2.810252 | 4.581351 | 0.322558 |
| 8 | −1.331069 | −0.638076 | −1.721293 |
| 6 | −2.584379 | −1.126575 | −1.321452 |
| 1 | −3.100367 | −1.524938 | −2.21181 |
| 1 | −3.234447 | −0.332947 | −0.919416 |
| 6 | −2.494416 | −2.238957 | −0.285515 |
| 6 | −1.429272 | −3.148367 | −0.319877 |
| 1 | −0.663558 | −3.026821 | −1.079705 |
| 6 | −1.331723 | −4.168825 | 0.627251 |
| 1 | −0.494919 | −4.862514 | 0.589687 |
| 6 | −2.301906 | −4.298038 | 1.626034 |
| 1 | −2.222809 | −5.087523 | 2.368863 |
| 6 | −3.370646 | −3.399121 | 1.665685 |
| 1 | −4.12561 | −3.483866 | 2.443499 |
| 6 | −3.462 | −2.377081 | 0.716423 |
| 1 | −4.284382 | −1.666195 | 0.766127 |
| 17 | −0.222199 | 0.23614 | 6.489666 |
| 6 | 0.592748 | 3.156412 | −0.987995 |
| 6 | 1.849685 | 2.934461 | −0.182567 |
| 6 | 1.798445 | 3.594157 | 1.214803 |
| 6 | 2.072418 | 5.102301 | 1.201778 |
| 6 | 1.102273 | 5.923412 | 0.343446 |
| 6 | 1.076916 | 5.539899 | −1.131935 |
| 1 | 2.707732 | 3.317232 | −0.750442 |
| 1 | 0.832293 | 3.378782 | 1.682524 |
| 1 | 3.097586 | 5.275204 | 0.8423 |
| 1 | 0.082669 | 5.845504 | 0.736379 |
| 1 | 1.98166 | 1.855211 | −0.097812 |
| 1 | 2.558447 | 3.103935 | 1.833328 |
| 1 | 2.03404 | 5.475556 | 2.231853 |
| 1 | 1.38576 | 6.982434 | 0.395829 |
| 1 | 0.588353 | 6.311029 | −1.728762 |

| | | | |
|---|---|---|---|
| 1 | 2.081776 | 5.376123 | −1.537778 |
| 8 | −0.188782 | 2.247601 | −1.284053 |
| 8 | 0.257359 | 4.365822 | −1.403979 |

**Table S14.** Cartesian coordinate of **C$_{eq}$TS1b.**

| The number of atom coordinates (angstroms) | X | Y | Z |
|---|---|---|---|
| 13 | −0.078761 | 0.356129 | −0.747755 |
| 8 | 0.37412 | −0.96892 | −2.002622 |
| 8 | −0.213659 | 2.054243 | 0.168164 |
| 7 | 0.756007 | −0.636903 | 0.729715 |
| 6 | 1.319261 | −1.830513 | −1.915613 |
| 6 | 1.651937 | −2.556921 | −3.193516 |
| 1 | 0.752215 | −3.060509 | −3.565328 |
| 1 | 1.947983 | −1.82691 | −3.955213 |
| 1 | 2.450191 | −3.292103 | −3.06688 |
| 6 | 1.983614 | −2.13812 | −0.72657 |
| 1 | 2.746746 | −2.905333 | −0.762071 |
| 6 | 1.639472 | −1.629284 | 0.541447 |
| 6 | 2.294435 | −2.283106 | 1.73972 |
| 1 | 1.537451 | −2.752334 | 2.37759 |
| 1 | 3.007607 | −3.047428 | 1.426668 |
| 1 | 2.815427 | −1.546431 | 2.359201 |
| 6 | 0.383354 | −0.337556 | 2.086067 |
| 6 | −0.745559 | −0.951019 | 2.639084 |
| 1 | −1.319658 | −1.645905 | 2.03855 |
| 6 | −1.135387 | −0.677387 | 3.950428 |
| 1 | −2.017815 | −1.14908 | 4.368995 |
| 6 | −0.376457 | 0.208757 | 4.712185 |
| 6 | 0.757768 | 0.824727 | 4.184066 |
| 1 | 1.33328 | 1.519255 | 4.786072 |
| 6 | 1.130158 | 0.548467 | 2.869847 |
| 1 | 2.008117 | 1.024708 | 2.452317 |
| 6 | −1.338657 | 2.916251 | 0.08026 |
| 1 | −1.121807 | 3.713092 | −0.643863 |
| 1 | −2.184049 | 2.359233 | −0.336977 |
| 6 | −1.730645 | 3.527532 | 1.408283 |
| 6 | −1.670555 | 2.775113 | 2.586504 |
| 1 | −1.303126 | 1.758632 | 2.539913 |
| 6 | −2.069421 | 3.325258 | 3.805057 |
| 1 | −2.009547 | 2.727237 | 4.710269 |
| 6 | −2.544062 | 4.638577 | 3.860557 |
| 1 | −2.853626 | 5.068817 | 4.809468 |

| | | | |
|---|---|---|---|
| 6 | −2.61729 | 5.395302 | 2.689031 |
| 1 | −2.984144 | 6.418137 | 2.721687 |
| 6 | −2.210667 | 4.841706 | 1.472485 |
| 1 | −2.269248 | 5.436831 | 0.562953 |
| 8 | −1.800012 | 0.109584 | −1.008527 |
| 6 | −2.849478 | −0.105625 | −0.118927 |
| 1 | −3.786547 | 0.274137 | −0.561792 |
| 1 | −2.722166 | 0.453367 | 0.826947 |
| 6 | −3.0553 | −1.571219 | 0.244951 |
| 6 | −2.323973 | −2.585421 | −0.380349 |
| 1 | −1.619714 | −2.315402 | −1.159575 |
| 6 | −2.489609 | −3.919004 | 0.006143 |
| 1 | −1.908855 | −4.696775 | −0.484232 |
| 6 | −3.391289 | −4.254726 | 1.017258 |
| 1 | −3.515879 | −5.291072 | 1.320419 |
| 6 | −4.133933 | −3.245582 | 1.639896 |
| 1 | −4.83878 | −3.495751 | 2.429117 |
| 6 | −3.965078 | −1.915732 | 1.254792 |
| 1 | −4.532438 | −1.131415 | 1.75358 |
| 17 | −0.864037 | 0.568833 | 6.36814 |
| 6 | 1.295304 | 2.524194 | −0.924381 |
| 6 | 2.376546 | 2.657922 | 0.13459 |
| 6 | 2.113523 | 3.585707 | 1.328231 |
| 6 | 2.212188 | 5.080987 | 1.015446 |
| 6 | 1.225659 | 5.556293 | −0.055152 |
| 6 | 1.437962 | 4.926054 | −1.428463 |
| 1 | 3.290647 | 2.96144 | −0.398427 |
| 1 | 1.127917 | 3.348588 | 1.739286 |
| 1 | 3.234985 | 5.319121 | 0.686986 |
| 1 | 0.194726 | 5.385416 | 0.271464 |
| 1 | 2.55196 | 1.63745 | 0.479238 |
| 1 | 2.851149 | 3.344036 | 2.104366 |
| 1 | 2.040127 | 5.6498 | 1.937264 |
| 1 | 1.336025 | 6.640434 | −0.190375 |
| 1 | 0.916637 | 5.50013 | −2.196612 |
| 1 | 2.503943 | 4.902155 | −1.688232 |
| 8 | 1.22422 | 1.409398 | −1.581633 |

**Table S15.** Cartesian coordinate of **C$_{eq}$2b.**

| The number of atom coordinates (angstroms) | X | Y | Z |
|---|---|---|---|
| 13 | 0.429777 | 0.221383 | −0.789434 |
| 8 | 1.533395 | −0.6408 | −2.002618 |

| | | | |
|---|---|---|---|
| 8 | −0.282841 | 1.670875 | 0.626927 |
| 7 | 0.839918 | −1.047361 | 0.638044 |
| 6 | 1.778312 | −1.903629 | −2.019631 |
| 6 | 2.340864 | −2.434875 | −3.31101 |
| 1 | 1.623347 | −2.248406 | −4.118275 |
| 1 | 3.254203 | −1.882864 | −3.560345 |
| 1 | 2.565012 | −3.503201 | −3.265925 |
| 6 | 1.582644 | −2.742909 | −0.923163 |
| 1 | 1.837155 | −3.788885 | −1.037974 |
| 6 | 1.218044 | −2.304064 | 0.368065 |
| 6 | 1.2741 | −3.326673 | 1.481392 |
| 1 | 0.276117 | −3.474031 | 1.907829 |
| 1 | 1.643771 | −4.285207 | 1.113269 |
| 1 | 1.923386 | −2.987253 | 2.294647 |
| 6 | 0.63744 | −0.702769 | 2.017849 |
| 6 | −0.586695 | −0.947177 | 2.647824 |
| 1 | −1.400468 | −1.385471 | 2.084329 |
| 6 | −0.758778 | −0.643518 | 3.99806 |
| 1 | −1.708326 | −0.833757 | 4.486379 |
| 6 | 0.300671 | −0.082686 | 4.710349 |
| 6 | 1.522042 | 0.188643 | 4.093208 |
| 1 | 2.331819 | 0.638027 | 4.657772 |
| 6 | 1.683462 | −0.123722 | 2.743133 |
| 1 | 2.626955 | 0.07676 | 2.244844 |
| 6 | −1.628189 | 2.073412 | 0.967192 |
| 1 | −2.138654 | 2.421739 | 0.068244 |
| 1 | −2.088495 | 1.138437 | 1.298379 |
| 6 | −1.682093 | 3.116768 | 2.051494 |
| 6 | −1.031428 | 2.900814 | 3.271299 |
| 1 | −0.478177 | 1.982239 | 3.418474 |
| 6 | −1.067986 | 3.867211 | 4.27479 |
| 1 | −0.553588 | 3.686532 | 5.21496 |
| 6 | −1.765052 | 5.062043 | 4.070216 |
| 1 | −1.7931 | 5.817957 | 4.850478 |
| 6 | −2.422121 | 5.282152 | 2.857744 |
| 1 | −2.960752 | 6.21105 | 2.689979 |
| 6 | −2.376716 | 4.313604 | 1.851799 |
| 1 | −2.867087 | 4.495468 | 0.898728 |
| 8 | −1.06711 | −0.094409 | −1.616483 |
| 6 | −2.432601 | −0.232276 | −1.371496 |
| 1 | −2.925103 | −0.51438 | −2.317029 |
| 1 | −2.895705 | 0.717907 | −1.064489 |

| | | | |
|---|---|---|---|
| 6 | −2.761233 | −1.295243 | −0.333361 |
| 6 | −2.094662 | −2.528111 | −0.360712 |
| 1 | −1.328978 | −2.696255 | −1.112062 |
| 6 | −2.386024 | −3.516433 | 0.579213 |
| 1 | −1.858082 | −4.466298 | 0.545734 |
| 6 | −3.352479 | −3.287795 | 1.565518 |
| 1 | −3.575333 | −4.055224 | 2.30186 |
| 6 | −4.025772 | −2.065166 | 1.598527 |
| 1 | −4.775001 | −1.875018 | 2.362685 |
| 6 | −3.72968 | −1.077285 | 0.653155 |
| 1 | −4.253331 | −0.124051 | 0.688449 |
| 17 | 0.079863 | 0.332105 | 6.408998 |
| 6 | 0.660501 | 2.610815 | −0.029276 |
| 6 | 1.609279 | 3.267232 | 1.002729 |
| 6 | 1.333068 | 4.7176 | 1.425505 |
| 6 | 1.659143 | 5.763172 | 0.345209 |
| 6 | 0.66942 | 5.80151 | −0.826536 |
| 6 | 0.503996 | 4.467573 | −1.548545 |
| 1 | 2.611693 | 3.211094 | 0.563142 |
| 1 | 0.297259 | 4.836479 | 1.752777 |
| 1 | 2.672444 | 5.57673 | −0.041782 |
| 1 | −0.319567 | 6.110866 | −0.462722 |
| 1 | 1.615846 | 2.61077 | 1.877783 |
| 1 | 1.958858 | 4.922451 | 2.304068 |
| 1 | 1.685632 | 6.75778 | 0.809744 |
| 1 | 0.992338 | 6.554869 | −1.558734 |
| 1 | −0.129292 | 4.586383 | −2.433538 |
| 1 | 1.465723 | 4.058484 | −1.88115 |
| 8 | 1.33922 | 1.763468 | −0.84695 |
| 8 | −0.169459 | 3.511121 | −0.7168 |

**Table S16.** Cartesian coordinate of **$C_{eq}$3b.**

| The number of atom coordinates (angstroms) | X | Y | Z |
|---|---|---|---|
| 13 | −0.067143 | 0.647997 | −0.435853 |
| 8 | 0.904862 | −0.355542 | −1.612634 |
| 8 | 0.705038 | 4.071986 | 0.324785 |
| 7 | 0.408491 | −0.559835 | 1.042579 |
| 6 | 1.312352 | −1.593674 | −1.535208 |
| 6 | 1.846348 | −2.201388 | −2.806267 |
| 1 | 2.690702 | −1.607655 | −3.166883 |
| 1 | 2.167197 | −3.239959 | −2.676313 |
| 1 | 1.069886 | −2.154797 | −3.578501 |

| | | | |
|---|---|---|---|
| 6 | 1.284825 | −2.344111 | −0.367509 |
| 1 | 1.616691 | −3.386806 | −0.406428 |
| 6 | 0.873382 | −1.823319 | 0.875229 |
| 6 | 0.971521 | −2.758549 | 2.063037 |
| 1 | 1.710899 | −2.384897 | 2.828428 |
| 1 | 0.011299 | −2.840492 | 2.53008 |
| 1 | 1.253222 | −3.754317 | 1.756112 |
| 6 | 0.158825 | −0.122501 | 2.393855 |
| 6 | −1.13206 | −0.229466 | 3.004892 |
| 1 | −1.949035 | −0.650411 | 2.443237 |
| 6 | −1.369902 | 0.193706 | 4.321013 |
| 1 | −2.371774 | 0.116348 | 4.79333 |
| 6 | −0.301554 | 0.71765 | 5.026043 |
| 6 | 0.993938 | 0.822938 | 4.440137 |
| 1 | 1.811314 | 1.243945 | 4.998146 |
| 6 | 1.216071 | 0.401088 | 3.122604 |
| 1 | 2.218427 | 0.476073 | 2.648275 |
| 6 | 1.712438 | 3.650721 | 1.275198 |
| 1 | 1.63013 | 2.562034 | 1.32861 |
| 1 | 2.696368 | 3.982307 | 0.883798 |
| 6 | 1.527751 | 4.267389 | 2.611254 |
| 6 | 2.42116 | 5.295123 | 3.136397 |
| 1 | 3.268665 | 5.663187 | 2.569331 |
| 6 | 2.23285 | 5.849502 | 4.383811 |
| 1 | 2.935791 | 6.646294 | 4.785234 |
| 6 | 1.143179 | 5.380506 | 5.11 |
| 1 | 0.995929 | 5.808891 | 6.081122 |
| 6 | 0.243149 | 4.354895 | 4.588053 |
| 1 | −0.599051 | 3.975927 | 5.155943 |
| 6 | 0.43734 | 3.800115 | 3.346417 |
| 1 | −0.246093 | 2.992527 | 2.937345 |
| 8 | −1.701051 | 0.377234 | −1.27042 |
| 6 | −2.115386 | −0.686362 | −2.113988 |
| 1 | −1.512391 | −0.824903 | −2.924597 |
| 1 | −3.150891 | −0.445006 | −2.605039 |
| 6 | −2.072353 | −2.00693 | −1.371593 |
| 6 | −1.65413 | −3.223135 | −1.967326 |
| 1 | −1.367336 | −3.228454 | −2.984059 |
| 6 | −1.581147 | −4.425229 | −1.266178 |
| 1 | −1.244136 | −5.35994 | −1.741774 |
| 6 | −1.922772 | −4.424964 | 0.048801 |
| 1 | −1.855154 | −5.357566 | 0.600654 |

| | | | |
|---|---|---|---|
| 6 | −2.356923 | −3.21555 | 0.64957 |
| 1 | −2.633788 | −3.20657 | 1.67094 |
| 6 | −2.436392 | −2.019442 | −0.059277 |
| 1 | −2.767769 | −1.073933 | 0.400531 |
| 17 | −0.596951 | 1.273004 | 6.682272 |
| 6 | 0.046101 | 3.160436 | −0.496551 |
| 6 | −0.789825 | 3.749931 | −1.490432 |
| 6 | −1.530087 | 4.88305 | −0.99136 |
| 6 | −2.751757 | 4.436281 | −0.190645 |
| 6 | −2.455306 | 3.724474 | 1.143335 |
| 6 | −2.155637 | 2.249844 | 1.040246 |
| 1 | −1.476396 | 2.919554 | −1.899812 |
| 1 | −0.824218 | 5.584675 | −0.395936 |
| 1 | −3.523771 | 3.783172 | −0.819884 |
| 1 | −1.636869 | 4.271072 | 1.712337 |
| 1 | −0.066062 | 4.11637 | −2.303128 |
| 1 | −1.854725 | 5.439234 | −1.87057 |
| 1 | −3.186973 | 5.330949 | 0.012786 |
| 1 | −3.341937 | 3.753698 | 1.744322 |
| 1 | −2.311572 | 1.794665 | 2.033539 |
| 1 | −2.809836 | 1.727351 | 0.352644 |
| 8 | 0.844516 | 2.126334 | −0.843918 |
| 8 | −0.788577 | 2.046323 | 0.630613 |

**Table S17.** Cartesian coordinate of **C$_{eq}$TS2b.**

| The number of atom coordinates (angstroms) | X | Y | Z |
|---|---|---|---|
| 13 | −0.048983 | 0.647997 | −0.435853 |
| 8 | 1.02039 | −0.355542 | −1.612634 |
| 8 | 0.84529 | 4.071986 | 0.324785 |
| 7 | 0.434372 | −0.559835 | 1.042579 |
| 6 | 1.357978 | −1.593674 | −1.535208 |
| 6 | 1.890519 | −2.201388 | −2.806267 |
| 1 | 2.737873 | −1.607655 | −3.166883 |
| 1 | 2.203458 | −3.239959 | −2.676313 |
| 1 | 1.11382 | −2.154797 | −3.578501 |
| 6 | 1.260913 | −2.344111 | −0.367509 |
| 1 | 1.547327 | −3.386806 | −0.406428 |
| 6 | 0.858454 | −1.823319 | 0.875229 |
| 6 | 0.920312 | −2.758549 | 2.063037 |
| 1 | 1.608112 | −2.384897 | 2.828428 |
| 1 | −0.066464 | −2.840492 | 2.53008 |
| 1 | 1.24412 | −3.754317 | 1.756112 |

| | | | |
|---|---|---|---|
| 6 | 0.2292 | −0.122501 | 2.393855 |
| 6 | −1.023488 | −0.229466 | 3.004892 |
| 1 | −1.850171 | −0.650411 | 2.443237 |
| 6 | −1.209801 | 0.193706 | 4.321013 |
| 1 | −2.183252 | 0.116348 | 4.79333 |
| 6 | −0.127376 | 0.71765 | 5.026043 |
| 6 | 1.133157 | 0.822938 | 4.440137 |
| 1 | 1.96172 | 1.243945 | 4.998146 |
| 6 | 1.303039 | 0.401088 | 3.122604 |
| 1 | 2.277101 | 0.476073 | 2.648275 |
| 6 | 1.86377 | 3.650721 | 1.275198 |
| 1 | 1.860945 | 2.562034 | 1.32861 |
| 1 | 2.829786 | 3.982307 | 0.883798 |
| 6 | 1.554509 | 4.267389 | 2.611254 |
| 6 | 2.342821 | 5.295123 | 3.136397 |
| 1 | 3.194896 | 5.663187 | 2.569331 |
| 6 | 2.042789 | 5.849502 | 4.383811 |
| 1 | 2.663233 | 6.646294 | 4.785234 |
| 6 | 0.946724 | 5.380506 | 5.11 |
| 1 | 0.713323 | 5.808891 | 6.081122 |
| 6 | 0.151881 | 4.354895 | 4.588053 |
| 1 | −0.69275 | 3.975927 | 5.155943 |
| 6 | 0.456105 | 3.800115 | 3.346417 |
| 1 | −0.140733 | 2.992527 | 2.937345 |
| 8 | −1.582798 | 0.377234 | −1.27042 |
| 6 | −1.925779 | −0.686362 | −2.113988 |
| 1 | −1.191558 | −0.824903 | −2.924597 |
| 1 | −2.8839 | −0.445006 | −2.605039 |
| 6 | −2.079953 | −2.00693 | −1.371593 |
| 6 | −1.730044 | −3.223135 | −1.967326 |
| 1 | −1.341564 | −3.228454 | −2.984059 |
| 6 | −1.850246 | −4.425229 | −1.266178 |
| 1 | −1.563394 | −5.35994 | −1.741774 |
| 6 | −2.32026 | −4.424964 | 0.048801 |
| 1 | −2.404383 | −5.357566 | 0.600654 |
| 6 | −2.683093 | −3.21555 | 0.64957 |
| 1 | −3.057087 | −3.20657 | 1.67094 |
| 6 | −2.56759 | −2.019442 | −0.059277 |
| 1 | −2.839119 | −1.073933 | 0.400531 |
| 17 | −0.360915 | 1.273004 | 6.682272 |
| 6 | 0.331144 | 3.160436 | −0.496551 |
| 6 | −0.646011 | 3.749931 | −1.490432 |

| | | | |
|---|---|---|---|
| 6 | −1.547933 | 4.88305 | −0.99136 |
| 6 | −2.778905 | 4.436281 | −0.190645 |
| 6 | −2.50066 | 3.724474 | 1.143335 |
| 6 | −2.102067 | 2.249844 | 1.040246 |
| 1 | −1.229463 | 2.919554 | −1.899812 |
| 1 | −0.952524 | 5.584675 | −0.395936 |
| 1 | −3.401386 | 3.783172 | −0.819884 |
| 1 | −1.739065 | 4.271072 | 1.712337 |
| 1 | −0.002716 | 4.11637 | −2.303128 |
| 1 | −1.89717 | 5.439234 | −1.87057 |
| 1 | −3.382698 | 5.330949 | 0.012786 |
| 1 | −3.420405 | 3.753698 | 1.744322 |
| 1 | −2.214907 | 1.794665 | 2.033539 |
| 1 | −2.780258 | 1.727351 | 0.352644 |
| 8 | 1.030039 | 2.126334 | −0.843918 |
| 8 | −0.756327 | 2.046323 | 0.630613 |

**Table S18.** Cartesian coordinate of **$C_{eq}$5b.**

| The number of atom coordinates (angstroms) | X | Y | Z |
|---|---|---|---|
| 13 | −0.125818 | 0.676639 | −0.345062 |
| 8 | 0.954605 | −0.299068 | −1.555787 |
| 8 | 1.062158 | 4.148168 | 0.224158 |
| 7 | 0.409588 | −0.581783 | 1.083316 |
| 6 | 1.305318 | −1.534766 | −1.520299 |
| 6 | 1.823681 | −2.099318 | −2.817874 |
| 1 | 2.658589 | −1.486199 | −3.175245 |
| 1 | 2.150136 | −3.137702 | −2.723551 |
| 1 | 1.03382 | −2.039931 | −3.575786 |
| 6 | 1.236384 | −2.320785 | −0.374334 |
| 1 | 1.537401 | −3.357804 | −0.44512 |
| 6 | 0.850522 | −1.833313 | 0.8873 |
| 6 | 0.954855 | −2.787161 | 2.057135 |
| 1 | 1.645768 | −2.407805 | 2.817061 |
| 1 | −0.02103 | −2.901287 | 2.540117 |
| 1 | 1.298644 | −3.769414 | 1.728529 |
| 6 | 0.225258 | −0.161657 | 2.442579 |
| 6 | −1.006035 | −0.312049 | 3.08672 |
| 1 | −1.833311 | −0.75874 | 2.545835 |
| 6 | −1.173932 | 0.113592 | 4.404258 |
| 1 | −2.13229 | 0.007112 | 4.901209 |
| 6 | −0.094979 | 0.686016 | 5.076255 |
| 6 | 1.146204 | 0.830824 | 4.45867 |

| | | | |
|---|---|---|---|
| 1 | 1.97195 | 1.288331 | 4.991749 |
| 6 | 1.298304 | 0.402608 | 3.141203 |
| 1 | 2.255475 | 0.513096 | 2.639875 |
| 6 | 2.034984 | 3.724591 | 1.238179 |
| 1 | 2.043745 | 2.634955 | 1.260623 |
| 1 | 3.008983 | 4.089345 | 0.903296 |
| 6 | 1.625215 | 4.310489 | 2.558752 |
| 6 | 2.348685 | 5.353773 | 3.144175 |
| 1 | 3.22004 | 5.759084 | 2.634502 |
| 6 | 1.958645 | 5.872974 | 4.381388 |
| 1 | 2.527834 | 6.681031 | 4.832918 |
| 6 | 0.839043 | 5.352815 | 5.034017 |
| 1 | 0.537074 | 5.753465 | 5.998051 |
| 6 | 0.109826 | 4.312882 | 4.448955 |
| 1 | −0.753239 | 3.896181 | 4.959994 |
| 6 | 0.501263 | 3.791964 | 3.216912 |
| 1 | −0.045154 | 2.978383 | 2.752161 |
| 8 | −1.617834 | 0.443179 | −1.271785 |
| 6 | −1.953025 | −0.625378 | −2.109549 |
| 1 | −1.208007 | −0.775701 | −2.908686 |
| 1 | −2.90313 | −0.385323 | −2.61731 |
| 6 | −2.125252 | −1.939979 | −1.359091 |
| 6 | −1.789729 | −3.163533 | −1.948588 |
| 1 | −1.398235 | −3.177984 | −2.96412 |
| 6 | −1.928407 | −4.361184 | −1.243654 |
| 1 | −1.652235 | −5.301454 | −1.714656 |
| 6 | −2.403481 | −4.349687 | 0.069551 |
| 1 | −2.502932 | −5.279059 | 0.62437 |
| 6 | −2.751366 | −3.13319 | 0.664412 |
| 1 | −3.129394 | −3.115488 | 1.684235 |
| 6 | −2.616429 | −1.940804 | −0.047739 |
| 1 | −2.875909 | −0.990923 | 0.410092 |
| 17 | −0.308985 | 1.257385 | 6.730976 |
| 6 | 0.572782 | 3.25727 | −0.603607 |
| 6 | −0.485003 | 3.783062 | −1.534594 |
| 6 | −1.489259 | 4.797217 | −0.96227 |
| 6 | −2.738966 | 4.205131 | −0.284838 |
| 6 | −2.571243 | 3.60299 | 1.123619 |
| 6 | −2.130624 | 2.137391 | 1.177888 |
| 1 | −0.994884 | 2.916264 | −1.963974 |
| 1 | −0.974128 | 5.475171 | −0.272079 |
| 1 | −3.187 | 3.452711 | −0.949512 |

| | | | |
|---|---|---|---|
| 1 | −1.870965 | 4.212886 | 1.709595 |
| 1 | 0.086702 | 4.251516 | −2.350521 |
| 1 | −1.829153 | 5.414658 | −1.802998 |
| 1 | −3.469686 | 5.022392 | −0.216571 |
| 1 | −3.540881 | 3.66305 | 1.639411 |
| 1 | −2.233976 | 1.791679 | 2.220076 |
| 1 | −2.81863 | 1.535418 | 0.564482 |
| 8 | 1.105682 | 2.126917 | −0.768213 |
| 8 | −0.802699 | 1.966971 | 0.753621 |

**Table S19.** Cartesian coordinate of **$C_{ax}$1b.**

| The number of atom coordinates (angstroms) | X | Y | Z |
|---|---|---|---|
| 13 | 0.381762 | −0.04108 | −1.138953 |
| 8 | 0.709677 | −1.615977 | −2.106694 |
| 8 | 1.686894 | 0.822899 | −1.938369 |
| 7 | 0.844831 | −0.947067 | 0.56793 |
| 6 | 0.911043 | −2.817027 | −1.70986 |
| 6 | 0.957238 | −3.85972 | −2.796588 |
| 1 | 1.150026 | −4.863209 | −2.409799 |
| 1 | 1.733007 | −3.593421 | −3.523153 |
| 1 | −0.000031 | −3.858825 | −3.329672 |
| 6 | 1.091329 | −3.168496 | −0.373791 |
| 1 | 1.268163 | −4.21147 | −0.144453 |
| 6 | 1.079674 | −2.261608 | 0.701217 |
| 6 | 1.360284 | −2.843664 | 2.071678 |
| 1 | 2.089026 | −2.239424 | 2.619979 |
| 1 | 0.444981 | −2.8689 | 2.672598 |
| 1 | 1.73854 | −3.864012 | 1.987056 |
| 6 | 0.88276 | −0.145716 | 1.755368 |
| 6 | −0.170164 | −0.173128 | 2.676312 |
| 6 | −0.15872 | 0.658006 | 3.795216 |
| 1 | −0.973539 | 0.63357 | 4.511038 |
| 6 | 0.90872 | 1.536181 | 3.977766 |
| 6 | 1.971981 | 1.577346 | 3.075563 |
| 1 | 2.795733 | 2.265221 | 3.232659 |
| 6 | 1.954613 | 0.728895 | 1.969897 |
| 6 | 2.245578 | 2.081063 | −1.74486 |
| 1 | 2.484498 | 2.265751 | −0.68207 |
| 1 | 3.210115 | 2.125061 | −2.280027 |
| 6 | 1.400966 | 3.259806 | −2.218378 |
| 6 | 0.18162 | 3.085078 | −2.878647 |
| 1 | −0.18308 | 2.080749 | −3.064358 |

| | | | |
|---|---|---|---|
| 6 | −0.583727 | 4.191195 | −3.259604 |
| 1 | −1.533978 | 4.036268 | −3.765477 |
| 6 | −0.139282 | 5.48764 | −2.992131 |
| 1 | −0.736885 | 6.346346 | −3.287605 |
| 6 | 1.084966 | 5.671533 | −2.341763 |
| 1 | 1.444527 | 6.675502 | −2.128537 |
| 6 | 1.845407 | 4.564918 | −1.961462 |
| 1 | 2.793704 | 4.713742 | −1.446368 |
| 8 | −1.305317 | 0.11683 | −1.655664 |
| 6 | −2.096847 | −0.908187 | −2.185425 |
| 1 | −1.650595 | −1.355339 | −3.088272 |
| 1 | −3.060607 | −0.471675 | −2.501673 |
| 6 | −2.38489 | −2.026825 | −1.191016 |
| 6 | −2.538011 | −3.351971 | −1.613767 |
| 1 | −2.446247 | −3.588829 | −2.672155 |
| 6 | −2.792346 | −4.374136 | −0.695897 |
| 1 | −2.896987 | −5.399227 | −1.043012 |
| 6 | −2.896821 | −4.082168 | 0.665679 |
| 1 | −3.087062 | −4.876136 | 1.383311 |
| 6 | −2.754426 | −2.759956 | 1.096512 |
| 1 | −2.843174 | −2.523434 | 2.155264 |
| 6 | −2.501705 | −1.74224 | 0.175707 |
| 1 | −2.367613 | −0.716838 | 0.503089 |
| 6 | −0.961093 | 2.29643 | 0.388593 |
| 6 | −0.928382 | 3.800135 | 0.47827 |
| 6 | −0.953278 | 4.322769 | 1.933291 |
| 6 | −2.345334 | 4.322886 | 2.575538 |
| 6 | −3.009425 | 2.942602 | 2.663611 |
| 6 | −3.212814 | 2.249777 | 1.319756 |
| 1 | −1.772197 | 4.213302 | −0.089008 |
| 1 | −0.253035 | 3.736835 | 2.541565 |
| 1 | −3.000187 | 4.993902 | 2.000914 |
| 1 | −2.424364 | 2.273825 | 3.306019 |
| 1 | −0.017269 | 4.112276 | −0.032282 |
| 1 | −0.569641 | 5.348975 | 1.918917 |
| 1 | −2.273334 | 4.746361 | 3.584469 |
| 1 | −3.997647 | 3.047143 | 3.129147 |
| 1 | −3.906553 | 1.412519 | 1.409765 |
| 1 | −3.598048 | 2.932823 | 0.554806 |
| 8 | 0.007672 | 1.673801 | −0.042879 |
| 8 | −2.007005 | 1.608997 | 0.822769 |
| 1 | 2.762978 | 0.756686 | 1.246176 |

| | | | |
|---|---|---|---|
| 1 | −1.008016 | −0.838249 | 2.500631 |
| 17 | 0.901513 | 2.628104 | 5.363541 |

**Table S20.** Cartesian coordinate of **C$_{ax}$TS1b.**

| The number of atom coordinates (angstroms) | X | Y | Z |
|---|---|---|---|
| 13 | 0.643663 | 0.185684 | −0.588301 |
| 8 | 0.526888 | −1.339918 | −1.654383 |
| 8 | 1.987725 | 0.930669 | −1.430704 |
| 7 | 1.228538 | −0.867582 | 0.958711 |
| 6 | 0.590279 | −2.584316 | −1.326532 |
| 6 | 0.231843 | −3.553982 | −2.420697 |
| 1 | 0.444154 | −4.590747 | −2.1489 |
| 1 | 0.775715 | −3.293711 | −3.334815 |
| 1 | −0.839751 | −3.456293 | −2.632999 |
| 6 | 0.940557 | −3.036862 | −0.058297 |
| 1 | 0.969529 | −4.106401 | 0.106528 |
| 6 | 1.289351 | −2.199828 | 1.022575 |
| 6 | 1.753685 | −2.867228 | 2.298676 |
| 1 | 2.799149 | −2.614529 | 2.506234 |
| 1 | 1.169917 | −2.516458 | 3.156005 |
| 1 | 1.665481 | −3.952943 | 2.228538 |
| 6 | 1.731988 | −0.108498 | 2.068704 |
| 6 | 0.876 | 0.33439 | 3.081982 |
| 6 | 1.370763 | 1.095904 | 4.138 |
| 1 | 0.710514 | 1.444565 | 4.924869 |
| 6 | 2.727073 | 1.422593 | 4.164982 |
| 6 | 3.591675 | 1.008784 | 3.154147 |
| 1 | 4.640761 | 1.282579 | 3.183967 |
| 6 | 3.084765 | 0.241784 | 2.103808 |
| 6 | 2.465202 | 2.228238 | −1.179159 |
| 1 | 2.575169 | 2.425904 | −0.101381 |
| 1 | 3.474755 | 2.311139 | −1.615837 |
| 6 | 1.592007 | 3.318261 | −1.779345 |
| 6 | 0.932151 | 3.10555 | −2.99602 |
| 1 | 1.045494 | 2.144558 | −3.488932 |
| 6 | 0.12017 | 4.097384 | −3.549252 |
| 1 | −0.391433 | 3.914148 | −4.491192 |
| 6 | −0.041078 | 5.321584 | −2.893425 |
| 1 | −0.677547 | 6.092541 | −3.320192 |
| 6 | 0.618412 | 5.544366 | −1.681737 |
| 1 | 0.492532 | 6.489047 | −1.158246 |
| 6 | 1.426294 | 4.546988 | −1.130485 |

| | | | |
|---|---|---|---|
| 1 | 1.915211 | 4.714814 | −0.173195 |
| 8 | −1.175493 | 0.513765 | −0.948138 |
| 6 | −2.220444 | −0.110489 | −1.664588 |
| 1 | −1.894742 | −0.242649 | −2.703541 |
| 1 | −3.072767 | 0.584609 | −1.68666 |
| 6 | −2.660445 | −1.447762 | −1.097064 |
| 6 | −3.357855 | −2.341648 | −1.920835 |
| 1 | −3.560046 | −2.0721 | −2.955833 |
| 6 | −3.783812 | −3.577359 | −1.432059 |
| 1 | −4.31881 | −4.261661 | −2.085458 |
| 6 | −3.510903 | −3.93834 | −0.109468 |
| 1 | −3.833137 | −4.903883 | 0.271044 |
| 6 | −2.815868 | −3.052379 | 0.714681 |
| 1 | −2.591123 | −3.327254 | 1.742156 |
| 6 | −2.395932 | −1.812998 | 0.2261 |
| 1 | −1.851259 | −1.128737 | 0.866301 |
| 6 | −1.180176 | 1.733179 | 0.333753 |
| 6 | −1.757877 | 2.952265 | −0.358653 |
| 6 | −2.005498 | 4.109802 | 0.628354 |
| 6 | −3.358273 | 4.017329 | 1.350854 |
| 6 | −3.539779 | 2.776596 | 2.23809 |
| 6 | −3.29636 | 1.444977 | 1.530163 |
| 1 | −2.690058 | 2.697638 | −0.875386 |
| 1 | −1.1799 | 4.151994 | 1.3503 |
| 1 | −4.156203 | 4.028104 | 0.593559 |
| 1 | −2.859082 | 2.819436 | 3.098307 |
| 1 | −1.033017 | 3.237184 | −1.118438 |
| 1 | −1.967619 | 5.047755 | 0.063024 |
| 1 | −3.505329 | 4.91444 | 1.965409 |
| 1 | −4.56134 | 2.767126 | 2.640767 |
| 1 | −3.646544 | 0.609484 | 2.141111 |
| 1 | −3.814723 | 1.388852 | 0.566327 |
| 8 | 0.098086 | 1.654618 | 0.511881 |
| 8 | −1.885925 | 1.188512 | 1.366959 |
| 1 | 3.735934 | −0.081397 | 1.297323 |
| 1 | −0.182831 | 0.11309 | 3.014073 |
| 17 | 3.355699 | 2.391512 | 5.499833 |

**Table S21.** Cartesian coordinate of **$C_{ax}$2b.**

| The number of atom coordinates (angstroms) | X | Y | Z |
|---|---|---|---|
| 13 | 0.614776 | 0.282638 | −0.452084 |
| 8 | 0.380849 | −1.157396 | −1.59108 |

| | | | |
|---|---|---|---|
| 8 | 2.000405 | 0.957341 | −1.292139 |
| 7 | 1.047923 | −0.875291 | 1.057306 |
| 6 | 0.743525 | −2.384026 | −1.433029 |
| 6 | 0.560366 | −3.262439 | −2.640301 |
| 1 | 0.923878 | −4.279843 | −2.477933 |
| 1 | 1.083699 | −2.819735 | −3.495 |
| 1 | −0.506347 | −3.296747 | −2.88768 |
| 6 | 1.241586 | −2.899215 | −0.239277 |
| 1 | 1.517975 | −3.945768 | −0.218396 |
| 6 | 1.340334 | −2.176176 | 0.967542 |
| 6 | 1.785557 | −2.933564 | 2.199174 |
| 1 | 2.557246 | −2.377808 | 2.740949 |
| 1 | 0.948293 | −3.070725 | 2.892025 |
| 1 | 2.174226 | −3.918168 | 1.932013 |
| 6 | 1.063865 | −0.241622 | 2.343342 |
| 6 | 0.008748 | −0.434495 | 3.240399 |
| 6 | −0.019721 | 0.241278 | 4.45833 |
| 1 | −0.840895 | 0.097511 | 5.152363 |
| 6 | 1.014753 | 1.123816 | 4.767014 |
| 6 | 2.074799 | 1.331768 | 3.885776 |
| 1 | 2.868139 | 2.026321 | 4.139725 |
| 6 | 2.093708 | 0.643576 | 2.673323 |
| 6 | 2.277129 | 2.328128 | −1.397906 |
| 1 | 2.315396 | 2.817254 | −0.411394 |
| 1 | 3.278721 | 2.445793 | −1.844684 |
| 6 | 1.273599 | 3.085648 | −2.258326 |
| 6 | 0.521435 | 2.422316 | −3.235052 |
| 1 | 0.667228 | 1.355462 | −3.37413 |
| 6 | −0.415477 | 3.115021 | −4.005352 |
| 1 | −0.997121 | 2.582831 | −4.754542 |
| 6 | −0.608764 | 4.485566 | −3.814992 |
| 1 | −1.342627 | 5.024104 | −4.409089 |
| 6 | 0.148711 | 5.158812 | −2.852336 |
| 1 | 0.003402 | 6.224257 | −2.691605 |
| 6 | 1.08041 | 4.460986 | −2.082026 |
| 1 | 1.649075 | 4.986338 | −1.317244 |
| 8 | −1.31722 | 0.731322 | −0.805528 |
| 6 | −2.557969 | 0.204071 | −1.315154 |
| 1 | −2.609147 | 0.455978 | −2.37856 |
| 1 | −3.360706 | 0.729401 | −0.789845 |
| 6 | −2.670086 | −1.286127 | −1.100572 |
| 6 | −3.056918 | −2.12332 | −2.150659 |

| | | | |
|---|---|---|---|
| 1 | −3.256426 | −1.699007 | −3.13209 |
| 6 | −3.174951 | −3.502178 | −1.952435 |
| 1 | −3.476106 | −4.14265 | −2.777266 |
| 6 | −2.888397 | −4.054973 | −0.703351 |
| 1 | −2.964193 | −5.128213 | −0.550816 |
| 6 | −2.500813 | −3.220485 | 0.349708 |
| 1 | −2.274529 | −3.64532 | 1.324434 |
| 6 | −2.403882 | −1.843929 | 0.156656 |
| 1 | −2.108247 | −1.186033 | 0.966673 |
| 6 | −1.268754 | 1.754429 | 0.28077 |
| 6 | −1.778189 | 3.104912 | −0.235497 |
| 6 | −1.232073 | 4.28706 | 0.575837 |
| 6 | −1.779451 | 4.395734 | 2.003901 |
| 6 | −1.636551 | 3.11582 | 2.84308 |
| 6 | −2.541127 | 1.9687 | 2.382523 |
| 1 | −2.875842 | 3.105131 | −0.249622 |
| 1 | −0.142313 | 4.189421 | 0.60302 |
| 1 | −2.845006 | 4.669462 | 1.96665 |
| 1 | −0.598425 | 2.768559 | 2.859394 |
| 1 | −1.428817 | 3.193579 | −1.267083 |
| 1 | −1.453605 | 5.212545 | 0.030333 |
| 1 | −1.268527 | 5.221094 | 2.516711 |
| 1 | −1.916231 | 3.341434 | 3.880735 |
| 1 | −2.663542 | 1.246739 | 3.195522 |
| 1 | −3.539057 | 2.362226 | 2.133202 |
| 8 | 0.045601 | 1.723308 | 0.545853 |
| 8 | −2.067165 | 1.169038 | 1.29049 |
| 1 | 2.898497 | 0.807852 | 1.963315 |
| 1 | −0.805083 | −1.098616 | 2.969418 |
| 17 | 0.970595 | 2.005085 | 6.294513 |

**Table S22.** Cartesian coordinate of **$C_{ax}$3b.**

| The number of atom coordinates (angstroms) | X | Y | Z |
|---|---|---|---|
| 13 | 0.150501 | 0.802166 | 0.632281 |
| 8 | −0.518963 | −0.479016 | −0.463561 |
| 8 | 1.376428 | 1.795562 | −0.11232 |
| 7 | 1.273233 | −0.481779 | 1.652296 |
| 6 | −0.389624 | −1.778029 | −0.384494 |
| 6 | −1.214849 | −2.535489 | −1.388567 |
| 1 | −1.079069 | −3.616843 | −1.314248 |
| 1 | −0.949902 | −2.206367 | −2.400202 |
| 1 | −2.274545 | −2.294454 | −1.24131 |

| | | | |
|---|---|---|---|
| 6 | 0.426002 | −2.426952 | 0.517217 |
| 1 | 0.455357 | −3.508941 | 0.478254 |
| 6 | 1.275534 | −1.789069 | 1.473434 |
| 6 | 2.199893 | −2.69028 | 2.260502 |
| 1 | 3.168805 | −2.759626 | 1.751722 |
| 1 | 2.381787 | −2.306407 | 3.266754 |
| 1 | 1.784671 | −3.698745 | 2.327907 |
| 6 | 2.212313 | 0.134316 | 2.542888 |
| 6 | 1.748103 | 0.821294 | 3.671068 |
| 6 | 2.646772 | 1.479277 | 4.510781 |
| 1 | 2.292103 | 2.009957 | 5.387724 |
| 6 | 4.007234 | 1.453038 | 4.208462 |
| 6 | 4.484075 | 0.784806 | 3.081569 |
| 1 | 5.543218 | 0.786772 | 2.84786 |
| 6 | 3.580827 | 0.12802 | 2.249603 |
| 6 | 1.997896 | 1.584344 | −1.358951 |
| 1 | 1.296659 | 1.149897 | −2.090564 |
| 1 | 2.312347 | 2.559155 | −1.761737 |
| 6 | 3.209489 | 0.677496 | −1.237132 |
| 6 | 4.47515 | 1.204865 | −0.956116 |
| 1 | 4.595445 | 2.282721 | −0.875016 |
| 6 | 5.575423 | 0.365375 | −0.767762 |
| 1 | 6.551775 | 0.791662 | −0.551966 |
| 6 | 5.420217 | −1.021307 | −0.851695 |
| 1 | 6.274231 | −1.676921 | −0.703419 |
| 6 | 4.16009 | −1.559676 | −1.131407 |
| 1 | 4.029455 | −2.636794 | −1.201717 |
| 6 | 3.066859 | −0.71298 | −1.325623 |
| 1 | 2.088752 | −1.136786 | −1.536996 |
| 8 | −1.831182 | 3.444544 | 1.989177 |
| 6 | −0.621868 | 4.206242 | 1.811889 |
| 1 | 0.255321 | 3.559525 | 1.895033 |
| 1 | −0.608918 | 4.901708 | 2.657973 |
| 6 | −0.624896 | 4.965849 | 0.503335 |
| 6 | 0.418707 | 4.826281 | −0.416911 |
| 1 | 1.225788 | 4.134937 | −0.197825 |
| 6 | 0.392123 | 5.528022 | −1.625326 |
| 1 | 1.206461 | 5.407856 | −2.335749 |
| 6 | −0.679415 | 6.371436 | −1.926422 |
| 1 | −0.70085 | 6.914103 | −2.868088 |
| 6 | −1.726863 | 6.512995 | −1.01068 |
| 1 | −2.564708 | 7.16747 | −1.237933 |

| | | | |
|---|---|---|---|
| 6 | −1.695839 | 5.815553 | 0.196856 |
| 1 | −2.514226 | 5.914 | 0.906007 |
| 6 | −1.80986 | 2.120402 | 1.54109 |
| 6 | −3.208265 | 1.558202 | 1.823869 |
| 6 | −3.608389 | 0.350925 | 0.961397 |
| 6 | −4.173617 | 0.72661 | −0.422589 |
| 6 | −3.160754 | 1.29431 | −1.429028 |
| 6 | −2.357516 | 2.480868 | −0.909997 |
| 1 | −3.940472 | 2.364546 | 1.703272 |
| 1 | −2.754218 | −0.326634 | 0.84945 |
| 1 | −4.985934 | 1.454528 | −0.278921 |
| 1 | −2.446057 | 0.515473 | −1.715792 |
| 1 | −3.201562 | 1.29068 | 2.885156 |
| 1 | −4.381448 | −0.209452 | 1.50132 |
| 1 | −4.63754 | −0.163478 | −0.868312 |
| 1 | −3.689701 | 1.606974 | −2.339798 |
| 1 | −1.730526 | 2.92151 | −1.689946 |
| 1 | −2.989381 | 3.276989 | −0.502733 |
| 8 | −0.793742 | 1.362646 | 2.056282 |
| 8 | −1.448374 | 2.062735 | 0.120957 |
| 1 | 3.93622 | −0.374554 | 1.358985 |
| 1 | 0.684117 | 0.853515 | 3.873146 |
| 17 | 5.146205 | 2.289139 | 5.265439 |

**Table S23.** Cartesian coordinate of **$C_{ax}$TS2b.**

| The number of atom coordinates (angstroms) | X | Y | Z |
|---|---|---|---|
| 13 | 0.458092 | 1.311194 | 0.221597 |
| 8 | 0.08791 | 0.321218 | −1.331168 |
| 8 | 1.885284 | 2.17514 | −0.335608 |
| 7 | 0.867446 | −0.348863 | 1.217994 |
| 6 | −0.091477 | −0.943196 | −1.494477 |
| 6 | −0.58922 | −1.349568 | −2.85709 |
| 1 | −0.705608 | −2.431084 | −2.958845 |
| 1 | 0.108853 | −0.986804 | −3.620255 |
| 1 | −1.55298 | −0.86322 | −3.047847 |
| 6 | 0.175304 | −1.890572 | −0.513145 |
| 1 | 0.032362 | −2.934355 | −0.762713 |
| 6 | 0.715373 | −1.592323 | 0.761819 |
| 6 | 1.172621 | −2.765355 | 1.601152 |
| 1 | 2.2482 | −2.680645 | 1.793573 |
| 1 | 0.676686 | −2.782594 | 2.575917 |
| 1 | 0.981006 | −3.711002 | 1.091014 |

| | | | |
|---|---|---|---|
| 6 | 1.493413 | −0.174475 | 2.50359 |
| 6 | 0.756346 | −0.300636 | 3.685695 |
| 6 | 1.363357 | −0.10306 | 4.924185 |
| 1 | 0.792932 | −0.2005 | 5.841609 |
| 6 | 2.717323 | 0.22863 | 4.969945 |
| 6 | 3.465688 | 0.365322 | 3.803267 |
| 1 | 4.517157 | 0.626917 | 3.851507 |
| 6 | 2.847161 | 0.16358 | 2.569474 |
| 6 | 2.743251 | 1.796443 | −1.389388 |
| 1 | 2.185326 | 1.353036 | −2.22791 |
| 1 | 3.243102 | 2.699355 | −1.772721 |
| 6 | 3.791838 | 0.81012 | −0.908345 |
| 6 | 4.943719 | 1.26193 | −0.251433 |
| 1 | 5.115658 | 2.331859 | −0.158968 |
| 6 | 5.85397 | 0.358556 | 0.301594 |
| 1 | 6.741624 | 0.725882 | 0.810395 |
| 6 | 5.620556 | −1.016668 | 0.206416 |
| 1 | 6.324139 | −1.721446 | 0.641587 |
| 6 | 4.479258 | −1.478951 | −0.453791 |
| 1 | 4.290824 | −2.546738 | −0.535007 |
| 6 | 3.575766 | −0.569836 | −1.008743 |
| 1 | 2.682142 | −0.935583 | −1.506518 |
| 8 | −1.015028 | 4.17275 | 1.650787 |
| 6 | −0.035699 | 4.852639 | 0.814098 |
| 1 | 0.887516 | 4.273049 | 0.776153 |
| 1 | 0.150483 | 5.798347 | 1.331095 |
| 6 | −0.615488 | 5.079509 | −0.561286 |
| 6 | 0.035083 | 4.591151 | −1.69872 |
| 1 | 0.950856 | 4.02132 | −1.577693 |
| 6 | −0.512864 | 4.797836 | −2.966743 |
| 1 | −0.001462 | 4.409502 | −3.843785 |
| 6 | −1.718332 | 5.48837 | −3.106971 |
| 1 | −2.146777 | 5.645369 | −4.093545 |
| 6 | −2.374536 | 5.97572 | −1.972535 |
| 1 | −3.31333 | 6.514043 | −2.074973 |
| 6 | −1.822503 | 5.774523 | −0.707304 |
| 1 | −2.334702 | 6.145959 | 0.176905 |
| 6 | −0.878551 | 2.856842 | 1.854774 |
| 6 | −2.01596 | 2.333449 | 2.710542 |
| 6 | −2.531698 | 0.915071 | 2.407889 |
| 6 | −3.628308 | 0.807716 | 1.323728 |
| 6 | −3.152577 | 0.701662 | −0.133649 |

| | | | |
|---|---|---|---|
| 6 | −2.369829 | 1.911691 | −0.634186 |
| 1 | −2.84005 | 3.051938 | 2.671997 |
| 1 | −1.691861 | 0.259584 | 2.154971 |
| 1 | −4.314557 | 1.661783 | 1.421674 |
| 1 | −2.515942 | −0.187908 | −0.240664 |
| 1 | −1.611723 | 2.360981 | 3.731398 |
| 1 | −2.950747 | 0.527992 | 3.34521 |
| 1 | −4.227893 | −0.085685 | 1.542245 |
| 1 | −4.025608 | 0.548118 | −0.783887 |
| 1 | −2.10261 | 1.788567 | −1.691646 |
| 1 | −2.970323 | 2.829212 | −0.551451 |
| 8 | 0.27606 | 2.302189 | 1.870563 |
| 8 | −1.193544 | 2.093775 | 0.118908 |
| 1 | 3.414988 | 0.274349 | 1.655565 |
| 1 | −0.297455 | −0.552761 | 3.637138 |
| 17 | 3.493082 | 0.480358 | 6.535134 |

**Table S24.** Cartesian coordinate of **$C_{ax}$4b.**

| The number of atom coordinates (angstroms) | X | Y | Z |
|---|---|---|---|
| 13 | 0.41324 | 1.10878 | −0.067892 |
| 8 | 0.126049 | 0.105269 | −1.632844 |
| 8 | 1.793612 | 2.045882 | −0.65013 |
| 7 | 0.895452 | −0.518654 | 0.95255 |
| 6 | 0.144114 | −1.162955 | −1.832742 |
| 6 | −0.231506 | −1.602785 | −3.224252 |
| 1 | −0.213995 | −2.688717 | −3.34341 |
| 1 | 0.459342 | −1.149749 | −3.944915 |
| 1 | −1.233355 | −1.228052 | −3.462606 |
| 6 | 0.508548 | −2.088616 | −0.86001 |
| 1 | 0.525475 | −3.134964 | −1.137469 |
| 6 | 0.914159 | −1.759469 | 0.452981 |
| 6 | 1.43415 | −2.900711 | 1.301961 |
| 1 | 2.466359 | −2.693517 | 1.605497 |
| 1 | 0.850669 | −3.020311 | 2.219464 |
| 1 | 1.412878 | −3.84077 | 0.747868 |
| 6 | 1.357527 | −0.352904 | 2.304899 |
| 6 | 0.495298 | −0.585666 | 3.380257 |
| 6 | 0.926213 | −0.38626 | 4.691453 |
| 1 | 0.258441 | −0.565183 | 5.527505 |
| 6 | 2.231941 | 0.050352 | 4.914226 |
| 6 | 3.106952 | 0.281522 | 3.854073 |
| 1 | 4.120253 | 0.619559 | 4.042865 |

| | | | |
|---|---|---|---|
| 6 | 2.66397 | 0.07819 | 2.548073 |
| 6 | 2.756696 | 1.62791 | −1.588981 |
| 1 | 2.305936 | 1.039698 | −2.402839 |
| 1 | 3.206416 | 2.522566 | −2.047058 |
| 6 | 3.84581 | 0.806097 | −0.92255 |
| 6 | 4.833841 | 1.434503 | −0.152392 |
| 1 | 4.846925 | 2.520268 | −0.092041 |
| 6 | 5.781876 | 0.684943 | 0.546386 |
| 1 | 6.541826 | 1.187941 | 1.139087 |
| 6 | 5.750919 | −0.712258 | 0.488075 |
| 1 | 6.484685 | −1.298129 | 1.035286 |
| 6 | 4.772706 | −1.34921 | −0.278867 |
| 1 | 4.741847 | −2.434789 | −0.331183 |
| 6 | 3.830136 | −0.592239 | −0.979963 |
| 1 | 3.061876 | −1.094277 | −1.562381 |
| 8 | −0.555793 | 4.178198 | 1.721276 |
| 6 | 0.206069 | 4.702497 | 0.582755 |
| 1 | 1.040197 | 4.037098 | 0.358545 |
| 1 | 0.582388 | 5.663886 | 0.943504 |
| 6 | −0.715221 | 4.872251 | −0.598096 |
| 6 | −0.397738 | 4.291075 | −1.829363 |
| 1 | 0.496718 | 3.681461 | −1.909995 |
| 6 | −1.247724 | 4.458304 | −2.92436 |
| 1 | −0.995799 | 3.997053 | −3.875651 |
| 6 | −2.425102 | 5.197915 | −2.794314 |
| 1 | −3.089523 | 5.321343 | −3.645606 |
| 6 | −2.750032 | 5.77439 | −1.562892 |
| 1 | −3.666485 | 6.348443 | −1.453906 |
| 6 | −1.894985 | 5.616207 | −0.472094 |
| 1 | −2.147877 | 6.059793 | 0.487714 |
| 6 | −0.369182 | 2.933259 | 2.120308 |
| 6 | −1.342571 | 2.5132 | 3.19205 |
| 6 | −2.410326 | 1.534256 | 2.650102 |
| 6 | −3.414088 | 2.140085 | 1.632043 |
| 6 | −3.549566 | 1.307348 | 0.33941 |
| 6 | −2.427915 | 1.624034 | −0.66525 |
| 1 | −1.816457 | 3.400466 | 3.621195 |
| 1 | −1.889456 | 0.690571 | 2.193362 |
| 1 | −3.106326 | 3.157343 | 1.360701 |
| 1 | −3.535887 | 0.238369 | 0.597376 |
| 1 | −0.756937 | 2.01109 | 3.969364 |
| 1 | −2.948544 | 1.138413 | 3.52051 |

| | | | |
|---|---|---|---|
| 1 | −4.395859 | 2.234313 | 2.112205 |
| 1 | −4.518663 | 1.499955 | −0.141579 |
| 1 | −2.392263 | 0.850695 | −1.450335 |
| 1 | −2.65887 | 2.57866 | −1.164677 |
| 8 | 0.537688 | 2.200675 | 1.703964 |
| 8 | −1.210893 | 1.754042 | 0.003482 |
| 1 | 3.327464 | 0.265868 | 1.713608 |
| 1 | −0.519789 | −0.917718 | 3.186963 |
| 17 | 2.784578 | 0.312586 | 6.569793 |

**Table S25.** Cartesian coordinate of **C$_{eq}$1c.**

| The number of atom coordinates (angstroms) | X | Y | Z |
|---|---|---|---|
| 13 | −0.098276 | 0.30334 | 0.104325 |
| 8 | 0.515018 | −0.832352 | −1.125019 |
| 8 | −0.219085 | 1.670679 | 1.193753 |
| 7 | 0.332755 | −1.091682 | 1.627065 |
| 6 | 0.840982 | −2.073297 | −0.971284 |
| 6 | 1.043021 | −2.700503 | −2.241224 |
| 1 | 0.066206 | −2.781398 | −2.73288 |
| 1 | 1.670942 | −2.050452 | −2.920587 |
| 1 | 1.492111 | −3.695132 | −2.062762 |
| 6 | 0.987689 | −2.825869 | 0.27495 |
| 1 | 1.293901 | −3.859607 | 0.305137 |
| 6 | 0.761835 | −2.332417 | 1.508149 |
| 6 | 1.017453 | −3.284061 | 2.739672 |
| 1 | 0.087169 | −3.502187 | 3.022322 |
| 1 | 1.442478 | −4.224469 | 2.529878 |
| 1 | 1.69977 | −2.839836 | 3.599732 |
| 6 | 0.148041 | −0.704867 | 2.966219 |
| 6 | −1.083418 | −0.961236 | 3.60317 |
| 6 | −1.267255 | −0.480041 | 4.895164 |
| 1 | −2.216423 | −0.650023 | 5.393386 |
| 6 | −0.259255 | 0.212571 | 5.555651 |
| 6 | 0.96535 | 0.419278 | 4.917837 |
| 1 | 1.757664 | 0.944646 | 5.440862 |
| 6 | 1.192546 | −0.031618 | 3.61085 |
| 6 | −1.38142 | 2.175446 | 1.665741 |
| 1 | −2.232462 | 2.167408 | 0.832699 |
| 1 | −1.671106 | 1.55749 | 2.35459 |
| 6 | −1.140545 | 3.59254 | 2.389059 |
| 6 | −0.13994 | 3.839703 | 3.554047 |
| 1 | 0.413676 | 2.99771 | 3.938541 |

| | | | |
|---|---|---|---|
| 6 | 0.146143 | 5.13926 | 4.206935 |
| 1 | 0.928891 | 5.313598 | 5.105492 |
| 6 | −0.574837 | 6.219766 | 3.710547 |
| 1 | −0.352735 | 7.23369 | 4.217132 |
| 6 | −1.589748 | 5.984327 | 2.565769 |
| 1 | −2.162489 | 6.815337 | 2.177367 |
| 6 | −1.868046 | 4.677222 | 1.911869 |
| 1 | −2.654383 | 4.497608 | 1.011497 |
| 8 | −1.745701 | 0.379696 | −0.240454 |
| 6 | −2.195316 | 0.078326 | −1.49718 |
| 1 | −1.516143 | 0.472369 | −2.301776 |
| 1 | −3.16214 | 0.588731 | −1.499727 |
| 6 | −2.408489 | −1.403395 | −1.869806 |
| 6 | −2.540961 | −1.854871 | −3.212338 |
| 1 | −2.471437 | −1.140541 | −3.993381 |
| 6 | −2.74321 | −3.207989 | −3.560407 |
| 1 | −2.836652 | −3.54074 | −4.608355 |
| 6 | −2.810571 | −4.13584 | −2.564838 |
| 1 | −2.957341 | −5.191419 | −2.833302 |
| 6 | −2.684724 | −3.692757 | −1.223334 |
| 1 | −2.731681 | −4.40414 | −0.440161 |
| 6 | −2.491842 | −2.336674 | −0.881948 |
| 1 | −2.384969 | −1.987131 | 0.155679 |
| 6 | 1.535698 | 2.445983 | −0.327057 |
| 6 | 2.618931 | 3.44704 | 0.181757 |
| 6 | 2.091954 | 4.571544 | 0.776143 |
| 6 | 1.29216 | 5.658092 | −0.266648 |
| 6 | 0.039409 | 5.147418 | −1.000484 |
| 6 | 0.299111 | 4.092637 | −1.725222 |
| 1 | 3.007038 | 3.874599 | −0.679575 |
| 1 | 1.479984 | 4.129557 | 1.511597 |
| 1 | 1.946563 | 6.163496 | −1.003832 |
| 1 | −0.670893 | 4.73268 | −0.294429 |
| 1 | 3.423746 | 2.894426 | 0.899078 |
| 1 | 2.960269 | 5.029022 | 1.325797 |
| 1 | 0.984958 | 6.412237 | 0.228535 |
| 1 | −0.461152 | 5.991064 | −1.750098 |
| 1 | −0.572886 | 3.955404 | −2.357117 |
| 1 | 1.160161 | 4.336101 | −2.367949 |
| 8 | 1.566778 | 1.312244 | −0.850435 |
| 8 | 0.488795 | 2.760234 | −0.834203 |
| 1 | −0.422562 | 0.581974 | 6.563288 |

| | | | |
|---|---|---|---|
| 6 | 2.54878 | 0.209753 | 2.91261 |
| 1 | 2.522481 | −0.225183 | 1.842068 |
| 6 | 3.684069 | −0.482965 | 3.094428 |
| 1 | 3.511651 | −1.561027 | 2.645639 |
| 1 | 4.641854 | −0.338374 | 2.617595 |
| 1 | 3.783705 | −0.071579 | 4.158892 |
| 6 | 2.832531 | 1.711921 | 3.353636 |
| 1 | 2.913198 | 2.21414 | 4.381183 |
| 1 | 3.780079 | 1.864142 | 2.709724 |
| 1 | 2.021952 | 2.173318 | 3.292199 |
| 6 | −2.203552 | −1.747269 | 2.957904 |
| 1 | −1.785298 | −2.225467 | 2.056253 |
| 6 | −2.746453 | −2.872448 | 3.882615 |
| 1 | −3.44363 | −3.501574 | 3.334803 |
| 1 | −1.941869 | −3.51132 | 4.269131 |
| 1 | −3.292232 | −2.473838 | 4.744429 |
| 6 | −3.34846 | −0.831167 | 2.51968 |
| 1 | −3.745243 | −0.242705 | 3.365664 |
| 1 | −3.010489 | −0.148745 | 1.719584 |
| 1 | −4.168151 | −1.433847 | 2.136399 |

**Table S26.** Cartesian coordinate of **$C_{eq}$TS1c.**

| The number of atom coordinates (angstroms) | X | Y | Z |
|---|---|---|---|
| 13 | −0.002386 | 0.14608 | 0.104325 |
| 8 | 0.595077 | −1.124279 | −1.125019 |
| 8 | −0.014573 | 1.776557 | 1.193753 |
| 7 | 0.458106 | −1.030921 | 1.627065 |
| 6 | 0.888096 | −2.365354 | −0.971284 |
| 6 | 1.044594 | −3.155573 | −2.241224 |
| 1 | 0.066492 | −3.214718 | −2.73288 |
| 1 | 1.723074 | −2.628617 | −2.920587 |
| 1 | 1.416495 | −4.167516 | −2.062762 |
| 6 | 1.041279 | −2.963425 | 0.27495 |
| 1 | 1.317264 | −4.010169 | 0.305137 |
| 6 | 0.83419 | −2.312484 | 1.508149 |
| 6 | 1.006448 | −3.179753 | 2.739672 |
| 1 | 0.037736 | −3.606373 | 3.022322 |
| 1 | 1.686219 | −4.008776 | 2.529878 |
| 1 | 1.376448 | −2.620818 | 3.599732 |
| 6 | 0.218644 | −0.539153 | 2.966219 |
| 6 | −1.013788 | −0.80376 | 3.60317 |
| 6 | −1.213827 | −0.295919 | 4.895164 |

| | | | |
|---|---|---|---|
| 1 | −2.162707 | −0.472596 | 5.393386 |
| 6 | −0.216107 | 0.410461 | 5.555651 |
| 6 | 1.000934 | 0.646894 | 4.917837 |
| 1 | 1.770762 | 1.204888 | 5.440862 |
| 6 | 1.233042 | 0.205549 | 3.61085 |
| 6 | −1.259137 | 2.304261 | 1.665741 |
| 1 | −1.960555 | 2.403479 | 0.832699 |
| 1 | −1.666263 | 1.55619 | 2.35459 |
| 6 | −1.099823 | 3.618324 | 2.389059 |
| 6 | −0.325866 | 3.699121 | 3.554047 |
| 1 | 0.139466 | 2.798656 | 3.938541 |
| 6 | −0.148231 | 4.918451 | 4.206935 |
| 1 | 0.462012 | 4.966647 | 5.105492 |
| 6 | −0.756502 | 6.075971 | 3.710547 |
| 1 | −0.616952 | 7.027398 | 4.217132 |
| 6 | −1.55205 | 6.000383 | 2.565769 |
| 1 | −2.035517 | 6.893218 | 2.177367 |
| 6 | −1.720887 | 4.776632 | 1.911869 |
| 1 | −2.327304 | 4.720214 | 1.011497 |
| 8 | −1.726291 | 0.105872 | −0.240454 |
| 6 | −2.298837 | −0.127877 | −1.49718 |
| 1 | −1.749095 | 0.388782 | −2.301776 |
| 1 | −3.31406 | 0.305304 | −1.499727 |
| 6 | −2.418544 | −1.599455 | −1.869806 |
| 6 | −2.50027 | −1.989168 | −3.212338 |
| 1 | −2.44396 | −1.233066 | −3.993381 |
| 6 | −2.637539 | −3.334933 | −3.560407 |
| 1 | −2.693764 | −3.619529 | −4.608355 |
| 6 | −2.685152 | −4.315246 | −2.564838 |
| 1 | −2.780927 | −5.364158 | −2.833302 |
| 6 | −2.602174 | −3.934382 | −1.223334 |
| 1 | −2.63214 | −4.688304 | −0.440161 |
| 6 | −2.476022 | −2.586871 | −0.881948 |
| 1 | −2.403375 | −2.283708 | 0.155679 |
| 6 | 0.812283 | 2.524596 | −0.327057 |
| 6 | 2.037476 | 3.256491 | 0.181757 |
| 6 | 1.874919 | 4.658632 | 0.776143 |
| 6 | 1.643386 | 5.760939 | −0.266648 |
| 6 | 0.301157 | 5.665286 | −1.000484 |
| 6 | 0.065913 | 4.345083 | −1.725222 |
| 1 | 2.721594 | 3.290692 | −0.679575 |
| 1 | 1.067976 | 4.66102 | 1.511597 |

| | | | |
|---|---|---|---|
| 1 | 2.460232 | 5.735931 | −1.003832 |
| 1 | −0.521853 | 5.819013 | −0.294429 |
| 1 | 2.500582 | 2.579542 | 0.899078 |
| 1 | 2.79655 | 4.887577 | 1.325797 |
| 1 | 1.702676 | 6.738319 | 0.228535 |
| 1 | 0.236753 | 6.466148 | −1.750098 |
| 1 | −0.82315 | 4.408562 | −2.357117 |
| 1 | 0.914759 | 4.078119 | −2.367949 |
| 8 | 1.037084 | 1.357709 | −0.850435 |
| 8 | −0.229826 | 3.240494 | −0.834203 |
| 1 | −0.383967 | 0.781182 | 6.563288 |
| 6 | 2.555385 | 0.506877 | 2.91261 |
| 1 | 2.341591 | 0.583599 | 1.842068 |
| 6 | 3.5748 | −0.634952 | 3.094428 |
| 1 | 3.224972 | −1.568122 | 2.645639 |
| 1 | 4.527189 | −0.374254 | 2.617595 |
| 1 | 3.766654 | −0.817336 | 4.158892 |
| 6 | 3.180135 | 1.841328 | 3.353636 |
| 1 | 3.559175 | 1.794795 | 4.381183 |
| 1 | 4.033619 | 2.08291 | 2.709724 |
| 1 | 2.459592 | 2.662978 | 3.292199 |
| 6 | −2.118405 | −1.637948 | 2.957904 |
| 1 | −1.707823 | −2.097994 | 2.056253 |
| 6 | −2.592461 | −2.77678 | 3.882615 |
| 1 | −3.26375 | −3.448174 | 3.334803 |
| 1 | −1.75556 | −3.368687 | 4.269131 |
| 1 | −3.148505 | −2.390451 | 4.744429 |
| 6 | −3.316255 | −0.774072 | 2.51968 |
| 1 | −3.715672 | −0.201399 | 3.365664 |
| 1 | −3.026639 | −0.089745 | 1.719584 |
| 1 | −4.120317 | −1.414618 | 2.136399 |

**Table S27.** Cartesian coordinate of **C$_{eq}$2c.**

| The number of atom coordinates (angstroms) | X | Y | Z |
|---|---|---|---|
| 13 | 0.07619 | 0.091962 | 0.124663 |
| 8 | 0.688216 | −1.096445 | −1.142702 |
| 8 | −0.041861 | 1.849915 | 1.351445 |
| 7 | 0.494017 | −1.069933 | 1.637535 |
| 6 | 0.933299 | −2.354334 | −1.002687 |
| 6 | 1.10154 | −3.12219 | −2.283094 |
| 1 | 0.13737 | −3.136783 | −2.804897 |
| 1 | 1.818278 | −2.604502 | −2.929469 |

| | | | |
|---|---|---|---|
| 1 | 1.432624 | −4.14987 | −2.116071 |
| 6 | 1.03161 | −2.981167 | 0.2346 |
| 1 | 1.273595 | −4.036651 | 0.244495 |
| 6 | 0.833102 | −2.357025 | 1.48581 |
| 6 | 0.985147 | −3.252524 | 2.697763 |
| 1 | 0.007651 | −3.661544 | 2.975354 |
| 1 | 1.645767 | −4.092495 | 2.471787 |
| 1 | 1.371689 | −2.714995 | 3.56461 |
| 6 | 0.259313 | −0.585541 | 2.980486 |
| 6 | −0.982 | −0.830407 | 3.607372 |
| 6 | −1.188 | −0.308263 | 4.893 |
| 1 | −2.142977 | −0.469828 | 5.384755 |
| 6 | −0.192125 | 0.403343 | 5.54921 |
| 6 | 1.032523 | 0.622649 | 4.919152 |
| 1 | 1.79845 | 1.182418 | 5.444648 |
| 6 | 1.278965 | 0.155992 | 3.623477 |
| 6 | −1.278028 | 2.378479 | 1.896201 |
| 1 | −2.031894 | 2.393282 | 1.109498 |
| 1 | −1.557065 | 1.626421 | 2.636377 |
| 6 | −1.10678 | 3.725405 | 2.544475 |
| 6 | −0.231058 | 3.880017 | 3.626625 |
| 1 | 0.305198 | 3.012485 | 3.999567 |
| 6 | −0.047873 | 5.130962 | 4.214326 |
| 1 | 0.640029 | 5.24197 | 5.048664 |
| 6 | −0.751877 | 6.240302 | 3.735093 |
| 1 | −0.609921 | 7.21556 | 4.193247 |
| 6 | −1.639296 | 6.090182 | 2.667451 |
| 1 | −2.188628 | 6.948635 | 2.289965 |
| 6 | −1.809905 | 4.837834 | 2.072231 |
| 1 | −2.474784 | 4.723508 | 1.220459 |
| 8 | −1.654696 | 0.114105 | −0.149378 |
| 6 | −2.280558 | −0.027491 | −1.39678 |
| 1 | −1.781124 | 0.569018 | −2.178642 |
| 1 | −3.302938 | 0.378454 | −1.314574 |
| 6 | −2.381367 | −1.468118 | −1.878735 |
| 6 | −2.426676 | −1.763965 | −3.246219 |
| 1 | −2.355515 | −0.955454 | −3.971303 |
| 6 | −2.547752 | −3.083207 | −3.689712 |
| 1 | −2.576391 | −3.294771 | −4.755816 |
| 6 | −2.614048 | −4.129888 | −2.765693 |
| 1 | −2.697376 | −5.158064 | −3.10815 |
| 6 | −2.564275 | −3.843047 | −1.399054 |

| | | | |
|---|---|---|---|
| 1 | −2.608301 | −4.649964 | −0.671443 |
| 6 | −2.453911 | −2.522246 | −0.962307 |
| 1 | −2.405892 | −2.292803 | 0.096129 |
| 6 | 0.649497 | 2.518335 | 0.218863 |
| 6 | 1.797834 | 3.431859 | 0.702755 |
| 6 | 1.581897 | 4.951642 | 0.685459 |
| 6 | 1.560793 | 5.576046 | −0.720117 |
| 6 | 0.290347 | 5.286698 | −1.529494 |
| 6 | −0.005661 | 3.801239 | −1.710885 |
| 1 | 2.662208 | 3.185747 | 0.075554 |
| 1 | 0.668197 | 5.216387 | 1.223294 |
| 1 | 2.43768 | 5.222025 | −1.28322 |
| 1 | −0.575379 | 5.743858 | −1.032068 |
| 1 | 2.045449 | 3.107803 | 1.714067 |
| 1 | 2.412231 | 5.398466 | 1.248106 |
| 1 | 1.673528 | 6.664416 | −0.627379 |
| 1 | 0.371079 | 5.749191 | −2.523369 |
| 1 | −0.862929 | 3.659417 | −2.377426 |
| 1 | 0.846024 | 3.264494 | −2.146565 |
| 8 | 1.126892 | 1.435691 | −0.453166 |
| 8 | −0.379115 | 3.193859 | −0.466419 |
| 1 | −0.366084 | 0.791702 | 6.549106 |
| 6 | 2.608604 | 0.443438 | 2.927419 |
| 1 | 2.378917 | 0.689512 | 1.88446 |
| 6 | 3.545685 | −0.782194 | 2.920142 |
| 1 | 3.140225 | −1.610898 | 2.336529 |
| 1 | 4.512086 | −0.51259 | 2.478318 |
| 1 | 3.727972 | −1.135489 | 3.942455 |
| 6 | 3.363565 | 1.641556 | 3.527603 |
| 1 | 3.792384 | 1.398633 | 4.507381 |
| 1 | 4.192535 | 1.921985 | 2.869147 |
| 1 | 2.71909 | 2.517819 | 3.651072 |
| 6 | −2.101399 | −1.636554 | 2.952352 |
| 1 | −1.707064 | −2.082705 | 2.036499 |
| 6 | −2.587681 | −2.785007 | 3.858263 |
| 1 | −3.284203 | −3.427044 | 3.306866 |
| 1 | −1.759568 | −3.405854 | 4.217063 |
| 1 | −3.119274 | −2.405164 | 4.738186 |
| 6 | −3.28537 | −0.740637 | 2.54272 |
| 1 | −3.682416 | −0.196306 | 3.408174 |
| 1 | −2.978643 | −0.030504 | 1.772399 |

| | | | |
|---|---|---|---|
| 1 | −4.09559 | −1.354536 | 2.130019 |

**Table S28.** Cartesian coordinate of **C$_{eq}$3c.**

| The number of atom coordinates (angstroms) | X | Y | Z |
|---|---|---|---|
| 13 | −0.045658 | 0.383742 | −0.578478 |
| 8 | 0.904745 | −0.849991 | −1.576439 |
| 8 | 0.672402 | 4.001313 | −0.567215 |
| 7 | 0.361198 | −0.504676 | 1.120965 |
| 6 | 1.135898 | −2.080983 | −1.263564 |
| 6 | 1.683171 | −2.938416 | −2.372074 |
| 1 | 2.686211 | −2.582425 | −2.637423 |
| 1 | 1.740051 | −3.992767 | −2.092543 |
| 1 | 1.053407 | −2.832341 | −3.259812 |
| 6 | 0.956647 | −2.588232 | 0.015879 |
| 1 | 1.154062 | −3.641089 | 0.174391 |
| 6 | 0.675076 | −1.809475 | 1.160359 |
| 6 | 0.819319 | −2.555426 | 2.476521 |
| 1 | 1.879413 | −2.701518 | 2.70574 |
| 1 | 0.364892 | −2.0416 | 3.320562 |
| 1 | 0.368953 | −3.547589 | 2.378394 |
| 6 | 0.332001 | 0.186129 | 2.391803 |
| 6 | −0.881021 | 0.290933 | 3.112327 |
| 6 | −0.830591 | 0.848123 | 4.397788 |
| 1 | −1.742034 | 0.933153 | 4.980173 |
| 6 | 0.370556 | 1.282141 | 4.951413 |
| 6 | 1.542798 | 1.216993 | 4.204547 |
| 1 | 2.46148 | 1.603429 | 4.631825 |
| 6 | 1.548905 | 0.694352 | 2.905377 |
| 6 | 1.640743 | 3.887902 | 0.491462 |
| 1 | 1.785819 | 2.837577 | 0.73347 |
| 1 | 2.58556 | 4.283043 | 0.103317 |
| 6 | 1.186022 | 4.670881 | 1.699306 |
| 6 | 1.508033 | 6.026645 | 1.833972 |
| 1 | 2.122216 | 6.507395 | 1.075699 |
| 6 | 1.045779 | 6.763262 | 2.926538 |
| 1 | 1.303125 | 7.815261 | 3.018582 |
| 6 | 0.257017 | 6.146952 | 3.901433 |
| 1 | −0.100593 | 6.718287 | 4.754195 |
| 6 | −0.064636 | 4.792844 | 3.777859 |
| 1 | −0.668063 | 4.298529 | 4.53447 |
| 6 | 0.394896 | 4.062273 | 2.682436 |

| | | | |
|---|---|---|---|
| 1 | 0.143119 | 3.013427 | 2.585452 |
| 8 | −1.697821 | 0.082716 | −1.109946 |
| 6 | −2.086812 | −0.750051 | −2.175387 |
| 1 | −1.412998 | −0.651453 | −3.041125 |
| 1 | −3.081374 | −0.420132 | −2.519132 |
| 6 | −2.168623 | −2.218506 | −1.791991 |
| 6 | −1.784806 | −3.226897 | −2.681599 |
| 1 | −1.420847 | −2.957524 | −3.671144 |
| 6 | −1.84762 | −4.572435 | −2.308557 |
| 1 | −1.534664 | −5.342667 | −3.009198 |
| 6 | −2.29906 | −4.925201 | −1.035307 |
| 1 | −2.341267 | −5.970373 | −0.739758 |
| 6 | −2.697352 | −3.923684 | −0.144292 |
| 1 | −3.054097 | −4.186638 | 0.848665 |
| 6 | −2.633837 | −2.583902 | −0.524471 |
| 1 | −2.92986 | −1.800711 | 0.163382 |
| 6 | 0.041893 | 2.834512 | −0.967902 |
| 6 | −0.878404 | 3.190875 | −2.144454 |
| 6 | −1.672225 | 4.495847 | −2.034178 |
| 6 | −2.803495 | 4.46163 | −1.001459 |
| 6 | −2.348416 | 4.115852 | 0.423143 |
| 6 | −1.995296 | 2.648102 | 0.651354 |
| 1 | −1.541974 | 2.334073 | −2.301623 |
| 1 | −0.981919 | 5.311796 | −1.794381 |
| 1 | −3.565209 | 3.732944 | −1.316546 |
| 1 | −1.510614 | 4.749711 | 0.728545 |
| 1 | −0.206492 | 3.242179 | −3.007804 |
| 1 | −2.099494 | 4.72069 | −3.020175 |
| 1 | −3.299051 | 5.441268 | −0.987704 |
| 1 | −3.173527 | 4.327263 | 1.117124 |
| 1 | −1.966494 | 2.450494 | 1.722786 |
| 1 | −2.738817 | 1.990009 | 0.1887 |
| 8 | 0.870359 | 1.790083 | −1.248906 |
| 8 | −0.692797 | 2.216543 | 0.194857 |
| 1 | 0.388555 | 1.696219 | 5.955853 |
| 6 | 2.833496 | 0.729694 | 2.069823 |
| 1 | 2.534575 | 0.870497 | 1.02436 |
| 6 | 3.751098 | 1.907788 | 2.453282 |
| 1 | 4.2802 | 1.713323 | 3.393826 |
| 1 | 4.511115 | 2.047914 | 1.677086 |
| 1 | 3.199803 | 2.84551 | 2.568741 |
| 6 | 3.654067 | −0.576115 | 2.128109 |

| | | | |
|---|---|---|---|
| 1 | 3.824744 | −0.883297 | 3.166961 |
| 1 | 3.16582 | −1.393969 | 1.595595 |
| 1 | 4.63173 | −0.421346 | 1.65722 |
| 6 | −2.209861 | −0.223362 | 2.55266 |
| 1 | −2.182737 | −0.102127 | 1.464851 |
| 6 | −3.434645 | 0.54729 | 3.082567 |
| 1 | −4.312808 | 0.29491 | 2.478353 |
| 1 | −3.665122 | 0.272812 | 4.118741 |
| 1 | −3.300646 | 1.632278 | 3.048508 |
| 6 | −2.424023 | −1.72463 | 2.845441 |
| 1 | −3.443142 | −2.018851 | 2.56727 |
| 1 | −1.740144 | −2.358542 | 2.280928 |
| 1 | −2.294237 | −1.93297 | 3.914112 |

**Table S29.** Cartesian coordinate of **$C_{eq}$TS2c.**

| The number of atom coordinates (angstroms) | X | Y | Z |
|---|---|---|---|
| 13 | −0.12611 | 0.508519 | −0.489562 |
| 8 | 0.90302 | −0.69211 | −1.490075 |
| 8 | 0.73052 | 4.067664 | −0.705804 |
| 7 | 0.281167 | −0.489013 | 1.1735 |
| 6 | 1.136607 | −1.934583 | −1.239375 |
| 6 | 1.716695 | −2.727319 | −2.38069 |
| 1 | 2.706079 | −2.325387 | −2.630473 |
| 1 | 1.810644 | −3.789854 | −2.144824 |
| 1 | 1.083868 | −2.608556 | −3.265098 |
| 6 | 0.932513 | −2.512922 | 0.005309 |
| 1 | 1.139062 | −3.570057 | 0.116832 |
| 6 | 0.617864 | −1.786708 | 1.174442 |
| 6 | 0.765526 | −2.575763 | 2.465034 |
| 1 | 1.82665 | −2.695542 | 2.705395 |
| 1 | 0.283483 | −2.107122 | 3.320051 |
| 1 | 0.349449 | −3.577848 | 2.324796 |
| 6 | 0.223991 | 0.161685 | 2.462595 |
| 6 | −0.99384 | 0.207654 | 3.180385 |
| 6 | −0.97359 | 0.754106 | 4.470561 |
| 1 | −1.890889 | 0.794345 | 5.048759 |
| 6 | 0.203699 | 1.243428 | 5.029768 |
| 6 | 1.38179 | 1.231812 | 4.288304 |
| 1 | 2.281198 | 1.654757 | 4.72213 |
| 6 | 1.41715 | 0.710571 | 2.989003 |
| 6 | 1.81258 | 4.009838 | 0.261319 |

| | | | |
|---|---|---|---|
| 1 | 2.037278 | 2.966495 | 0.471782 |
| 1 | 2.680807 | 4.467769 | −0.220706 |
| 6 | 1.404484 | 4.76543 | 1.498442 |
| 6 | 1.774063 | 6.102908 | 1.67706 |
| 1 | 2.378587 | 6.598719 | 0.92082 |
| 6 | 1.372794 | 6.80239 | 2.817566 |
| 1 | 1.666202 | 7.840771 | 2.9468 |
| 6 | 0.599256 | 6.165913 | 3.791046 |
| 1 | 0.290285 | 6.708566 | 4.680671 |
| 6 | 0.229219 | 4.828803 | 3.620469 |
| 1 | −0.361756 | 4.318719 | 4.376059 |
| 6 | 0.626518 | 4.13461 | 2.478808 |
| 1 | 0.340976 | 3.099506 | 2.338692 |
| 8 | −1.714565 | 0.137208 | −1.171936 |
| 6 | −2.013474 | −0.728484 | −2.234745 |
| 1 | −1.272778 | −0.653063 | −3.047031 |
| 1 | −2.97912 | −0.417585 | −2.66942 |
| 6 | −2.1233 | −2.189163 | −1.82464 |
| 6 | −1.739903 | −3.214114 | −2.696181 |
| 1 | −1.353451 | −2.961277 | −3.681688 |
| 6 | −1.831582 | −4.554244 | −2.311833 |
| 1 | −1.518265 | −5.336618 | −2.998849 |
| 6 | −2.313353 | −4.886404 | −1.043986 |
| 1 | −2.378705 | −5.927556 | −0.738447 |
| 6 | −2.711789 | −3.869097 | −0.171684 |
| 1 | −3.091995 | −4.116494 | 0.816382 |
| 6 | −2.618764 | −2.533638 | −0.562791 |
| 1 | −2.914528 | −1.739529 | 0.112749 |
| 6 | 0.211216 | 2.93054 | −1.170947 |
| 6 | −0.864639 | 3.190308 | −2.207243 |
| 6 | −1.785053 | 4.389936 | −1.968498 |
| 6 | −2.872667 | 4.175904 | −0.907575 |
| 6 | −2.374671 | 3.942834 | 0.5291 |
| 6 | −1.944697 | 2.512437 | 0.857151 |
| 1 | −1.436154 | 2.265132 | −2.31677 |
| 1 | −1.180076 | 5.265394 | −1.70566 |
| 1 | −3.51052 | 3.331285 | −1.206938 |
| 1 | −1.557489 | 4.632849 | 0.76873 |
| 1 | −0.300887 | 3.333771 | −3.139904 |
| 1 | −2.27582 | 4.624156 | −2.921991 |
| 1 | −3.518348 | 5.064383 | −0.912412 |
| 1 | −3.194765 | 4.177359 | 1.222254 |

| | | | |
|---|---|---|---|
| 1 | −1.846598 | 2.426274 | 1.945892 |
| 1 | −2.723954 | 1.812753 | 0.527562 |
| 8 | 0.937411 | 1.862387 | −1.280967 |
| 8 | −0.696712 | 2.138358 | 0.29548 |
| 1 | 0.199405 | 1.652455 | 6.036675 |
| 6 | 2.704653 | 0.788598 | 2.160677 |
| 1 | 2.405785 | 0.95342 | 1.118438 |
| 6 | 3.611177 | 1.962779 | 2.576322 |
| 1 | 4.127375 | 1.754605 | 3.521128 |
| 1 | 4.383353 | 2.116442 | 1.814328 |
| 1 | 3.055763 | 2.896464 | 2.69634 |
| 6 | 3.540815 | −0.508961 | 2.181635 |
| 1 | 3.708015 | −0.847332 | 3.211263 |
| 1 | 3.066811 | −1.314601 | 1.619252 |
| 1 | 4.519951 | −0.327062 | 1.723449 |
| 6 | −2.300238 | −0.334604 | 2.596533 |
| 1 | −2.248643 | −0.208031 | 1.510981 |
| 6 | −3.548234 | 0.416794 | 3.09732 |
| 1 | −4.411591 | 0.140074 | 2.482209 |
| 1 | −3.790804 | 0.149868 | 4.132854 |
| 1 | −3.431003 | 1.502699 | 3.048975 |
| 6 | −2.495693 | −1.840562 | 2.873344 |
| 1 | −3.503169 | −2.148371 | 2.568452 |
| 1 | −1.788973 | −2.457823 | 2.318631 |
| 1 | −2.386858 | −2.057548 | 3.942748 |

**Table S30.** Cartesian coordinate of $\mathbf{C_{eq}4c}$.

| The number of atom coordinates (angstroms) | X | Y | Z |
|---|---|---|---|
| 13 | 0.07619 | 0.091962 | 0.124663 |
| 8 | 0.688216 | −1.096445 | −1.142702 |
| 8 | −0.041861 | 1.849915 | 1.351445 |
| 7 | 0.494017 | −1.069933 | 1.637535 |
| 6 | 0.933299 | −2.354334 | −1.002687 |
| 6 | 1.10154 | −3.12219 | −2.283094 |
| 1 | 0.13737 | −3.136783 | −2.804897 |
| 1 | 1.818278 | −2.604502 | −2.929469 |
| 1 | 1.432624 | −4.14987 | −2.116071 |
| 6 | 1.03161 | −2.981167 | 0.2346 |
| 1 | 1.273595 | −4.036651 | 0.244495 |
| 6 | 0.833102 | −2.357025 | 1.48581 |
| 6 | 0.985147 | −3.252524 | 2.697763 |

| | | | |
|---|---|---|---|
| 1 | 0.007651 | −3.661544 | 2.975354 |
| 1 | 1.645767 | −4.092495 | 2.471787 |
| 1 | 1.371689 | −2.714995 | 3.56461 |
| 6 | 0.259313 | −0.585541 | 2.980486 |
| 6 | −0.982 | −0.830407 | 3.607372 |
| 6 | −1.188 | −0.308263 | 4.893 |
| 1 | −2.142977 | −0.469828 | 5.384755 |
| 6 | −0.192125 | 0.403343 | 5.54921 |
| 6 | 1.032523 | 0.622649 | 4.919152 |
| 1 | 1.79845 | 1.182418 | 5.444648 |
| 6 | 1.278965 | 0.155992 | 3.623477 |
| 6 | −1.278028 | 2.378479 | 1.896201 |
| 1 | −2.031894 | 2.393282 | 1.109498 |
| 1 | −1.557065 | 1.626421 | 2.636377 |
| 6 | −1.10678 | 3.725405 | 2.544475 |
| 6 | −0.231058 | 3.880017 | 3.626625 |
| 1 | 0.305198 | 3.012485 | 3.999567 |
| 6 | −0.047873 | 5.130962 | 4.214326 |
| 1 | 0.640029 | 5.24197 | 5.048664 |
| 6 | −0.751877 | 6.240302 | 3.735093 |
| 1 | −0.609921 | 7.21556 | 4.193247 |
| 6 | −1.639296 | 6.090182 | 2.667451 |
| 1 | −2.188628 | 6.948635 | 2.289965 |
| 6 | −1.809905 | 4.837834 | 2.072231 |
| 1 | −2.474784 | 4.723508 | 1.220459 |
| 8 | −1.654696 | 0.114105 | −0.149378 |
| 6 | −2.280558 | −0.027491 | −1.39678 |
| 1 | −1.781124 | 0.569018 | −2.178642 |
| 1 | −3.302938 | 0.378454 | −1.314574 |
| 6 | −2.381367 | −1.468118 | −1.878735 |
| 6 | −2.426676 | −1.763965 | −3.246219 |
| 1 | −2.355515 | −0.955454 | −3.971303 |
| 6 | −2.547752 | −3.083207 | −3.689712 |
| 1 | −2.576391 | −3.294771 | −4.755816 |
| 6 | −2.614048 | −4.129888 | −2.765693 |
| 1 | −2.697376 | −5.158064 | −3.10815 |
| 6 | −2.564275 | −3.843047 | −1.399054 |
| 1 | −2.608301 | −4.649964 | −0.671443 |
| 6 | −2.453911 | −2.522246 | −0.962307 |
| 1 | −2.405892 | −2.292803 | 0.096129 |
| 6 | 0.649497 | 2.518335 | 0.218863 |
| 6 | 1.797834 | 3.431859 | 0.702755 |

| | | | |
|---|---|---|---|
| 6 | 1.581897 | 4.951642 | 0.685459 |
| 6 | 1.560793 | 5.576046 | −0.720117 |
| 6 | 0.290347 | 5.286698 | −1.529494 |
| 6 | −0.005661 | 3.801239 | −1.710885 |
| 1 | 2.662208 | 3.185747 | 0.075554 |
| 1 | 0.668197 | 5.216387 | 1.223294 |
| 1 | 2.43768 | 5.222025 | −1.28322 |
| 1 | −0.575379 | 5.743858 | −1.032068 |
| 1 | 2.045449 | 3.107803 | 1.714067 |
| 1 | 2.412231 | 5.398466 | 1.248106 |
| 1 | 1.673528 | 6.664416 | −0.627379 |
| 1 | 0.371079 | 5.749191 | −2.523369 |
| 1 | −0.862929 | 3.659417 | −2.377426 |
| 1 | 0.846024 | 3.264494 | −2.146565 |
| 8 | 1.126892 | 1.435691 | −0.453166 |
| 8 | −0.379115 | 3.193859 | −0.466419 |
| 1 | −0.366084 | 0.791702 | 6.549106 |
| 6 | 2.608604 | 0.443438 | 2.927419 |
| 1 | 2.378917 | 0.689512 | 1.88446 |
| 6 | 3.545685 | −0.782194 | 2.920142 |
| 1 | 3.140225 | −1.610898 | 2.336529 |
| 1 | 4.512086 | −0.51259 | 2.478318 |
| 1 | 3.727972 | −1.135489 | 3.942455 |
| 6 | 3.363565 | 1.641556 | 3.527603 |
| 1 | 3.792384 | 1.398633 | 4.507381 |
| 1 | 4.192535 | 1.921985 | 2.869147 |
| 1 | 2.71909 | 2.517819 | 3.651072 |
| 6 | −2.101399 | −1.636554 | 2.952352 |
| 1 | −1.707064 | −2.082705 | 2.036499 |
| 6 | −2.587681 | −2.785007 | 3.858263 |
| 1 | −3.284203 | −3.427044 | 3.306866 |
| 1 | −1.759568 | −3.405854 | 4.217063 |
| 1 | −3.119274 | −2.405164 | 4.738186 |
| 6 | −3.28537 | −0.740637 | 2.54272 |
| 1 | −3.682416 | −0.196306 | 3.408174 |
| 1 | −2.978643 | −0.030504 | 1.772399 |
| 1 | −4.09559 | −1.354536 | 2.130019 |

**Table S31.** Cartesian coordinate of **C$_{ax}$1c.**

| The number of atom coordinates (angstroms) | X | Y | Z |
|---|---|---|---|
| 13 | 0.277016 | −0.110945 | −0.653733 |

| | | | |
|---|---|---|---|
| 8 | 0.47808 | −1.779598 | −1.480675 |
| 8 | 1.405712 | 0.588569 | −1.820673 |
| 7 | 0.927237 | −0.833175 | 1.085021 |
| 6 | 0.932138 | −2.891977 | −1.045728 |
| 6 | 0.836659 | −4.049233 | −2.003655 |
| 1 | 1.317905 | −4.953485 | −1.622485 |
| 1 | 1.286114 | −3.769769 | −2.962472 |
| 1 | −0.225013 | −4.254573 | −2.186324 |
| 6 | 1.447779 | −3.055824 | 0.239783 |
| 1 | 1.842333 | −4.028469 | 0.507408 |
| 6 | 1.412467 | −2.074326 | 1.249421 |
| 6 | 1.899949 | −2.530194 | 2.612067 |
| 1 | 2.346046 | −1.72128 | 3.191545 |
| 1 | 1.057846 | −2.919786 | 3.195023 |
| 1 | 2.627891 | −3.337771 | 2.503363 |
| 6 | 0.821951 | −0.00809 | 2.267873 |
| 6 | −0.26526 | −0.18055 | 3.152861 |
| 6 | −0.363684 | 0.674342 | 4.259939 |
| 1 | −1.200374 | 0.562384 | 4.943891 |
| 6 | 0.592294 | 1.653854 | 4.505308 |
| 6 | 1.668249 | 1.800515 | 3.630751 |
| 1 | 2.41346 | 2.563481 | 3.832363 |
| 6 | 1.797856 | 0.990701 | 2.497626 |
| 6 | 1.932536 | 1.854062 | −2.045259 |
| 1 | 2.43558 | 2.251594 | −1.149174 |
| 1 | 2.718754 | 1.775569 | −2.816727 |
| 6 | 0.947014 | 2.924854 | −2.504144 |
| 6 | −0.382909 | 2.631729 | −2.815322 |
| 1 | −0.728533 | 1.60834 | −2.737657 |
| 6 | −1.268774 | 3.647371 | −3.186559 |
| 1 | −2.302709 | 3.399542 | −3.415471 |
| 6 | −0.833777 | 4.972136 | −3.263654 |
| 1 | −1.523008 | 5.761836 | −3.551868 |
| 6 | 0.50074 | 5.2728 | −2.972376 |
| 1 | 0.854127 | 6.299509 | −3.03242 |
| 6 | 1.379218 | 4.256027 | −2.596937 |
| 1 | 2.414052 | 4.498081 | −2.357609 |
| 8 | −1.479339 | −0.015226 | −0.867215 |
| 6 | −2.200724 | −0.643267 | −1.888578 |
| 1 | −1.71219 | −0.526055 | −2.872151 |
| 1 | −3.178413 | −0.13703 | −1.980086 |
| 6 | −2.475061 | −2.130668 | −1.693663 |

| | | | |
|---|---|---|---|
| 6 | −2.894229 | −2.912278 | −2.777701 |
| 1 | −2.997294 | −2.45419 | −3.7601 |
| 6 | −3.163042 | −4.273329 | −2.618976 |
| 1 | −3.480096 | −4.865053 | −3.474499 |
| 6 | −3.011381 | −4.8778 | −1.367218 |
| 1 | −3.211214 | −5.939059 | −1.243143 |
| 6 | −2.592259 | −4.104991 | −0.281597 |
| 1 | −2.4595 | −4.563127 | 0.695939 |
| 6 | −2.332476 | −2.743695 | −0.447238 |
| 1 | −1.986036 | −2.143651 | 0.382497 |
| 6 | −0.694887 | 2.515154 | 0.589901 |
| 6 | −0.440402 | 4.000434 | 0.54797 |
| 6 | −0.31808 | 4.630174 | 1.954154 |
| 6 | −1.660487 | 4.870882 | 2.657066 |
| 6 | −2.501997 | 3.6065 | 2.872323 |
| 6 | −2.883083 | 2.882812 | 1.584875 |
| 1 | −1.238235 | 4.48735 | −0.026876 |
| 1 | 0.32491 | 3.995158 | 2.575854 |
| 1 | −2.246867 | 5.589898 | 2.066628 |
| 1 | −1.972897 | 2.892564 | 3.515762 |
| 1 | 0.480403 | 4.134144 | −0.018647 |
| 1 | 0.199974 | 5.589238 | 1.840505 |
| 1 | −1.473777 | 5.345572 | 3.628084 |
| 1 | −3.433541 | 3.875569 | 3.38618 |
| 1 | −3.689302 | 2.16925 | 1.757506 |
| 1 | −3.19546 | 3.574255 | 0.794358 |
| 8 | 0.170304 | 1.724691 | 0.20945 |
| 8 | −1.816271 | 2.035917 | 1.085277 |
| 1 | 0.504244 | 2.298933 | 5.375809 |
| 6 | 2.973998 | 1.182787 | 1.545009 |
| 1 | 2.666962 | 0.819276 | 0.561038 |
| 6 | 4.19145 | 0.345396 | 1.981974 |
| 1 | 3.965329 | −0.724629 | 1.97956 |
| 1 | 5.033055 | 0.509711 | 1.298689 |
| 1 | 4.513263 | 0.624095 | 2.993117 |
| 6 | 3.373815 | 2.658238 | 1.379448 |
| 1 | 3.828761 | 3.068178 | 2.288944 |
| 1 | 4.112608 | 2.755842 | 0.576373 |
| 1 | 2.50654 | 3.274862 | 1.122135 |
| 6 | −1.327183 | −1.25956 | 2.958306 |
| 1 | −0.977448 | −1.942544 | 2.180054 |
| 6 | −1.544884 | −2.096761 | 4.233581 |

| | | | |
|---|---|---|---|
| 1 | −2.199211 | −2.948574 | 4.01471 |
| 1 | −0.601925 | −2.483567 | 4.635529 |
| 1 | −2.025723 | −1.510611 | 5.025364 |
| 6 | −2.661299 | −0.661772 | 2.472599 |
| 1 | −3.033198 | 0.080705 | 3.190228 |
| 1 | −2.535716 | −0.180408 | 1.500745 |
| 1 | −3.418086 | −1.44995 | 2.37882 |

**Table S32.** Cartesian coordinate of **$C_{ax}$TS1c.**

| The number of atom coordinates (angstroms) | X | Y | Z |
|---|---|---|---|
| 13 | 0.777623 | 0.111364 | −0.380736 |
| 8 | 0.569348 | −1.48934 | −1.325597 |
| 8 | 2.14914 | 0.632858 | −1.355941 |
| 7 | 1.353148 | −0.862972 | 1.220991 |
| 6 | 1.374236 | −2.496983 | −1.207527 |
| 6 | 1.379221 | −3.448429 | −2.374998 |
| 1 | 2.040921 | −4.304042 | −2.21985 |
| 1 | 1.691264 | −2.909797 | −3.277215 |
| 1 | 0.359325 | −3.810129 | −2.553772 |
| 6 | 2.14822 | −2.733823 | −0.08149 |
| 1 | 2.779044 | −3.614717 | −0.07218 |
| 6 | 2.036878 | −2.00032 | 1.12881 |
| 6 | 2.689032 | −2.624146 | 2.34315 |
| 1 | 2.850972 | −1.905099 | 3.146008 |
| 1 | 2.052643 | −3.426754 | 2.733197 |
| 1 | 3.644653 | −3.075491 | 2.060003 |
| 6 | 1.078232 | −0.313671 | 2.528523 |
| 6 | −0.011884 | −0.820405 | 3.266195 |
| 6 | −0.280006 | −0.255778 | 4.520037 |
| 1 | −1.116711 | −0.632414 | 5.102325 |
| 6 | 0.504091 | 0.771968 | 5.032842 |
| 6 | 1.571863 | 1.262238 | 4.285044 |
| 1 | 2.1826 | 2.064959 | 4.68854 |
| 6 | 1.872149 | 0.74323 | 3.021502 |
| 6 | 2.576975 | 1.94775 | −1.568068 |
| 1 | 2.699459 | 2.501396 | −0.625411 |
| 1 | 3.57291 | 1.917969 | −2.042617 |
| 6 | 1.642769 | 2.750268 | −2.462135 |
| 6 | 0.823241 | 2.108378 | −3.398334 |
| 1 | 0.872602 | 1.026687 | −3.479319 |
| 6 | −0.062594 | 2.841175 | −4.191117 |

| | | | |
|---|---|---|---|
| 1 | −0.700671 | 2.326301 | −4.905545 |
| 6 | −0.135483 | 4.230976 | −4.064809 |
| 1 | −0.830497 | 4.801502 | −4.675467 |
| 6 | 0.690803 | 4.882106 | −3.14444 |
| 1 | 0.63875 | 5.962618 | −3.034518 |
| 6 | 1.571086 | 4.144339 | −2.350144 |
| 1 | 2.190713 | 4.653045 | −1.614187 |
| 8 | −1.04429 | 0.488455 | −0.693915 |
| 6 | −2.029301 | 0.099262 | −1.653739 |
| 1 | −1.487652 | −0.338266 | −2.497901 |
| 1 | −2.538295 | 1.000647 | −2.013398 |
| 6 | −3.021782 | −0.890846 | −1.092881 |
| 6 | −4.318018 | −0.497212 | −0.745742 |
| 1 | −4.623202 | 0.53617 | −0.896733 |
| 6 | −5.227315 | −1.419354 | −0.22095 |
| 1 | −6.231515 | −1.099457 | 0.044446 |
| 6 | −4.846845 | −2.751035 | −0.047459 |
| 1 | −5.553523 | −3.472032 | 0.354851 |
| 6 | −3.553193 | −3.153786 | −0.394555 |
| 1 | −3.252585 | −4.189924 | −0.261493 |
| 6 | −2.646333 | −2.228932 | −0.909355 |
| 1 | −1.636512 | −2.529493 | −1.171042 |
| 6 | −0.916363 | 1.867186 | 0.318153 |
| 6 | −1.380476 | 3.034656 | −0.532421 |
| 6 | −1.364825 | 4.355893 | 0.259889 |
| 6 | −2.646036 | 4.589612 | 1.074651 |
| 6 | −2.925052 | 3.536796 | 2.157098 |
| 6 | −2.954897 | 2.093723 | 1.654255 |
| 1 | −2.386512 | 2.856005 | −0.927883 |
| 1 | −0.482217 | 4.374974 | 0.912058 |
| 1 | −3.498498 | 4.615022 | 0.379361 |
| 1 | −2.164421 | 3.588763 | 2.946583 |
| 1 | −0.702209 | 3.083665 | −1.381003 |
| 1 | −1.241595 | 5.177984 | −0.454713 |
| 1 | −2.603142 | 5.580024 | 1.545545 |
| 1 | −3.892201 | 3.754249 | 2.630106 |
| 1 | −3.371673 | 1.431173 | 2.416367 |
| 1 | −3.567177 | 1.981214 | 0.751528 |
| 8 | 0.370392 | 1.720267 | 0.508228 |
| 8 | −1.628149 | 1.582591 | 1.444586 |
| 1 | 0.284165 | 1.191486 | 6.011334 |
| 6 | 3.052235 | 1.304471 | 2.236188 |

| | | | |
|---|---|---|---|
| 1 | 3.011851 | 0.899456 | 1.221439 |
| 6 | 4.391125 | 0.859873 | 2.855968 |
| 1 | 4.478637 | −0.231717 | 2.885949 |
| 1 | 5.233096 | 1.249836 | 2.271407 |
| 1 | 4.491192 | 1.232377 | 3.882976 |
| 6 | 2.986054 | 2.837306 | 2.117184 |
| 1 | 3.093768 | 3.327512 | 3.092255 |
| 1 | 3.799622 | 3.200263 | 1.477474 |
| 1 | 2.032306 | 3.138644 | 1.676593 |
| 6 | −0.909655 | −1.944319 | 2.754669 |
| 1 | −0.539865 | −2.266758 | 1.776886 |
| 6 | −0.87781 | −3.166494 | 3.69292 |
| 1 | −1.453713 | −3.993219 | 3.26014 |
| 1 | 0.144328 | −3.517482 | 3.870808 |
| 1 | −1.319142 | −2.930099 | 4.66841 |
| 6 | −2.356855 | −1.46085 | 2.550963 |
| 1 | −2.775979 | −1.06507 | 3.484316 |
| 1 | −2.393454 | −0.676397 | 1.794289 |
| 1 | −2.993203 | −2.285586 | 2.215555 |

**Table S33.** Cartesian coordinate of **C$_{ax}$2c.**

| The number of atom coordinates (angstroms) | X | Y | Z |
|---|---|---|---|
| 13 | 0.584859 | 0.298107 | 0.180144 |
| 8 | 0.181982 | −1.173617 | −0.825917 |
| 8 | 1.926984 | 1.145455 | −0.555826 |
| 7 | 1.586361 | −0.787925 | 1.482054 |
| 6 | 1.012027 | −2.162778 | −1.019827 |
| 6 | 0.726935 | −2.972241 | −2.255243 |
| 1 | 1.437554 | −3.789776 | −2.397344 |
| 1 | 0.748686 | −2.312098 | −3.130305 |
| 1 | −0.288189 | −3.382434 | −2.193292 |
| 6 | 2.050524 | −2.478506 | −0.164265 |
| 1 | 2.675832 | −3.325999 | −0.416732 |
| 6 | 2.245544 | −1.872941 | 1.112716 |
| 6 | 3.192111 | −2.55323 | 2.074406 |
| 1 | 3.86935 | −1.825609 | 2.531341 |
| 1 | 2.630921 | −3.019167 | 2.891563 |
| 1 | 3.777468 | −3.326029 | 1.572117 |
| 6 | 1.622223 | −0.374854 | 2.864318 |
| 6 | 0.690948 | −0.944692 | 3.759155 |
| 6 | 0.704001 | −0.51082 | 5.089416 |

| | | | |
|---|---|---|---|
| 1 | −0.007933 | −0.934286 | 5.792832 |
| 6 | 1.601614 | 0.461416 | 5.523463 |
| 6 | 2.499099 | 1.023245 | 4.620288 |
| 1 | 3.187347 | 1.793537 | 4.957634 |
| 6 | 2.526367 | 0.622901 | 3.278394 |
| 6 | 1.946715 | 2.287515 | −1.359645 |
| 1 | 1.429236 | 3.136634 | −0.88118 |
| 1 | 2.995605 | 2.607079 | −1.480826 |
| 6 | 1.342747 | 2.071473 | −2.738759 |
| 6 | 1.456787 | 0.826668 | −3.370648 |
| 1 | 1.981113 | 0.026974 | −2.857314 |
| 6 | 0.882645 | 0.609487 | −4.623957 |
| 1 | 0.972011 | −0.36498 | −5.09847 |
| 6 | 0.189607 | 1.639054 | −5.268421 |
| 1 | −0.265346 | 1.468199 | −6.240465 |
| 6 | 0.084458 | 2.888041 | −4.652152 |
| 1 | −0.455649 | 3.693796 | −5.143157 |
| 6 | 0.657284 | 3.098963 | −3.395437 |
| 1 | 0.55204 | 4.067127 | −2.909275 |
| 8 | −1.023195 | 1.230531 | −0.676753 |
| 6 | −1.847582 | 1.31503 | −1.848673 |
| 1 | −1.188196 | 0.990143 | −2.656506 |
| 1 | −2.092802 | 2.367292 | −2.032329 |
| 6 | −3.100277 | 0.469025 | −1.812972 |
| 6 | −4.303702 | 0.994137 | −2.292814 |
| 1 | −4.337649 | 2.022128 | −2.648633 |
| 6 | −5.46444 | 0.215232 | −2.314095 |
| 1 | −6.393173 | 0.638186 | −2.688227 |
| 6 | −5.429324 | −1.097257 | −1.843097 |
| 1 | −6.330793 | −1.704398 | −1.848879 |
| 6 | −4.227114 | −1.626999 | −1.360824 |
| 1 | −4.19486 | −2.648475 | −0.9901 |
| 6 | −3.068511 | −0.852934 | −1.350636 |
| 1 | −2.135435 | −1.25836 | −0.975601 |
| 6 | −1.42231 | 1.718576 | 0.690547 |
| 6 | −1.405183 | 3.248124 | 0.690547 |
| 6 | −1.749932 | 3.847819 | 2.06456 |
| 6 | −3.258374 | 3.989768 | 2.317576 |
| 6 | −4.03885 | 2.66854 | 2.325895 |
| 6 | −3.862882 | 1.826252 | 1.062926 |
| 1 | −2.075188 | 3.646083 | −0.081537 |
| 1 | −1.282646 | 3.23105 | 2.842037 |

| | | | |
|---|---|---|---|
| 1 | −3.682238 | 4.645006 | 1.541457 |
| 1 | −3.73495 | 2.050503 | 3.180517 |
| 1 | −0.387526 | 3.521963 | 0.400658 |
| 1 | −1.29111 | 4.841564 | 2.13507 |
| 1 | −3.416651 | 4.504355 | 3.274107 |
| 1 | −5.108945 | 2.884092 | 2.450305 |
| 1 | −4.616059 | 1.035425 | 1.016188 |
| 1 | −3.97903 | 2.43069 | 0.154892 |
| 8 | −0.41068 | 1.172516 | 1.405747 |
| 8 | −2.618795 | 1.114804 | 1.060001 |
| 1 | 1.595076 | 0.786154 | 6.560828 |
| 6 | 3.485988 | 1.297137 | 2.305884 |
| 1 | 3.41518 | 0.788092 | 1.342885 |
| 6 | 4.949311 | 1.218142 | 2.774964 |
| 1 | 5.263159 | 0.179812 | 2.935605 |
| 1 | 5.612318 | 1.659274 | 2.021231 |
| 1 | 5.108066 | 1.762209 | 3.713615 |
| 6 | 3.056556 | 2.756611 | 2.063131 |
| 1 | 3.133329 | 3.350052 | 2.982886 |
| 1 | 3.694388 | 3.221275 | 1.301689 |
| 1 | 2.020964 | 2.797306 | 1.713721 |
| 6 | −0.343651 | −1.974458 | 3.314965 |
| 1 | −0.154852 | −2.221616 | 2.266429 |
| 6 | −0.242494 | −3.282317 | 4.121661 |
| 1 | −0.95449 | −4.021949 | 3.736609 |
| 1 | 0.761872 | −3.718038 | 4.065985 |
| 1 | −0.474964 | −3.119761 | 5.180623 |
| 6 | −1.765945 | −1.386968 | 3.391156 |
| 1 | −2.027408 | −1.128576 | 4.425017 |
| 1 | −1.846951 | −0.487467 | 2.776197 |
| 1 | −2.499553 | −2.120974 | 3.034175 |

**Table S34.** Cartesian coordinate of **C$_{ax}$3c.**

| The number of atom coordinates (angstroms) | X | Y | Z |
|---|---|---|---|
| 13 | 0.078781 | 0.114999 | 0.426592 |
| 8 | 0.090086 | −1.409709 | −0.55 |
| 8 | 1.313077 | 1.289642 | 0.03931 |
| 7 | 0.948704 | −0.716965 | 1.998153 |
| 6 | 0.797084 | −2.488862 | −0.351637 |
| 6 | 0.781043 | −3.453585 | −1.504346 |
| 1 | 1.348626 | −4.364182 | −1.297928 |

| | | | |
|---|---|---|---|
| 1 | 1.20013 | −2.95582 | −2.386564 |
| 1 | −0.255796 | −3.718962 | −1.741995 |
| 6 | 1.496873 | −2.747683 | 0.806098 |
| 1 | 2.031613 | −3.687819 | 0.876093 |
| 6 | 1.520959 | −1.907836 | 1.961978 |
| 6 | 2.250799 | −2.48805 | 3.154391 |
| 1 | 3.30061 | −2.65645 | 2.888455 |
| 1 | 2.209591 | −1.8483 | 4.034074 |
| 1 | 1.822766 | −3.4653 | 3.402751 |
| 6 | 1.014409 | 0.063959 | 3.215778 |
| 6 | −0.046041 | −0.027457 | 4.142834 |
| 6 | 0.057905 | 0.697036 | 5.33501 |
| 1 | −0.742289 | 0.63303 | 6.066211 |
| 6 | 1.164987 | 1.496619 | 5.601056 |
| 6 | 2.185382 | 1.596961 | 4.661301 |
| 1 | 3.044596 | 2.229221 | 4.868699 |
| 6 | 2.134439 | 0.889891 | 3.452903 |
| 6 | 1.565448 | 1.927618 | −1.180733 |
| 1 | 0.680171 | 2.475272 | −1.543802 |
| 1 | 2.350039 | 2.684014 | −1.013103 |
| 6 | 2.031872 | 0.977108 | −2.274376 |
| 6 | 2.793446 | −0.153731 | −1.955194 |
| 1 | 3.026376 | −0.354679 | −0.913771 |
| 6 | 3.225772 | −1.02676 | −2.954023 |
| 1 | 3.813638 | −1.901919 | −2.687666 |
| 6 | 2.903244 | −0.781026 | −4.292191 |
| 1 | 3.235402 | −1.463512 | −5.070098 |
| 6 | 2.150485 | 0.348048 | −4.621449 |
| 1 | 1.89126 | 0.547477 | −5.658201 |
| 6 | 1.72036 | 1.219017 | −3.617282 |
| 1 | 1.130616 | 2.095478 | −3.880206 |
| 8 | −2.639033 | 2.394901 | 0.265371 |
| 6 | −1.625894 | 3.30234 | 0.720979 |
| 1 | −1.847368 | 4.244651 | 0.205634 |
| 1 | −0.633565 | 2.972382 | 0.400345 |
| 6 | −1.660571 | 3.512629 | 2.218103 |
| 6 | −0.481002 | 3.763553 | 2.924572 |
| 1 | 0.474628 | 3.725984 | 2.407319 |
| 6 | −0.520482 | 4.038756 | 4.292211 |
| 1 | 0.405386 | 4.219991 | 4.829013 |
| 6 | −1.739051 | 4.043956 | 4.972532 |
| 1 | −1.768042 | 4.24599 | 6.040233 |

| | | | |
|---|---|---|---|
| 6 | −2.919231 | 3.771538 | 4.275852 |
| 1 | −3.871587 | 3.760964 | 4.800355 |
| 6 | −2.879685 | 3.513489 | 2.905481 |
| 1 | −3.791789 | 3.292254 | 2.359276 |
| 6 | −2.259036 | 1.049552 | 0.187454 |
| 6 | −3.556017 | 0.249045 | 0.040942 |
| 6 | −3.39036 | −1.151346 | −0.567728 |
| 6 | −3.387046 | −1.162645 | −2.107872 |
| 6 | −2.154345 | −0.535066 | −2.775067 |
| 6 | −1.823438 | 0.877631 | −2.306655 |
| 1 | −4.272385 | 0.835519 | −0.545617 |
| 1 | −2.47607 | −1.618566 | −0.184127 |
| 1 | −4.291623 | −0.643984 | −2.4594 |
| 1 | −1.270253 | −1.152704 | −2.587972 |
| 1 | −3.955361 | 0.184711 | 1.058215 |
| 1 | −4.226711 | −1.773031 | −0.225049 |
| 1 | −3.475397 | −2.200178 | −2.456404 |
| 1 | −2.307049 | −0.503833 | −3.862822 |
| 1 | −1.015359 | 1.298246 | −2.910247 |
| 1 | −2.683467 | 1.555669 | −2.361644 |
| 8 | −1.457989 | 0.616952 | 1.199838 |
| 8 | −1.338112 | 0.884304 | −0.953842 |
| 1 | 1.225667 | 2.050085 | 6.534501 |
| 6 | 3.297852 | 1.006861 | 2.473552 |
| 1 | 3.057466 | 0.420411 | 1.585419 |
| 6 | 4.6001 | 0.452173 | 3.08161 |
| 1 | 4.480431 | −0.585539 | 3.411438 |
| 1 | 5.410196 | 0.48466 | 2.343085 |
| 1 | 4.91754 | 1.041006 | 3.950492 |
| 6 | 3.486812 | 2.458168 | 1.998218 |
| 1 | 3.738662 | 3.128021 | 2.829175 |
| 1 | 4.301789 | 2.513136 | 1.265953 |
| 1 | 2.57258 | 2.813919 | 1.517156 |
| 6 | −1.255326 | −0.926355 | 3.912301 |
| 1 | −1.234228 | −1.263713 | 2.874344 |
| 6 | −1.187987 | −2.171607 | 4.817426 |
| 1 | −2.042247 | −2.832565 | 4.626852 |
| 1 | −0.26947 | −2.744379 | 4.646145 |
| 1 | −1.210601 | −1.887926 | 5.876739 |
| 6 | −2.58 | −0.169481 | 4.105823 |
| 1 | −2.727518 | 0.138064 | 5.148046 |
| 1 | −2.602827 | 0.721067 | 3.474831 |

| | | | |
|---|---|---|---|
| 1 | −3.422303 | −0.817079 | 3.831556 |

**Table S35.** Cartesian coordinate of **C$_{ax}$TS2c.**

| The number of atom coordinates (angstroms) | X | Y | Z |
|---|---|---|---|
| 13 | −0.031196 | 0.058608 | 0.391858 |
| 8 | 0.007498 | −1.50685 | −0.529699 |
| 8 | 1.30778 | 1.153196 | 0.067111 |
| 7 | 0.891924 | −0.778219 | 1.97579 |
| 6 | 0.926858 | −2.433116 | −0.437306 |
| 6 | 0.996555 | −3.348773 | −1.628003 |
| 1 | 1.735929 | −4.144022 | −1.504122 |
| 1 | 1.248901 | −2.75471 | −2.514012 |
| 1 | 0.010758 | −3.795111 | −1.803326 |
| 6 | 1.762083 | −2.586554 | 0.647197 |
| 1 | 2.474818 | −3.402996 | 0.627938 |
| 6 | 1.675243 | −1.830284 | 1.85881 |
| 6 | 2.538324 | −2.322474 | 2.999615 |
| 1 | 3.590984 | −2.13562 | 2.754622 |
| 1 | 2.310458 | −1.825766 | 3.942137 |
| 1 | 2.42215 | −3.403818 | 3.123691 |
| 6 | 0.737326 | −0.115799 | 3.248587 |
| 6 | −0.351731 | −0.494032 | 4.065178 |
| 6 | −0.546618 | 0.189123 | 5.269193 |
| 6 | −1.372293 | −0.094042 | 5.915779 |
| 6 | 0.296634 | 1.230483 | 5.651818 |
| 1 | 1.362849 | 1.588769 | 4.833653 |
| 6 | 2.025149 | 2.394056 | 5.140011 |
| 6 | 1.610879 | 0.92591 | 3.622969 |
| 1 | 1.501008 | 1.935362 | −1.076143 |
| 1 | 0.585642 | 2.481997 | −1.358657 |
| 6 | 2.261566 | 2.700835 | −0.845705 |
| 6 | 1.971188 | 1.133535 | −2.281219 |
| 1 | 2.738547 | −0.024041 | −2.106467 |
| 6 | 2.963366 | −0.355627 | −1.097559 |
| 1 | 3.183147 | −0.755658 | −3.209096 |
| 6 | 3.775613 | −1.654693 | −3.056093 |
| 1 | 2.868144 | −0.33837 | −4.505586 |
| 6 | 3.210561 | −0.910495 | −5.363959 |
| 1 | 2.106317 | 0.817729 | −4.689467 |
| 6 | 1.850037 | 1.14817 | −5.693025 |
| 1 | 1.662081 | 1.545564 | −3.58298 |

| | | | |
|---|---|---|---|
| 8 | 1.0606 | 2.440792 | −3.731482 |
| 6 | −2.419807 | 2.503626 | 0.788138 |
| 1 | −1.261169 | 3.195156 | 1.336077 |
| 1 | −0.361543 | 2.834988 | 0.834752 |
| 6 | −1.183411 | 2.946793 | 2.398945 |
| 6 | −1.45602 | 4.666761 | 1.10771 |
| 1 | −0.844545 | 5.298129 | 0.018454 |
| 6 | −0.215737 | 4.71511 | −0.650473 |
| 1 | −1.032615 | 6.662716 | −0.207752 |
| 6 | −0.548788 | 7.143678 | −1.05371 |
| 1 | −1.839702 | 7.40794 | 0.654802 |
| 6 | −1.985658 | 8.470932 | 0.481923 |
| 1 | −2.455975 | 6.784778 | 1.743642 |
| 6 | −3.081991 | 7.362016 | 2.418897 |
| 1 | −2.262526 | 5.42152 | 1.968042 |
| 6 | −2.738444 | 4.935029 | 2.816232 |
| 6 | −2.30798 | 1.164105 | 0.669925 |
| 6 | −3.665394 | 0.547707 | 0.408547 |
| 6 | −3.659124 | −0.860168 | −0.208071 |
| 6 | −3.706596 | −0.882644 | −1.748107 |
| 6 | −2.406956 | −0.516726 | −2.478527 |
| 1 | −1.814565 | 0.842521 | −2.112718 |
| 1 | −4.25164 | 1.241609 | −0.202821 |
| 1 | −2.792053 | −1.422719 | 0.15755 |
| 1 | −4.513048 | −0.211128 | −2.079635 |
| 1 | −1.641134 | −1.272184 | −2.276268 |
| 1 | −4.141036 | 0.525862 | 1.397379 |
| 1 | −4.545956 | −1.388065 | 0.162879 |
| 1 | −4.002338 | −1.890616 | −2.068428 |
| 1 | −2.595558 | −0.525495 | −3.561567 |
| 1 | −0.977256 | 1.074945 | −2.782203 |
| 8 | −2.558793 | 1.645496 | −2.220345 |
| 8 | −1.460728 | 0.538991 | 1.457272 |
| 1 | −1.318193 | 0.883253 | −0.78432 |
| 6 | 0.125657 | 1.755781 | 6.587994 |
| 1 | 2.823852 | 1.324882 | 2.789793 |
| 6 | 2.818877 | 0.73312 | 1.87346 |
| 1 | 4.133524 | 1.038275 | 3.550036 |
| 1 | 4.199186 | −0.011147 | 3.857431 |
| 1 | 4.999784 | 1.263018 | 2.916059 |
| 6 | 4.213365 | 1.653983 | 4.454074 |
| 1 | 2.756978 | 2.799591 | 2.356029 |

| | | | |
|---|---|---|---|
| 1 | 2.77737 | 3.479093 | 3.21681 |
| 1 | 3.614818 | 3.043678 | 1.71762 |
| 6 | 1.846513 | 2.985474 | 1.781962 |
| 1 | −1.277633 | −1.642373 | 3.679785 |
| 1 | −1.126971 | −1.850096 | 2.617857 |
| 6 | −0.915517 | −2.918056 | 4.464815 |
| 1 | −1.560418 | −3.753355 | 4.16573 |
| 1 | 0.125141 | −3.21333 | 4.292684 |
| 1 | −1.043286 | −2.759958 | 5.542769 |
| 6 | −2.76336 | −1.291953 | 3.86055 |
| 1 | −3.034162 | −1.177206 | 4.916747 |
| 1 | −2.998242 | −0.360912 | 3.338669 |
| 1 | −3.390568 | −2.09109 | 3.447445 |

**Table S36.** Cartesian coordinate of **C$_{ax}$4c.**

| The number of atom coordinates (angstroms) | X | Y | Z |
|---|---|---|---|
| 13 | 0.021267 | 0.328316 | 0.466658 |
| 8 | 0.594477 | 0.889354 | −1.23079 |
| 8 | −2.468802 | 0.818255 | 3.244519 |
| 7 | 1.834991 | −0.336038 | 0.879042 |
| 6 | 1.512951 | 0.355481 | −1.959607 |
| 6 | 1.495968 | 0.738164 | −3.415093 |
| 1 | 0.567959 | 0.376533 | −3.870353 |
| 1 | 2.349871 | 0.333884 | −3.964294 |
| 1 | 1.49447 | 1.830836 | −3.498482 |
| 6 | 2.490076 | −0.495162 | −1.449151 |
| 1 | 3.23324 | −0.885395 | −2.133649 |
| 6 | 2.70101 | −0.710543 | −0.067041 |
| 6 | 4.040916 | −1.305346 | 0.315542 |
| 1 | 4.424977 | −1.931281 | −0.493939 |
| 1 | 4.763901 | −0.497749 | 0.482814 |
| 1 | 3.993173 | −1.889237 | 1.235117 |
| 6 | 2.288585 | −0.349635 | 2.249146 |
| 6 | 1.886616 | −1.399282 | 3.099872 |
| 6 | 2.263198 | −1.356343 | 4.457272 |
| 6 | 3.018123 | −0.273402 | 4.919263 |
| 6 | 3.418287 | 0.746218 | 4.061886 |
| 1 | 4.010305 | 1.567184 | 4.45604 |
| 6 | 3.075493 | 0.728624 | 2.705698 |
| 6 | −3.289689 | −0.136856 | 2.497361 |
| 1 | −3.898522 | −0.626623 | 3.262407 |

| | | | |
|---|---|---|---|
| 1 | −2.641109 | −0.864395 | 2.008872 |
| 6 | −4.12931 | 0.61939 | 1.502138 |
| 6 | −4.038529 | 0.314879 | 0.141428 |
| 1 | −3.343604 | −0.45411 | −0.17728 |
| 6 | −4.799046 | 1.023392 | −0.791911 |
| 1 | −4.713575 | 0.779505 | −1.847644 |
| 6 | −5.651216 | 2.044664 | −0.368784 |
| 1 | −6.238948 | 2.60159 | −1.093811 |
| 6 | −5.746307 | 2.352339 | 0.992643 |
| 1 | −6.408262 | 3.14717 | 1.326118 |
| 6 | −4.992559 | 1.639274 | 1.924321 |
| 1 | −5.059428 | 1.882716 | 2.981824 |
| 8 | −1.047247 | −0.995072 | −0.020672 |
| 6 | −0.802804 | −1.937835 | −1.024298 |
| 1 | 0.254228 | −2.255888 | −1.039331 |
| 1 | −1.383033 | −2.84842 | −0.79578 |
| 6 | −1.177271 | −1.498526 | −2.436258 |
| 6 | −1.892647 | −0.323537 | −2.683305 |
| 1 | −2.150396 | 0.319998 | −1.852081 |
| 6 | −2.237746 | 0.040811 | −3.987231 |
| 1 | −2.787344 | 0.963358 | −4.158852 |
| 6 | −1.865143 | −0.763264 | −5.066728 |
| 1 | −2.12811 | −0.477461 | −6.081879 |
| 6 | −1.140945 | −1.935646 | −4.829901 |
| 1 | −0.835787 | −2.565321 | −5.662149 |
| 6 | −0.802128 | −2.296347 | −3.525159 |
| 1 | −0.231381 | −3.206845 | −3.348741 |
| 6 | −1.158932 | 0.832513 | 3.053672 |
| 6 | −0.47177 | 1.927656 | 3.824456 |
| 6 | −1.175823 | 3.299383 | 3.862696 |
| 6 | −1.01374 | 4.248731 | 2.654853 |
| 6 | −1.785546 | 3.919413 | 1.355296 |
| 6 | −0.985528 | 3.090585 | 0.347311 |
| 1 | 0.54827 | 1.993837 | 3.448247 |
| 1 | −2.242688 | 3.144727 | 4.061879 |
| 1 | 0.053477 | 4.366473 | 2.426124 |
| 1 | −2.710626 | 3.379775 | 1.59552 |
| 1 | −0.402564 | 1.542537 | 4.853483 |
| 1 | −0.776117 | 3.824548 | 4.739971 |
| 1 | −1.351269 | 5.232676 | 3.007672 |
| 1 | −2.084659 | 4.857245 | 0.866032 |
| 1 | −1.600409 | 2.898937 | −0.546881 |

| | | | |
|---|---|---|---|
| 1 | −0.103946 | 3.666259 | 0.01441 |
| 8 | −0.569552 | −0.065523 | 2.43383 |
| 8 | −0.594103 | 1.899718 | 0.966207 |
| 1 | 3.300934 | −0.231425 | 5.968751 |
| 6 | 3.529332 | 1.857606 | 1.781303 |
| 1 | 3.429518 | 1.517876 | 0.746987 |
| 6 | 2.620314 | 3.091814 | 1.920528 |
| 1 | 2.6301 | 3.471005 | 2.950226 |
| 1 | 2.960404 | 3.898096 | 1.259109 |
| 1 | 1.590304 | 2.8414 | 1.654222 |
| 6 | 5.006895 | 2.232608 | 1.990616 |
| 1 | 5.323733 | 2.949464 | 1.224412 |
| 1 | 5.175325 | 2.702949 | 2.96608 |
| 1 | 5.657758 | 1.35281 | 1.927453 |
| 6 | 1.057165 | −2.556031 | 2.571942 |
| 1 | 0.368137 | −2.887643 | 3.355829 |
| 1 | 0.426395 | −2.221577 | 1.746919 |
| 6 | 1.89663 | −3.75795 | 2.101529 |
| 1 | 2.604476 | −4.087914 | 2.870595 |
| 1 | 1.247043 | −4.605138 | 1.852905 |
| 1 | 2.471511 | −3.509195 | 1.2042 |
| 6 | 1.841006 | −2.437531 | 5.427882 |
| 1 | 0.752159 | −2.449574 | 5.568353 |
| 1 | 2.12615 | −3.438056 | 5.08381 |
| 1 | 2.298174 | −2.279861 | 6.409647 |

**Table S37.** Cartesian coordinate of $\mathbf{L_{ax}1a}$.

| The number of atom coordinates (angstroms) | X | Y | Z |
|---|---|---|---|
| 13 | 0.262125 | 1.030969 | −0.416931 |
| 8 | −1.032341 | 2.039628 | −1.308447 |
| 8 | −0.350145 | −0.559267 | −0.926117 |
| 7 | −0.255679 | 1.815554 | 1.321494 |
| 6 | −3.015389 | 3.186476 | −1.88893 |
| 1 | −2.478302 | 3.785743 | −2.632364 |
| 1 | −3.823124 | 3.781062 | −1.455726 |
| 1 | −3.448014 | 2.327067 | −2.413417 |
| 6 | −2.04489 | 2.686684 | −0.851794 |
| 6 | −2.248997 | 2.932945 | 0.500865 |
| 1 | −3.131693 | 3.489384 | 0.788097 |
| 6 | −1.365381 | 2.540574 | 1.525279 |
| 6 | −1.719575 | 2.987037 | 2.928193 |

| | | | |
|---|---|---|---|
| 1 | −0.880479 | 3.507972 | 3.399371 |
| 1 | −2.587533 | 3.648484 | 2.918155 |
| 1 | −1.949487 | 2.121162 | 3.558012 |
| 6 | 0.579641 | 1.56663 | 2.469051 |
| 6 | 1.71567 | 2.352341 | 2.677572 |
| 6 | 2.57484 | 2.072385 | 3.741035 |
| 1 | 3.460689 | 2.683867 | 3.89067 |
| 6 | 2.303814 | 1.007655 | 4.602958 |
| 6 | 1.161276 | 0.228726 | 4.400476 |
| 1 | 0.940809 | −0.600192 | 5.068248 |
| 6 | 0.30091 | 0.507185 | 3.338614 |
| 6 | −1.614886 | −0.803511 | −1.481447 |
| 1 | −1.804478 | −0.177625 | −2.366907 |
| 1 | −1.641277 | −1.850646 | −1.828684 |
| 6 | −2.7551 | −0.594216 | −0.49273 |
| 6 | −2.545658 | −0.752115 | 0.882067 |
| 1 | −1.552224 | −1.013649 | 1.231496 |
| 6 | −3.58192 | −0.543271 | 1.793197 |
| 1 | −3.397394 | −0.661412 | 2.858658 |
| 6 | −4.852069 | −0.178663 | 1.340335 |
| 1 | −5.659136 | −0.009039 | 2.048221 |
| 6 | −5.075962 | −0.030954 | −0.030784 |
| 1 | −6.059516 | 0.256168 | −0.394315 |
| 6 | −4.033766 | −0.236846 | −0.93687 |
| 1 | −4.211883 | −0.106457 | −2.002781 |
| 8 | 1.649085 | 1.757601 | −1.214872 |
| 6 | 2.672773 | 1.106565 | −1.909206 |
| 1 | 2.326753 | 0.168713 | −2.380541 |
| 1 | 2.995212 | 1.751071 | −2.74486 |
| 6 | 3.903139 | 0.791381 | −1.065659 |
| 6 | 4.746645 | −0.274799 | −1.402506 |
| 1 | 4.506057 | −0.901456 | −2.258959 |
| 6 | 5.887829 | −0.553403 | −0.643843 |
| 1 | 6.524809 | −1.391286 | −0.914618 |
| 6 | 6.189913 | 0.228188 | 0.474473 |
| 1 | 7.072876 | 0.011207 | 1.070473 |
| 6 | 5.343736 | 1.285283 | 0.824389 |
| 1 | 5.561539 | 1.88782 | 1.702808 |
| 6 | 4.214139 | 1.566094 | 0.056516 |
| 1 | 3.537534 | 2.365948 | 0.335048 |
| 6 | 2.284318 | −1.128129 | 0.722762 |
| 6 | 2.052931 | −2.281189 | −0.231812 |

| | | | |
|---|---|---|---|
| 6 | 4.202746 | −3.19001 | 0.443524 |
| 6 | 4.102563 | −2.373049 | 1.725067 |
| 1 | 1.764034 | −1.848127 | −1.188654 |
| 1 | 5.090788 | −1.936738 | 1.876155 |
| 8 | 1.593403 | −0.123121 | 0.709489 |
| 8 | 3.225119 | −1.221816 | 1.646101 |
| 1 | 2.976332 | 0.787164 | 5.427357 |
| 8 | 5.126874 | −3.949955 | 0.276712 |
| 8 | 3.249514 | −3.051113 | −0.492954 |
| 6 | 3.711131 | −3.22848 | 2.927013 |
| 1 | 4.450556 | −4.022167 | 3.061217 |
| 1 | 2.725956 | −3.683838 | 2.791996 |
| 1 | 3.686762 | −2.600219 | 3.821375 |
| 6 | 0.92774 | −3.189122 | 0.26574 |
| 1 | 1.144671 | −3.598964 | 1.256588 |
| 1 | 0.810467 | −4.017324 | −0.438236 |
| 1 | 0.005383 | −2.607224 | 0.295464 |
| 1 | 1.923258 | 3.167315 | 1.990913 |
| 1 | −0.581523 | −0.101062 | 3.163562 |

**Table S38.** Cartesian coordinate of **L$_{ax}$TS1a.**

| The number of atom coordinates (angstroms) | X | Y | Z |
|---|---|---|---|
| 13 | 1.228224 | 0.116953 | 1.217193 |
| 8 | 1.592918 | 1.424072 | −0.022912 |
| 8 | −0.091897 | −0.931106 | 0.706947 |
| 7 | 0.311248 | 1.434025 | 2.400665 |
| 6 | 1.460019 | 3.302632 | −1.450211 |
| 1 | 2.551402 | 3.336164 | −1.546763 |
| 1 | 1.059617 | 4.317319 | −1.510291 |
| 1 | 1.072879 | 2.717273 | −2.291226 |
| 6 | 1.089363 | 2.612328 | −0.165811 |
| 6 | 0.273074 | 3.216087 | 0.768907 |
| 1 | −0.128475 | 4.193479 | 0.535378 |
| 6 | −0.113036 | 2.629936 | 2.001332 |
| 6 | −1.070332 | 3.432186 | 2.858024 |
| 1 | −0.558749 | 3.87117 | 3.720735 |
| 1 | −1.518379 | 4.23924 | 2.275282 |
| 1 | −1.865749 | 2.79152 | 3.249208 |
| 6 | −0.110323 | 0.961191 | 3.691139 |
| 6 | 0.425363 | 1.513765 | 4.85884 |
| 6 | 0.033431 | 1.030642 | 6.107771 |

| | | | |
|---|---|---|---|
| 1 | 0.455412 | 1.467555 | 7.008982 |
| 6 | −0.887624 | −0.015304 | 6.199417 |
| 6 | −1.405002 | −0.582274 | 5.031862 |
| 1 | −2.111635 | −1.405978 | 5.092788 |
| 6 | −1.01693 | −0.102025 | 3.780777 |
| 6 | −0.814647 | −0.898506 | −0.501904 |
| 1 | −0.162025 | −1.085309 | −1.364107 |
| 1 | −1.546819 | −1.722315 | −0.479597 |
| 6 | −1.562855 | 0.406882 | −0.722236 |
| 6 | −2.42549 | 0.906769 | 0.26289 |
| 1 | −2.553076 | 0.350207 | 1.186543 |
| 6 | −3.104248 | 2.110044 | 0.0761 |
| 1 | −3.768005 | 2.483166 | 0.852371 |
| 6 | −2.931763 | 2.837198 | −1.106091 |
| 1 | −3.458652 | 3.776712 | −1.252308 |
| 6 | −2.078267 | 2.347869 | −2.095417 |
| 1 | −1.934642 | 2.907087 | −3.016902 |
| 6 | −1.398948 | 1.14139 | −1.901067 |
| 1 | −0.725112 | 0.765655 | −2.667263 |
| 8 | 2.824766 | −0.740918 | 0.746444 |
| 6 | 3.529552 | −0.8307 | −0.485487 |
| 1 | 4.351567 | −1.547078 | −0.354839 |
| 1 | 3.97778 | 0.147282 | −0.700705 |
| 6 | 2.662439 | −1.260053 | −1.651307 |
| 6 | 2.222468 | −2.585671 | −1.766781 |
| 1 | 2.525035 | −3.315917 | −1.021835 |
| 6 | 1.408883 | −2.973331 | −2.832086 |
| 1 | 1.074209 | −4.004602 | −2.908449 |
| 6 | 1.031047 | −2.041659 | −3.803501 |
| 1 | 0.39986 | −2.34443 | −4.634765 |
| 6 | 1.470468 | −0.720156 | −3.700147 |
| 1 | 1.184544 | 0.010361 | −4.453038 |
| 6 | 2.276056 | −0.333687 | −2.626183 |
| 1 | 2.601145 | 0.697961 | −2.533388 |
| 6 | 2.633838 | −1.67762 | 2.428106 |
| 6 | 2.11418 | −3.017495 | 1.945677 |
| 6 | 4.429133 | −3.583469 | 1.48091 |
| 6 | 4.865276 | −2.551451 | 2.519058 |
| 1 | 1.329157 | −2.821448 | 1.216431 |
| 1 | 5.600513 | −1.917254 | 2.015248 |
| 8 | 1.799933 | −0.81017 | 2.823585 |
| 8 | 3.837157 | −1.6559 | 3.001609 |

| | | | |
|---|---|---|---|
| 1 | −1.191393 | −0.392259 | 7.172186 |
| 8 | 5.265147 | −4.221144 | 0.88329 |
| 8 | 3.123649 | −3.780667 | 1.240704 |
| 6 | 5.504421 | −3.237671 | 3.723697 |
| 1 | 6.353928 | −3.839216 | 3.39191 |
| 1 | 4.783319 | −3.88727 | 4.229458 |
| 1 | 5.850753 | −2.480114 | 4.431608 |
| 6 | 1.574481 | −3.824166 | 3.124349 |
| 1 | 2.350772 | −3.997727 | 3.876587 |
| 1 | 1.207802 | −4.789444 | 2.764929 |
| 1 | 0.750383 | −3.276059 | 3.589352 |
| 1 | 1.156315 | 2.313441 | 4.779143 |
| 1 | −1.372539 | −0.563603 | 2.86628 |

**Table S39.** Cartesian coordinate of $\mathbf{L_{ax}2a}$.

| The number of atom coordinates (angstroms) | X | Y | Z |
|---|---|---|---|
| 13 | 0.90318 | −0.224732 | 1.379194 |
| 8 | 1.598102 | 0.94614 | 0.188421 |
| 8 | −0.486247 | −1.189587 | 0.976765 |
| 7 | 0.125974 | 1.183898 | 2.511531 |
| 6 | 1.857036 | 2.826922 | −1.216118 |
| 1 | 2.949961 | 2.740443 | −1.194128 |
| 1 | 1.580974 | 3.878493 | −1.321658 |
| 1 | 1.504363 | 2.269411 | −2.089966 |
| 6 | 1.280943 | 2.203815 | 0.024311 |
| 6 | 0.493312 | 2.926692 | 0.8945 |
| 1 | 0.270545 | 3.953344 | 0.63419 |
| 6 | −0.052394 | 2.433385 | 2.113083 |
| 6 | −0.861223 | 3.404022 | 2.945028 |
| 1 | −0.255718 | 3.824012 | 3.755617 |
| 1 | −1.218585 | 4.229182 | 2.325166 |
| 1 | −1.71723 | 2.903742 | 3.405633 |
| 6 | −0.331738 | 0.767711 | 3.804239 |
| 6 | 0.30172 | 1.23837 | 4.961012 |
| 6 | −0.117302 | 0.798638 | 6.216122 |
| 1 | 0.381263 | 1.167999 | 7.108278 |
| 6 | −1.163744 | −0.120833 | 6.326 |
| 6 | −1.782093 | −0.604029 | 5.170833 |
| 1 | −2.589155 | −1.327841 | 5.24669 |
| 6 | −1.368188 | −0.167879 | 3.911147 |
| 6 | −1.437123 | −1.13856 | −0.058274 |
| 1 | −1.079391 | −1.703986 | −0.931364 |

| | | | |
|---|---|---|---|
| 1 | −2.35468 | −1.635934 | 0.293444 |
| 6 | −1.783701 | 0.272831 | −0.499286 |
| 6 | −2.611968 | 1.081822 | 0.290626 |
| 1 | −3.024745 | 0.679059 | 1.21291 |
| 6 | −2.908615 | 2.39013 | −0.091596 |
| 1 | −3.556066 | 3.003257 | 0.530465 |
| 6 | −2.378794 | 2.909187 | −1.276767 |
| 1 | −2.612329 | 3.926787 | −1.579331 |
| 6 | −1.547236 | 2.114573 | −2.067405 |
| 1 | −1.128451 | 2.511666 | −2.988862 |
| 6 | −1.248875 | 0.806106 | −1.677646 |
| 1 | −0.593099 | 0.192812 | −2.290044 |
| 8 | 2.306124 | −1.645339 | 0.584517 |
| 6 | 3.186111 | −1.4957 | −0.544513 |
| 1 | 3.84317 | −2.371604 | −0.590131 |
| 1 | 3.794339 | −0.59538 | −0.423982 |
| 6 | 2.345499 | −1.425317 | −1.793815 |
| 6 | 1.377197 | −2.40543 | −2.042635 |
| 1 | 1.214095 | −3.187901 | −1.307495 |
| 6 | 0.615315 | −2.364322 | −3.210558 |
| 1 | −0.136987 | −3.127225 | −3.391201 |
| 6 | 0.817853 | −1.344496 | −4.145339 |
| 1 | 0.22438 | −1.312637 | −5.054949 |
| 6 | 1.781553 | −0.363446 | −3.90114 |
| 1 | 1.940871 | 0.436391 | −4.619501 |
| 6 | 2.537692 | −0.403445 | −2.727434 |
| 1 | 3.275115 | 0.369384 | −2.530084 |
| 6 | 2.823514 | −1.730164 | 1.979264 |
| 6 | 2.703117 | −3.186101 | 2.435902 |
| 6 | 4.912186 | −3.584285 | 1.531622 |
| 6 | 5.239124 | −2.110406 | 1.782182 |
| 1 | 1.731129 | −3.54832 | 2.098615 |
| 1 | 5.6875 | −1.764781 | 0.843079 |
| 8 | 1.980731 | −0.923205 | 2.645064 |
| 8 | 4.13019 | −1.245004 | 2.075415 |
| 1 | −1.487639 | −0.464934 | 7.304335 |
| 8 | 5.766428 | −4.331118 | 1.108302 |
| 8 | 3.662292 | −4.030431 | 1.73894 |
| 6 | 6.276077 | −1.966823 | 2.896556 |
| 1 | 7.171161 | −2.540144 | 2.642197 |
| 1 | 5.881678 | −2.326238 | 3.851207 |
| 1 | 6.54015 | −0.911517 | 3.00637 |

| | | | |
|---|---|---|---|
| 6 | 2.845999 | −3.349077 | 3.941448 |
| 1 | 3.79778 | −2.942194 | 4.294909 |
| 1 | 2.794902 | −4.409553 | 4.203953 |
| 1 | 2.037444 | −2.810403 | 4.440722 |
| 1 | 1.13 | 1.934188 | 4.864792 |
| 1 | −1.815778 | −0.559983 | 3.005281 |

**Table S40.** Cartesian coordinate of $\mathbf{L_{ax}3a}$.

| The number of atom coordinates (angstroms) | X | Y | Z |
|---|---|---|---|
| 13 | 4.652025 | −2.240079 | −3.858678 |
| 8 | 6.401227 | −2.693652 | −3.776159 |
| 8 | 3.860932 | −2.576011 | −5.372491 |
| 7 | 5.011865 | −0.338823 | −4.138592 |
| 6 | 8.63659 | −2.858906 | −4.513082 |
| 1 | 8.964982 | −3.207455 | −3.526975 |
| 1 | 9.444251 | −2.293693 | −4.983804 |
| 1 | 8.417081 | −3.750979 | −5.111739 |
| 6 | 7.3758 | −2.054088 | −4.364708 |
| 6 | 7.285951 | −0.75077 | −4.815276 |
| 1 | 8.162602 | −0.318706 | −5.28185 |
| 6 | 6.155681 | 0.101641 | −4.639484 |
| 6 | 6.313997 | 1.549185 | −5.048388 |
| 1 | 5.403154 | 1.925167 | −5.522164 |
| 1 | 6.506456 | 2.178473 | −4.172488 |
| 1 | 7.153928 | 1.664174 | −5.736925 |
| 6 | 3.928631 | 0.567766 | −3.890273 |
| 6 | 4.008811 | 1.504395 | −2.853623 |
| 6 | 2.931517 | 2.351761 | −2.595721 |
| 1 | 2.999159 | 3.075727 | −1.78819 |
| 6 | 1.768161 | 2.263339 | −3.364159 |
| 6 | 1.685172 | 1.316734 | −4.387863 |
| 1 | 0.779905 | 1.236071 | −4.983396 |
| 6 | 2.758788 | 0.465392 | −4.652505 |
| 6 | 3.366785 | −3.792841 | −5.868913 |
| 1 | 2.721418 | −4.293068 | −5.13533 |
| 1 | 2.736777 | −3.573728 | −6.746623 |
| 6 | 4.460853 | −4.763981 | −6.285202 |
| 6 | 5.699911 | −4.295721 | −6.739076 |
| 1 | 5.873282 | −3.224931 | −6.784745 |
| 6 | 6.707868 | −5.190787 | −7.103767 |
| 1 | 7.665982 | −4.812092 | −7.452229 |

| | | | |
|---|---|---|---|
| 6 | 6.489348 | −6.569103 | −7.022332 |
| 1 | 7.276688 | −7.265744 | −7.297788 |
| 6 | 5.25265 | −7.044039 | −6.579425 |
| 1 | 5.075037 | −8.113793 | −6.502795 |
| 6 | 4.247071 | −6.146193 | −6.214021 |
| 1 | 3.295095 | −6.522395 | −5.846326 |
| 8 | 2.125282 | −3.553631 | −1.936969 |
| 6 | 1.442862 | −4.684906 | −1.37067 |
| 1 | 0.439463 | −4.29737 | −1.164604 |
| 1 | 1.878958 | −4.98962 | −0.415248 |
| 6 | 1.35351 | −5.857603 | −2.322977 |
| 6 | 0.943145 | −5.641766 | −3.645343 |
| 1 | 0.707584 | −4.631294 | −3.966022 |
| 6 | 0.853944 | −6.705692 | −4.541309 |
| 1 | 0.538909 | −6.525189 | −5.565758 |
| 6 | 1.173641 | −8.002688 | −4.12359 |
| 1 | 1.111533 | −8.831446 | −4.823673 |
| 6 | 1.571116 | −8.226589 | −2.804869 |
| 1 | 1.826571 | −9.228935 | −2.47301 |
| 6 | 1.655594 | −7.1579 | −1.90752 |
| 1 | 1.988045 | −7.336042 | −0.88875 |
| 6 | 3.51504 | −3.390241 | −1.893611 |
| 6 | 4.158411 | −3.706199 | −0.52899 |
| 6 | 4.555327 | −6.010162 | −1.244911 |
| 6 | 4.644937 | −5.546508 | −2.695006 |
| 1 | 3.496097 | −3.323899 | 0.25111 |
| 1 | 3.974579 | −6.205981 | −3.254339 |
| 8 | 3.777407 | −2.129449 | −2.300117 |
| 8 | 4.199122 | −4.194307 | −2.918816 |
| 1 | 0.928632 | 2.921988 | −3.160004 |
| 8 | 4.764078 | −7.167808 | −0.95919 |
| 8 | 4.205432 | −5.14017 | −0.276475 |
| 6 | 6.074841 | −5.725383 | −3.204464 |
| 1 | 6.346366 | −6.779521 | −3.108405 |
| 1 | 6.776684 | −5.114372 | −2.632891 |
| 1 | 6.132602 | −5.429609 | −4.250878 |
| 6 | 5.553364 | −3.117805 | −0.374038 |
| 1 | 6.195645 | −3.401944 | −1.212062 |
| 1 | 5.994845 | −3.479218 | 0.559131 |
| 1 | 5.493959 | −2.028029 | −0.348064 |
| 1 | 2.704705 | −0.289151 | −5.430646 |

| | | | |
|---|---|---|---|
| 1 | 4.909676 | 1.55182 | −2.248645 |

**Table S41.** Cartesian coordinate of $\mathbf{L_{ax}TS2a}$.

| The number of atom coordinates (angstroms) | X | Y | Z |
|---|---|---|---|
| 13 | 4.821561 | −3.426514 | −3.975671 |
| 8 | 6.630763 | −3.840977 | −4.10109 |
| 8 | 4.251197 | −4.126542 | −5.473376 |
| 7 | 5.1949 | −1.51566 | −4.308736 |
| 6 | 8.933784 | −3.892014 | −4.622352 |
| 1 | 9.210634 | −4.290115 | −3.638708 |
| 1 | 9.748715 | −3.267911 | −4.996403 |
| 1 | 8.798657 | −4.750444 | −5.290011 |
| 6 | 7.633435 | −3.141211 | −4.509759 |
| 6 | 7.547692 | −1.792485 | −4.822679 |
| 1 | 8.44286 | −1.29304 | −5.170872 |
| 6 | 6.364391 | −1.024925 | −4.733673 |
| 6 | 6.464887 | 0.422991 | −5.167432 |
| 1 | 6.330592 | 1.099149 | −4.317197 |
| 1 | 7.437326 | 0.625094 | −5.619948 |
| 1 | 5.678392 | 0.664426 | −5.889199 |
| 6 | 4.07114 | −0.614834 | −4.290673 |
| 6 | 3.922257 | 0.317896 | −3.260012 |
| 6 | 2.814325 | 1.16625 | −3.239131 |
| 1 | 2.705799 | 1.889368 | −2.435099 |
| 6 | 1.848816 | 1.084195 | −4.245374 |
| 6 | 1.996342 | 0.146435 | −5.270457 |
| 1 | 1.246004 | 0.071835 | −6.052939 |
| 6 | 3.102281 | −0.703836 | −5.294617 |
| 6 | 3.224545 | −5.063708 | −5.649323 |
| 1 | 2.270075 | −4.697593 | −5.239749 |
| 1 | 3.06625 | −5.204543 | −6.731825 |
| 6 | 3.512863 | −6.425364 | −5.027034 |
| 6 | 4.822925 | −6.842081 | −4.762661 |
| 1 | 5.645889 | −6.173667 | −4.994849 |
| 6 | 5.069886 | −8.086276 | −4.176909 |
| 1 | 6.093521 | −8.390719 | −3.970773 |
| 6 | 4.009437 | −8.936973 | −3.852484 |
| 1 | 4.20142 | −9.90161 | −3.38968 |
| 6 | 2.699907 | −8.5335 | −4.125376 |
| 1 | 1.86214 | −9.176946 | −3.869712 |
| 6 | 2.457642 | −7.287973 | −4.708067 |

| | | | |
|---|---|---|---|
| 1 | 1.433517 | −6.972781 | −4.891052 |
| 8 | 1.945157 | −4.691477 | −2.648701 |
| 6 | 1.468441 | −5.741401 | −1.774104 |
| 1 | 1.256188 | −5.337899 | −0.780168 |
| 1 | 2.258411 | −6.491658 | −1.683011 |
| 6 | 0.227454 | −6.339604 | −2.387581 |
| 6 | −0.632677 | −5.581791 | −3.188836 |
| 1 | −0.386274 | −4.548472 | −3.410968 |
| 6 | −1.785638 | −6.160185 | −3.723551 |
| 1 | −2.441725 | −5.566158 | −4.354039 |
| 6 | −2.094606 | −7.494872 | −3.452933 |
| 1 | −2.990961 | −7.943852 | −3.871814 |
| 6 | −1.241852 | −8.251925 | −2.645837 |
| 1 | −1.470512 | −9.293076 | −2.435207 |
| 6 | −0.084197 | −7.67739 | −2.12025 |
| 1 | 0.588648 | −8.275242 | −1.510231 |
| 6 | 2.789228 | −3.729008 | −2.290904 |
| 6 | 2.996993 | −3.413303 | −0.806329 |
| 6 | 4.462992 | −5.385639 | −0.251672 |
| 6 | 5.252724 | −5.331885 | −1.565937 |
| 1 | 2.004896 | −3.178601 | −0.398178 |
| 1 | 5.205435 | −6.358993 | −1.958229 |
| 8 | 3.053933 | −2.880984 | −3.18486 |
| 8 | 4.64706 | −4.454242 | −2.461321 |
| 1 | 0.98569 | 1.743841 | −4.228362 |
| 8 | 4.7325 | −6.193161 | 0.608 |
| 8 | 3.394481 | −4.56464 | −0.025564 |
| 6 | 6.718521 | −4.997283 | −1.257281 |
| 1 | 7.098047 | −5.675427 | −0.48842 |
| 1 | 6.809958 | −3.965046 | −0.901312 |
| 1 | 7.307535 | −5.101172 | −2.168598 |
| 6 | 3.917336 | −2.227916 | −0.56847 |
| 1 | 4.914245 | −2.428289 | −0.965357 |
| 1 | 3.987512 | −2.040603 | 0.506453 |
| 1 | 3.516684 | −1.342084 | −1.066093 |
| 1 | 4.675696 | 0.369836 | −2.478994 |
| 1 | 3.218258 | −1.456606 | −6.068137 |

**Table 42.** Cartesian coordinate of **L$_{ax}$4a.**

| The number of atom coordinates (angstroms) | X | Y | Z |
|---|---|---|---|
| 13 | 4.855023 | −3.511808 | −4.003204 |

| | | | |
|---|---|---|---|
| 8 | 6.330985 | −4.555138 | −3.995386 |
| 8 | 3.913935 | −3.690444 | −5.427362 |
| 7 | 5.777421 | −1.82755 | −4.184879 |
| 6 | 8.50742 | −5.327727 | −4.492763 |
| 1 | 8.581035 | −5.831665 | −3.522148 |
| 1 | 9.499639 | −4.992847 | −4.802649 |
| 1 | 8.134809 | −6.068549 | −5.209885 |
| 6 | 7.522313 | −4.197987 | −4.39102 |
| 6 | 7.881746 | −2.89446 | −4.690805 |
| 1 | 8.89817 | −2.723661 | −5.023545 |
| 6 | 7.052104 | −1.746126 | −4.555469 |
| 6 | 7.696607 | −0.411229 | −4.854951 |
| 1 | 8.167591 | 0.002155 | −3.955828 |
| 1 | 8.479489 | −0.537869 | −5.606836 |
| 1 | 6.967332 | 0.318296 | −5.212973 |
| 6 | 4.999798 | −0.657901 | −3.895867 |
| 6 | 5.388152 | 0.232297 | −2.8844 |
| 6 | 4.578338 | 1.321207 | −2.565697 |
| 1 | 4.885253 | 2.005822 | −1.779921 |
| 6 | 3.371383 | 1.520579 | −3.241529 |
| 6 | 2.977229 | 0.62091 | −4.233454 |
| 1 | 2.031699 | 0.756283 | −4.750024 |
| 6 | 3.783741 | −0.468414 | −4.56247 |
| 6 | 2.738749 | −4.400748 | −5.748309 |
| 1 | 1.890097 | −4.006981 | −5.177242 |
| 1 | 2.524857 | −4.218697 | −6.813645 |
| 6 | 2.836105 | −5.900108 | −5.526249 |
| 6 | 4.059945 | −6.57695 | −5.596508 |
| 1 | 4.96823 | −6.014598 | −5.787889 |
| 6 | 4.118504 | −7.960045 | −5.40837 |
| 1 | 5.077799 | −8.470037 | −5.458111 |
| 6 | 2.952104 | −8.686457 | −5.157242 |
| 1 | 2.998582 | −9.761753 | −5.003949 |
| 6 | 1.726377 | −8.019465 | −5.092117 |
| 1 | 0.814611 | −8.565171 | −4.867693 |
| 6 | 1.673892 | −6.637437 | −5.272634 |
| 1 | 0.721902 | −6.121756 | −5.187078 |
| 8 | 1.275933 | −4.546679 | −2.622428 |
| 6 | 1.308368 | −5.581818 | −1.618544 |
| 1 | 0.960958 | −5.189076 | −0.659058 |
| 1 | 2.342144 | −5.916859 | −1.492749 |
| 6 | 0.425134 | −6.721659 | −2.061704 |

| | | | |
|---|---|---|---|
| 6 | −0.759712 | −6.490981 | −2.769018 |
| 1 | −1.019358 | −5.476363 | −3.054924 |
| 6 | −1.582932 | −7.557722 | −3.131393 |
| 1 | −2.494728 | −7.368311 | −3.691669 |
| 6 | −1.238881 | −8.864836 | −2.774957 |
| 1 | −1.881783 | −9.694715 | −3.055763 |
| 6 | −0.062197 | −9.098222 | −2.060053 |
| 1 | 0.218533 | 10.111644 | −1.786143 |
| 6 | 0.767937 | −8.031433 | −1.71236 |
| 1 | 1.694725 | −8.22015 | −1.17571 |
| 6 | 1.626383 | −3.261192 | −2.415068 |
| 6 | 1.898061 | −2.720123 | −1.001028 |
| 6 | 3.827538 | −4.051978 | −0.036687 |
| 6 | 4.768857 | −3.962122 | −1.243636 |
| 1 | 0.902852 | −2.622244 | −0.546789 |
| 1 | 5.202875 | −4.971577 | −1.297981 |
| 8 | 1.535095 | −2.48877 | −3.346161 |
| 8 | 4.095194 | −3.654413 | −2.433786 |
| 1 | 2.73775 | 2.365407 | −2.986932 |
| 8 | 4.212507 | −4.573968 | 0.987522 |
| 8 | 2.544048 | −3.611665 | −0.062496 |
| 6 | 5.909077 | −2.983676 | −0.903956 |
| 1 | 6.359686 | −3.251472 | 0.055659 |
| 1 | 5.535411 | −1.956995 | −0.850484 |
| 1 | 6.680234 | −3.029956 | −1.678737 |
| 6 | 2.555707 | −1.348697 | −1.036798 |
| 1 | 3.49301 | −1.386825 | −1.588877 |
| 1 | 2.735341 | −1.009035 | −0.012456 |
| 1 | 1.904606 | −0.635327 | −1.544016 |
| 1 | 3.47489 | −1.1864 | −5.314513 |
| 1 | 6.310846 | 0.055059 | −2.340877 |

**Table S43.** Cartesian coordinate of **$L_{ax}$5a.**

| The number of atom coordinates (angstroms) | X | Y | Z |
|---|---|---|---|
| 13 | 0.856223 | −0.394274 | 1.391754 |
| 8 | 1.069247 | 0.473149 | −0.190581 |
| 8 | −0.703501 | −1.164894 | 1.661408 |
| 7 | 0.388819 | 1.269664 | 2.365355 |
| 6 | 0.670507 | 1.931346 | −2.002587 |
| 1 | 1.702656 | 1.79734 | −2.343042 |
| 1 | 0.33161 | 2.940941 | −2.247354 |

| | | | |
|---|---|---|---|
| 1 | 0.056679 | 1.201441 | −2.543253 |
| 6 | 0.590282 | 1.635348 | −0.529941 |
| 6 | 0.044143 | 2.545784 | 0.355878 |
| 1 | −0.322245 | 3.481927 | −0.047227 |
| 6 | −0.003004 | 2.382905 | 1.771473 |
| 6 | −0.519842 | 3.550324 | 2.585166 |
| 1 | −1.200483 | 3.20699 | 3.369808 |
| 1 | 0.30631 | 4.069228 | 3.083218 |
| 1 | −1.03887 | 4.269263 | 1.947684 |
| 6 | 0.392685 | 1.186585 | 3.79603 |
| 6 | 1.38026 | 1.840266 | 4.541708 |
| 6 | 1.398207 | 1.722713 | 5.931348 |
| 1 | 2.169408 | 2.231801 | 6.503472 |
| 6 | 0.436178 | 0.947593 | 6.584355 |
| 6 | −0.539678 | 0.284205 | 5.836926 |
| 1 | −1.286298 | −0.327499 | 6.33673 |
| 6 | −0.563706 | 0.397105 | 4.445697 |
| 6 | −1.262364 | −2.262883 | 0.994606 |
| 1 | −0.622168 | −3.157664 | 1.061834 |
| 1 | −2.208443 | −2.524902 | 1.497823 |
| 6 | −1.56378 | −2.000785 | −0.475272 |
| 6 | −1.949367 | −0.725632 | −0.905277 |
| 1 | −1.993453 | 0.082422 | −0.181431 |
| 6 | −2.25465 | −0.487722 | −2.246639 |
| 1 | −2.552648 | 0.509131 | −2.563007 |
| 6 | −2.181183 | −1.525167 | −3.181034 |
| 1 | −2.414448 | −1.339857 | −4.226222 |
| 6 | −1.795395 | −2.800617 | −2.76159 |
| 1 | −1.720178 | −3.611426 | −3.481758 |
| 6 | −1.486516 | −3.031537 | −1.418908 |
| 1 | −1.173459 | −4.024251 | −1.100541 |
| 8 | 5.31498 | −4.207034 | −1.557179 |
| 6 | 4.02737 | −4.406635 | −2.212801 |
| 1 | 3.312836 | −4.89546 | −1.551239 |
| 1 | 4.277415 | −5.099012 | −3.021599 |
| 6 | 3.483732 | −3.108289 | −2.754208 |
| 6 | 2.146713 | −2.754954 | −2.553132 |
| 1 | 1.498155 | −3.386911 | −1.956114 |
| 6 | 1.641168 | −1.566472 | −3.084117 |
| 1 | 0.606747 | −1.299919 | −2.896366 |
| 6 | 2.473188 | −0.726607 | −3.824069 |
| 1 | 2.082176 | 0.198854 | −4.237859 |

| | | | |
|---|---|---|---|
| 6 | 3.813924 | −1.071211 | −4.025544 |
| 1 | 4.46618 | −0.415457 | −4.596305 |
| 6 | 4.316985 | −2.257407 | −3.494319 |
| 1 | 5.36018 | −2.524328 | −3.64011 |
| 6 | 5.516599 | −3.94275 | −0.258487 |
| 6 | 4.416219 | −4.138126 | 0.801766 |
| 6 | 2.57485 | −2.531415 | 0.927389 |
| 6 | 3.036837 | −1.862764 | 2.229744 |
| 1 | 4.780569 | −3.602325 | 1.675392 |
| 1 | 4.116062 | −1.65175 | 2.159515 |
| 8 | 6.629935 | −3.656014 | 0.123829 |
| 8 | 2.319731 | −0.689742 | 2.369157 |
| 1 | 0.452413 | 0.8555 | 7.666955 |
| 8 | 1.5643 | −2.088667 | 0.384703 |
| 8 | 3.14352 | −3.596426 | 0.369983 |
| 6 | 2.771108 | −2.809892 | 3.419778 |
| 1 | 3.365699 | −3.728995 | 3.391563 |
| 1 | 1.708474 | −3.072056 | 3.447959 |
| 1 | 3.010988 | −2.264122 | 4.335592 |
| 6 | 4.220427 | −5.615248 | 1.129579 |
| 1 | 3.861277 | −6.17128 | 0.259672 |
| 1 | 3.49105 | −5.720247 | 1.937897 |
| 1 | 5.172489 | −6.045843 | 1.453004 |
| 1 | 2.135826 | 2.422783 | 4.022891 |
| 1 | −1.294924 | −0.132663 | 3.844793 |

**Table S44.** Cartesian coordinate of **L$_{ax}$1b.**

| The number of atom coordinates (angstroms) | X | Y | Z |
|---|---|---|---|
| 13 | 0.244316 | 1.017588 | −0.448719 |
| 8 | −1.073228 | 2.030764 | −1.300211 |
| 8 | −0.372784 | −0.575849 | −0.938497 |
| 7 | −0.251126 | 1.787046 | 1.312791 |
| 6 | −3.027556 | 3.242254 | −1.842734 |
| 1 | −2.4869 | 3.826515 | −2.595448 |
| 1 | −3.808612 | 3.860124 | −1.393566 |
| 1 | −3.495057 | 2.3968 | −2.359523 |
| 6 | −2.052447 | 2.712698 | −0.82505 |
| 6 | −2.221353 | 2.968599 | 0.532007 |
| 1 | −3.079654 | 3.553964 | 0.835216 |
| 6 | −1.3398 | 2.537376 | 1.540391 |
| 6 | −1.667344 | 2.96516 | 2.95528 |

| | | | |
|---|---|---|---|
| 1 | −0.80446 | 3.437513 | 3.434913 |
| 1 | −2.505668 | 3.663713 | 2.966765 |
| 1 | −1.933934 | 2.09514 | 3.565114 |
| 6 | 0.572272 | 1.462268 | 2.445779 |
| 6 | 1.756414 | 2.169397 | 2.667859 |
| 6 | 2.640314 | 1.77569 | 3.670915 |
| 1 | 3.572894 | 2.307146 | 3.826021 |
| 6 | 2.322585 | 0.669629 | 4.45595 |
| 6 | 1.129813 | −0.029015 | 4.275281 |
| 1 | 0.896116 | −0.883098 | 4.902098 |
| 6 | 0.256142 | 0.375656 | 3.267774 |
| 6 | −1.636983 | −0.814156 | −1.500558 |
| 1 | −1.814592 | −0.195262 | −2.393231 |
| 1 | −1.669928 | −1.864307 | −1.837364 |
| 6 | −2.781931 | −0.584794 | −0.522165 |
| 6 | −2.590997 | −0.766136 | 0.852483 |
| 1 | −1.609267 | −1.063912 | 1.206087 |
| 6 | −3.629838 | −0.540544 | 1.756503 |
| 1 | −3.460683 | −0.679974 | 2.822 |
| 6 | −4.884669 | −0.133781 | 1.296231 |
| 1 | −5.693665 | 0.049082 | 1.99854 |
| 6 | −5.090525 | 0.037764 | −0.074744 |
| 1 | −6.061955 | 0.357012 | −0.443946 |
| 6 | −4.045601 | −0.186434 | −0.973644 |
| 1 | −4.209597 | −0.038661 | −2.039464 |
| 8 | 1.600452 | 1.762336 | −1.285807 |
| 6 | 2.627763 | 1.108019 | −1.974614 |
| 1 | 2.288381 | 0.156406 | −2.422213 |
| 1 | 2.935504 | 1.739185 | −2.825639 |
| 6 | 3.867128 | 0.8245 | −1.133265 |
| 6 | 4.688244 | −0.274661 | −1.41384 |
| 1 | 4.427838 | −0.946757 | −2.229136 |
| 6 | 5.832255 | −0.529289 | −0.65036 |
| 1 | 6.451091 | −1.393609 | −0.876584 |
| 6 | 6.161232 | 0.312121 | 0.415164 |
| 1 | 7.046077 | 0.11459 | 1.014887 |
| 6 | 5.340225 | 1.406322 | 0.706956 |
| 1 | 5.583494 | 2.059275 | 1.541639 |
| 6 | 4.206636 | 1.660684 | −0.064278 |
| 1 | 3.55114 | 2.493023 | 0.168167 |
| 6 | 2.319498 | −1.083458 | 0.724526 |
| 6 | 2.124008 | −2.293232 | −0.163068 |

| | | | |
|---|---|---|---|
| 6 | 4.25689 | −3.141358 | 0.636222 |
| 6 | 4.120627 | −2.236587 | 1.854179 |
| 1 | 1.873113 | −1.917513 | −1.155009 |
| 1 | 5.103238 | −1.787102 | 2.002609 |
| 8 | 1.606303 | −0.098702 | 0.634892 |
| 8 | 3.243144 | −1.09722 | 1.666833 |
| 8 | 5.188393 | −3.904735 | 0.545685 |
| 8 | 3.326288 | −3.077258 | −0.332152 |
| 6 | 3.692818 | −3.002271 | 3.103264 |
| 1 | 4.413805 | −3.799528 | 3.30168 |
| 1 | 2.700811 | −3.44615 | 2.977986 |
| 1 | 3.667395 | −2.31646 | 3.954349 |
| 6 | 0.976532 | −3.164606 | 0.348741 |
| 1 | 1.154535 | −3.50812 | 1.372253 |
| 1 | 0.882577 | −4.037357 | −0.302856 |
| 1 | 0.057585 | −2.577437 | 0.303387 |
| 1 | 2.000768 | 3.004854 | 2.020195 |
| 1 | −0.664225 | −0.17191 | 3.092562 |
| 17 | 3.463123 | 0.123948 | 5.688353 |

**Table S45.** Cartesian coordinate of **L$_{ax}$TS1b.**

| The number of atom coordinates (angstroms) | X | Y | Z |
|---|---|---|---|
| 13 | 1.236028 | 0.121135 | 1.210665 |
| 8 | 1.599192 | 1.430698 | −0.026308 |
| 8 | −0.090429 | −0.924789 | 0.713758 |
| 7 | 0.315159 | 1.440087 | 2.393973 |
| 6 | 1.47164 | 3.310831 | −1.45119 |
| 1 | 2.563175 | 3.342423 | −1.546063 |
| 1 | 1.072978 | 4.326161 | −1.510738 |
| 1 | 1.084891 | 2.726796 | −2.293301 |
| 6 | 1.098367 | 2.619652 | −0.168308 |
| 6 | 0.281542 | 3.22445 | 0.766619 |
| 1 | −0.118112 | 4.202523 | 0.533025 |
| 6 | −0.108143 | 2.63799 | 1.996308 |
| 6 | −1.066929 | 3.438288 | 2.852787 |
| 1 | −0.557976 | 3.874374 | 3.718677 |
| 1 | −1.51187 | 4.248142 | 2.271809 |
| 1 | −1.865595 | 2.798118 | 3.238336 |
| 6 | −0.108288 | 0.961189 | 3.678093 |
| 6 | 0.416615 | 1.509241 | 4.852585 |
| 6 | 0.033955 | 1.017765 | 6.099925 |

| | | | |
|---|---|---|---|
| 1 | 0.441594 | 1.442798 | 7.010809 |
| 6 | −0.872892 | −0.038905 | 6.161769 |
| 6 | −1.390707 | −0.614596 | 5.001956 |
| 1 | −2.084475 | −1.446 | 5.06819 |
| 6 | −1.001623 | −0.11388 | 3.760823 |
| 6 | −0.823585 | −0.896955 | −0.489784 |
| 1 | −0.178431 | −1.09325 | −1.355344 |
| 1 | −1.559521 | −1.71666 | −0.455046 |
| 6 | −1.565821 | 0.411365 | −0.711815 |
| 6 | −2.433018 | 0.911971 | 0.269018 |
| 1 | −2.571962 | 0.35177 | 1.189004 |
| 6 | −3.104897 | 2.119083 | 0.082004 |
| 1 | −3.77303 | 2.49215 | 0.854496 |
| 6 | −2.92088 | 2.849371 | −1.096544 |
| 1 | −3.442774 | 3.791538 | −1.243286 |
| 6 | −2.063277 | 2.359246 | −2.081856 |
| 1 | −1.911457 | 2.920503 | −3.000738 |
| 6 | −1.390804 | 1.148986 | −1.887079 |
| 1 | −0.714212 | 0.772294 | −2.650265 |
| 8 | 2.827731 | −0.740122 | 0.738452 |
| 6 | 3.526734 | −0.841132 | −0.496479 |
| 1 | 4.345363 | −1.560952 | −0.364996 |
| 1 | 3.978958 | 0.133276 | −0.719173 |
| 6 | 2.650891 | −1.272763 | −1.654567 |
| 6 | 2.206608 | −2.597697 | −1.761193 |
| 1 | 2.5125 | −3.325488 | −1.015138 |
| 6 | 1.385055 | −2.98773 | −2.819466 |
| 1 | 1.04727 | −4.018416 | −2.889324 |
| 6 | 1.003522 | −2.059176 | −3.792456 |
| 1 | 0.366417 | −2.363895 | −4.61844 |
| 6 | 1.446946 | −0.73837 | −3.6977 |
| 1 | 1.158295 | −0.010535 | −4.452117 |
| 6 | 2.260438 | −0.349316 | −2.630619 |
| 1 | 2.589266 | 0.68183 | −2.544667 |
| 6 | 2.645895 | −1.664347 | 2.425645 |
| 6 | 2.124142 | −3.007877 | 1.955724 |
| 6 | 4.437113 | −3.577233 | 1.486285 |
| 6 | 4.876741 | −2.539955 | 2.517673 |
| 1 | 1.335223 | −2.817662 | 1.229131 |
| 1 | 5.613408 | −1.910874 | 2.009706 |
| 8 | 1.812491 | −0.794106 | 2.819923 |
| 8 | 3.850801 | −1.637544 | 2.993831 |

| | | | |
|---|---|---|---|
| 8 | 5.270807 | −4.218743 | 0.889882 |
| 8 | 3.13051 | −3.774297 | 1.250429 |
| 6 | 5.514147 | −3.219675 | 3.726854 |
| 1 | 6.36249 | −3.82491 | 3.398794 |
| 1 | 4.792079 | −3.864803 | 4.236842 |
| 1 | 5.862132 | −2.458331 | 4.429851 |
| 6 | 1.590794 | −3.806661 | 3.142713 |
| 1 | 2.369907 | −3.973313 | 3.893449 |
| 1 | 1.224449 | −4.775168 | 2.791861 |
| 1 | 0.767357 | −3.257122 | 3.607194 |
| 1 | −1.349982 | −0.577227 | 2.844701 |
| 1 | 1.139401 | 2.316851 | 4.786897 |
| 17 | −1.362419 | −0.669133 | 7.735668 |

**Table S46.** Cartesian coordinate of **L$_{ax}$2b.**

| The number of atom coordinates (angstroms) | X | Y | Z |
|---|---|---|---|
| 13 | 0.907363 | −0.209679 | 1.36681 |
| 8 | 1.595919 | 0.963965 | 0.175165 |
| 8 | −0.510319 | −1.136947 | 0.958682 |
| 7 | 0.142464 | 1.204136 | 2.511575 |
| 6 | 1.817536 | 2.840881 | −1.243924 |
| 1 | 2.910404 | 2.752684 | −1.243913 |
| 1 | 1.54083 | 3.892866 | −1.342518 |
| 1 | 1.445136 | 2.28649 | −2.111563 |
| 6 | 1.264062 | 2.217596 | 0.006563 |
| 6 | 0.47772 | 2.93841 | 0.880153 |
| 1 | 0.241293 | 3.960529 | 0.614229 |
| 6 | −0.05122 | 2.450314 | 2.107102 |
| 6 | −0.860822 | 3.423281 | 2.935683 |
| 1 | −0.235966 | 3.90605 | 3.69541 |
| 1 | −1.277033 | 4.204679 | 2.295903 |
| 1 | −1.676225 | 2.915551 | 3.456864 |
| 6 | −0.290239 | 0.788759 | 3.809122 |
| 6 | 0.3145 | 1.30774 | 4.960581 |
| 6 | −0.070331 | 0.865868 | 6.224525 |
| 1 | 0.399356 | 1.265794 | 7.116668 |
| 6 | −1.060241 | −0.110911 | 6.3287 |
| 6 | −1.660292 | −0.654358 | 5.193814 |
| 1 | −2.420524 | −1.421705 | 5.293016 |
| 6 | −1.269978 | −0.204619 | 3.933042 |
| 6 | −1.352936 | −1.093059 | −0.169458 |

| | | | |
|---|---|---|---|
| 1 | −0.872723 | −1.57541 | −1.032687 |
| 1 | −2.258561 | −1.675426 | 0.061079 |
| 6 | −1.760287 | 0.317943 | −0.556231 |
| 6 | −2.579472 | 1.073967 | 0.293948 |
| 1 | −2.934943 | 0.62959 | 1.220744 |
| 6 | −2.933512 | 2.382379 | −0.032803 |
| 1 | −3.572507 | 2.954334 | 0.63554 |
| 6 | −2.470476 | 2.956483 | −1.221249 |
| 1 | −2.747537 | 3.975434 | −1.479179 |
| 6 | −1.650194 | 2.214665 | −2.071984 |
| 1 | −1.282627 | 2.655075 | −2.995646 |
| 6 | −1.295101 | 0.904182 | −1.738144 |
| 1 | −0.646325 | 0.331996 | −2.396937 |
| 8 | 2.291101 | −1.636892 | 0.587789 |
| 6 | 3.189598 | −1.522436 | −0.533882 |
| 1 | 3.832829 | −2.408672 | −0.550905 |
| 1 | 3.808821 | −0.62882 | −0.420425 |
| 6 | 2.366964 | −1.465943 | −1.794527 |
| 6 | 1.469273 | −2.499724 | −2.088114 |
| 1 | 1.35854 | −3.321982 | −1.386763 |
| 6 | 0.711555 | −2.465495 | −3.25879 |
| 1 | 0.014503 | −3.270405 | −3.474935 |
| 6 | 0.84873 | −1.398182 | −4.151622 |
| 1 | 0.259214 | −1.371692 | −5.063979 |
| 6 | 1.74498 | −0.365945 | −3.865122 |
| 1 | 1.855274 | 0.468226 | −4.553031 |
| 6 | 2.496586 | −0.399918 | −2.688361 |
| 1 | 3.180997 | 0.410595 | −2.456831 |
| 6 | 2.796262 | −1.738588 | 1.988333 |
| 6 | 2.655871 | −3.195179 | 2.436515 |
| 6 | 4.875389 | −3.613047 | 1.567797 |
| 6 | 5.211921 | −2.142403 | 1.823845 |
| 1 | 1.685226 | −3.547113 | 2.08494 |
| 1 | 5.678275 | −1.801042 | 0.891986 |
| 8 | 1.954893 | −0.92501 | 2.648234 |
| 8 | 4.105609 | −1.266372 | 2.097225 |
| 8 | 5.729387 | −4.368635 | 1.160567 |
| 8 | 3.616112 | −4.045461 | 1.748848 |
| 6 | 6.231384 | −2.006811 | 2.955051 |
| 1 | 7.12521 | −2.588595 | 2.715984 |
| 1 | 5.818187 | −2.36147 | 3.903473 |
| 1 | 6.50341 | −0.953873 | 3.067958 |

| | | | |
|---|---|---|---|
| 6 | 2.776729 | −3.365276 | 3.943354 |
| 1 | 3.726488 | −2.967296 | 4.311957 |
| 1 | 2.713859 | −4.4264 | 4.200357 |
| 1 | 1.965472 | −2.822918 | 4.434185 |
| 1 | 1.102196 | 2.04799 | 4.861169 |
| 1 | −1.697044 | −0.636923 | 3.035148 |
| 17 | −1.551779 | −0.677017 | 7.925015 |

**Table S47.** Cartesian coordinate of **L$_{ax}$3b.**

| The number of atom coordinates (angstroms) | X | Y | Z |
|---|---|---|---|
| 13 | 4.977647 | −3.24924 | −3.824582 |
| 8 | 6.752438 | −3.506705 | −3.603375 |
| 8 | 4.274996 | −4.033526 | −5.208855 |
| 7 | 5.186779 | −1.423966 | −4.500764 |
| 6 | 9.101535 | −3.301302 | −3.668121 |
| 1 | 9.190635 | −3.539838 | −2.601576 |
| 1 | 9.91049 | −2.625297 | −3.953633 |
| 1 | 9.207251 | −4.244491 | −4.216498 |
| 6 | 7.740949 | −2.719809 | −3.935577 |
| 6 | 7.588993 | −1.459307 | −4.480637 |
| 1 | 8.487155 | −0.90422 | −4.720454 |
| 6 | 6.341416 | −0.828286 | −4.751893 |
| 6 | 6.370139 | 0.560165 | −5.349125 |
| 1 | 5.683773 | 0.628255 | −6.198925 |
| 1 | 6.04099 | 1.302655 | −4.614162 |
| 1 | 7.376251 | 0.825157 | −5.679232 |
| 6 | 3.948876 | −0.746027 | −4.749633 |
| 6 | 3.486575 | 0.234322 | −3.8652 |
| 6 | 2.254605 | 0.850182 | −4.0765 |
| 1 | 1.891038 | 1.608892 | −3.39209 |
| 6 | 1.485272 | 0.467729 | −5.175208 |
| 6 | 1.922734 | −0.519364 | −6.057123 |
| 1 | 1.305652 | −0.811064 | −6.900021 |
| 6 | 3.157381 | −1.129716 | −5.838129 |
| 6 | 3.065839 | −4.759311 | −5.223572 |
| 1 | 2.322413 | −4.322284 | −4.540893 |
| 1 | 2.640516 | −4.688808 | −6.238047 |
| 6 | 3.236433 | −6.226326 | −4.868668 |
| 6 | 4.401159 | −6.919174 | −5.216729 |
| 1 | 5.193492 | −6.383996 | −5.731713 |
| 6 | 4.55568 | −8.265472 | −4.878736 |

| | | | |
|---|---|---|---|
| 1 | 5.470204 | −8.788568 | −5.147458 |
| 6 | 3.544104 | −8.938984 | −4.187047 |
| 1 | 3.669274 | −9.983335 | −3.913869 |
| 6 | 2.373791 | −8.25573 | −3.844758 |
| 1 | 1.580714 | −8.762148 | −3.300039 |
| 6 | 2.223547 | −6.909671 | −4.186735 |
| 1 | 1.319823 | −6.379845 | −3.899081 |
| 8 | 2.445309 | −4.337041 | −1.828381 |
| 6 | 1.889353 | −5.430567 | −1.09605 |
| 1 | 1.971473 | −5.252079 | −0.015775 |
| 1 | 2.420547 | −6.361835 | −1.322505 |
| 6 | 0.439332 | −5.586252 | −1.493488 |
| 6 | −0.288304 | −4.529867 | −2.0511 |
| 1 | 0.204338 | −3.579387 | −2.225301 |
| 6 | −1.629802 | −4.707185 | −2.398385 |
| 1 | −2.183683 | −3.88152 | −2.837444 |
| 6 | −2.257253 | −5.936364 | −2.187276 |
| 1 | −3.300009 | −6.072491 | −2.461017 |
| 6 | −1.534295 | −6.993621 | −1.628415 |
| 1 | −2.011414 | −7.95659 | −1.466893 |
| 6 | −0.192955 | −6.819064 | −1.287451 |
| 1 | 0.37082 | −7.648327 | −0.864995 |
| 6 | 3.78988 | −3.974629 | −1.707531 |
| 6 | 4.317998 | −3.900876 | −0.262974 |
| 6 | 4.911809 | −6.278774 | −0.396653 |
| 6 | 5.064284 | −6.166675 | −1.912086 |
| 1 | 3.559229 | −3.418252 | 0.357738 |
| 1 | 4.413818 | −6.935655 | −2.342093 |
| 8 | 3.968153 | −2.825478 | −2.39245 |
| 8 | 4.658124 | −4.895536 | −2.441336 |
| 8 | 5.151565 | −7.324857 | 0.162792 |
| 8 | 4.456554 | −5.229829 | 0.318774 |
| 6 | 6.506476 | −6.437821 | −2.330822 |
| 1 | 6.797302 | −7.432285 | −1.984382 |
| 1 | 7.182736 | −5.688665 | −1.911773 |
| 1 | 6.574143 | −6.395883 | −3.419109 |
| 6 | 5.646471 | −3.16626 | −0.15784 |
| 1 | 6.379506 | −3.594396 | −0.848378 |
| 1 | 6.025355 | −3.245541 | 0.864913 |
| 1 | 5.51053 | −2.11342 | −0.413226 |
| 1 | 3.508245 | −1.920671 | −6.490925 |
| 1 | 4.088214 | 0.499924 | −3.001665 |

| | | | |
|---|---|---|---|
| 17 | −0.076296 | 1.239693 | −5.446088 |

**Table S48.** Cartesian coordinate of $L_{ax}$**TS2b.**

| The number of atom coordinates (angstroms) | X | Y | Z |
|---|---|---|---|
| 13 | 4.824145 | −3.427695 | −3.968968 |
| 8 | 6.632935 | −3.838718 | −4.101346 |
| 8 | 4.241988 | −4.110827 | −5.469248 |
| 7 | 5.199486 | −1.514948 | −4.304426 |
| 6 | 8.939645 | −3.886294 | −4.605304 |
| 1 | 9.210366 | −4.29089 | −3.622641 |
| 1 | 9.756796 | −3.259822 | −4.970243 |
| 1 | 8.80819 | −4.74023 | −5.279364 |
| 6 | 7.638877 | −3.136297 | −4.495462 |
| 6 | 7.556104 | −1.784065 | −4.797947 |
| 1 | 8.454114 | −1.282409 | −5.135299 |
| 6 | 6.372056 | −1.019068 | −4.717842 |
| 6 | 6.471713 | 0.429862 | −5.14772 |
| 1 | 6.328105 | 1.104732 | −4.29782 |
| 1 | 7.447737 | 0.636142 | −5.590236 |
| 1 | 5.692467 | 0.670694 | −5.877643 |
| 6 | 4.071631 | −0.623967 | −4.299381 |
| 6 | 3.903821 | 0.313552 | −3.276403 |
| 6 | 2.788035 | 1.149446 | −3.256919 |
| 1 | 2.655411 | 1.878099 | −2.464424 |
| 6 | 1.838286 | 1.032978 | −4.271108 |
| 6 | 1.985175 | 0.098763 | −5.295848 |
| 1 | 1.232179 | 0.017261 | −6.072257 |
| 6 | 3.105348 | −0.730743 | −5.304155 |
| 6 | 3.216608 | −5.050675 | −5.645275 |
| 1 | 2.262766 | −4.687534 | −5.231713 |
| 1 | 3.056021 | −5.187635 | −6.727768 |
| 6 | 3.510507 | −6.413095 | −5.028042 |
| 6 | 4.823449 | −6.832876 | −4.78343 |
| 1 | 5.644514 | −6.16616 | −5.027152 |
| 6 | 5.076284 | −8.078034 | −4.202348 |
| 1 | 6.102112 | −8.385007 | −4.011615 |
| 6 | 4.018714 | −8.926506 | −3.862941 |
| 1 | 4.215324 | −9.891929 | −3.403807 |
| 6 | 2.706206 | −8.519872 | −4.116029 |
| 1 | 1.870884 | −9.161849 | −3.848945 |
| 6 | 2.458067 | −7.273389 | −4.694235 |

| | | | |
|---|---|---|---|
| 1 | 1.43216 | −6.955972 | −4.862411 |
| 8 | 1.948194 | −4.674768 | −2.633967 |
| 6 | 1.465403 | −5.725188 | −1.762046 |
| 1 | 1.255364 | −5.32237 | −0.767375 |
| 1 | 2.251373 | −6.479695 | −1.67293 |
| 6 | 0.221783 | −6.31478 | −2.378054 |
| 6 | −0.638763 | −5.547067 | −3.169443 |
| 1 | −0.391283 | −4.511853 | −3.381461 |
| 6 | −1.794238 | −6.117686 | −3.706948 |
| 1 | −2.450883 | −5.515929 | −4.329418 |
| 6 | −2.105184 | −7.454432 | −3.44888 |
| 1 | −3.003609 | −7.897256 | −3.869828 |
| 6 | −1.252047 | −8.221367 | −2.651637 |
| 1 | −1.482445 | −9.264045 | −2.450783 |
| 6 | −0.09182 | −7.654553 | −2.123285 |
| 1 | 0.581147 | −8.259756 | −1.520709 |
| 6 | 2.800748 | −3.721854 | −2.273847 |
| 6 | 3.019358 | −3.417038 | −0.788679 |
| 6 | 4.47305 | −5.404044 | −0.25464 |
| 6 | 5.253023 | −5.352002 | −1.574785 |
| 1 | 2.031688 | −3.176365 | −0.373433 |
| 1 | 5.18985 | −6.375694 | −1.973415 |
| 8 | 3.067899 | −2.869085 | −3.163705 |
| 8 | 4.651219 | −4.461022 | −2.460268 |
| 8 | 4.742653 | −6.216855 | 0.599734 |
| 8 | 3.411952 | −4.576102 | −0.017747 |
| 6 | 6.725166 | −5.037195 | −1.276546 |
| 1 | 7.102302 | −5.723725 | −0.514059 |
| 1 | 6.83225 | −4.00786 | −0.916509 |
| 1 | 7.305766 | −5.144187 | −2.192901 |
| 6 | 3.951122 | −2.240724 | −0.548888 |
| 1 | 4.943566 | −2.446005 | −0.954443 |
| 1 | 4.030514 | −2.061898 | 0.526766 |
| 1 | 3.552933 | −1.347806 | −1.035881 |
| 1 | 4.648855 | 0.383664 | −2.489478 |
| 1 | 3.228217 | −1.486651 | −6.073012 |
| 17 | 0.421394 | 2.083297 | −4.252333 |

**Table S49.** Cartesian coordinate of **$L_{ax}$4b.**

| The number of atom coordinates (angstroms) | X | Y | Z |
|---|---|---|---|
| 13 | 5.095466 | −3.499157 | −4.17376 |

| | | | |
|---|---|---|---|
| 8 | 6.763416 | −4.171915 | −4.402403 |
| 8 | 4.089173 | −3.874563 | −5.524576 |
| 7 | 5.585794 | −1.645864 | −4.339039 |
| 6 | 9.065141 | −4.370245 | −4.890241 |
| 1 | 9.250383 | −4.943306 | −3.974145 |
| 1 | 9.954523 | −3.786597 | −5.136778 |
| 1 | 8.875035 | −5.096611 | −5.688793 |
| 6 | 7.846213 | −3.512618 | −4.700764 |
| 6 | 7.90094 | −2.134899 | −4.837897 |
| 1 | 8.855456 | −1.699359 | −5.104855 |
| 6 | 6.810827 | −1.24012 | −4.672314 |
| 6 | 7.085114 | 0.228198 | −4.905195 |
| 1 | 7.1364 | 0.769741 | −3.954638 |
| 1 | 8.033773 | 0.366233 | −5.426892 |
| 1 | 6.282186 | 0.685378 | −5.490704 |
| 6 | 4.532376 | −0.684812 | −4.15985 |
| 6 | 4.52127 | 0.159647 | −3.045974 |
| 6 | 3.469894 | 1.051729 | −2.849584 |
| 1 | 3.454231 | 1.70728 | −1.985671 |
| 6 | 2.427662 | 1.08223 | −3.775713 |
| 6 | 2.42002 | 0.241149 | −4.886494 |
| 1 | 1.594118 | 0.27107 | −5.588507 |
| 6 | 3.476248 | −0.647381 | −5.074759 |
| 6 | 2.995828 | −4.763653 | −5.594497 |
| 1 | 2.127489 | −4.342219 | −5.070411 |
| 1 | 2.714835 | −4.866215 | −6.654629 |
| 6 | 3.278665 | −6.146276 | −5.025188 |
| 6 | 4.576041 | −6.671148 | −4.980693 |
| 1 | 5.403298 | −6.092234 | −5.379281 |
| 6 | 4.818244 | −7.928274 | −4.419331 |
| 1 | 5.834401 | −8.313255 | −4.381347 |
| 6 | 3.762364 | −8.687975 | −3.911886 |
| 1 | 3.950975 | −9.661818 | −3.467874 |
| 6 | 2.460752 | −8.183441 | −3.979111 |
| 1 | 1.626634 | −8.758271 | −3.58544 |
| 6 | 2.224944 | −6.923632 | −4.530113 |
| 1 | 1.213906 | −6.527949 | −4.549706 |
| 8 | 1.544694 | −4.554022 | −2.543519 |
| 6 | 1.123445 | −5.604602 | −1.64752 |
| 1 | 0.937641 | −5.203908 | −0.64716 |
| 1 | 1.923893 | −6.344952 | −1.570667 |
| 6 | −0.134723 | −6.234968 | −2.196137 |

| | | | |
|---|---|---|---|
| 6 | −1.037775 | −5.510153 | −2.981147 |
| 1 | −0.808783 | −4.482368 | −3.244129 |
| 6 | −2.208773 | −6.113714 | −3.444275 |
| 1 | −2.899305 | −5.544025 | −4.060492 |
| 6 | −2.492452 | −7.442412 | −3.121095 |
| 1 | −3.403116 | −7.910885 | −3.484266 |
| 6 | −1.595392 | −8.168437 | −2.333856 |
| 1 | −1.804013 | −9.204899 | −2.082582 |
| 6 | −0.420895 | −7.568124 | −1.878157 |
| 1 | 0.282053 | −8.139837 | −1.276401 |
| 6 | 2.141126 | −3.402257 | −2.165801 |
| 6 | 2.6196 | −3.24488 | −0.716999 |
| 6 | 4.201272 | −5.224144 | −0.52237 |
| 6 | 5.126445 | −4.860134 | −1.6886 |
| 1 | 1.704523 | −3.103045 | −0.125517 |
| 1 | 5.314177 | −5.8248 | −2.180904 |
| 8 | 2.160019 | −2.477994 | −2.948121 |
| 8 | 4.545353 | −3.949914 | −2.577823 |
| 8 | 4.411848 | −6.227311 | 0.124627 |
| 8 | 3.15883 | −4.446813 | −0.122444 |
| 6 | 6.450322 | −4.349143 | −1.089014 |
| 1 | 6.856547 | −5.07765 | −0.38141 |
| 1 | 6.293201 | −3.397965 | −0.56967 |
| 1 | 7.173593 | −4.193333 | −1.893249 |
| 6 | 3.504891 | −2.02583 | −0.512247 |
| 1 | 4.394019 | −2.092102 | −1.136811 |
| 1 | 3.786554 | −1.959764 | 0.542893 |
| 1 | 2.955374 | −1.125983 | −0.797052 |
| 1 | 3.47552 | −1.337396 | −5.911351 |
| 1 | 5.328372 | 0.104565 | −2.322163 |
| 17 | 1.089919 | 2.202773 | −3.528906 |

**Table S50.** Cartesian coordinate of **L$_{ax}$5a.**

| The number of atom coordinates (angstroms) | X | Y | Z |
|---|---|---|---|
| 13 | 0.849442 | 0.849442 | 1.376941 |
| 8 | 1.060621 | 1.060621 | −0.19927 |
| 8 | −0.713645 | −0.713645 | 1.650009 |
| 7 | 0.381579 | 0.381579 | 2.358735 |
| 6 | 0.651888 | 0.651888 | −2.00654 |
| 1 | 1.684358 | 1.684358 | −2.348036 |
| 1 | 0.308216 | 0.308216 | −2.24853 |

| | | | |
|---|---|---|---|
| 1 | 0.040833 | 0.040833 | −2.548052 |
| 6 | 0.574908 | 0.574908 | −0.534875 |
| 6 | 0.024326 | 0.024326 | 0.354743 |
| 1 | −0.348958 | −0.348958 | −0.04581 |
| 6 | −0.017437 | −0.017437 | 1.769118 |
| 6 | −0.536669 | −0.536669 | 2.585346 |
| 1 | −1.182074 | −1.182074 | 3.397404 |
| 1 | 0.291879 | 0.291879 | 3.044897 |
| 1 | −1.095089 | −1.095089 | 1.955154 |
| 6 | 0.404849 | 0.404849 | 3.785256 |
| 6 | 1.371458 | 1.371458 | 4.528516 |
| 6 | 1.423267 | 1.423267 | 5.915047 |
| 1 | 2.173572 | 2.173572 | 6.492769 |
| 6 | 0.502635 | 0.502635 | 6.552239 |
| 6 | −0.458229 | −0.458229 | 5.827916 |
| 1 | −1.160989 | −1.160989 | 6.340362 |
| 6 | −0.502437 | −0.502437 | 4.440822 |
| 6 | −1.272365 | −1.272365 | 0.98732 |
| 1 | −0.634137 | −0.634137 | 1.064039 |
| 1 | −2.221099 | −2.221099 | 1.488181 |
| 6 | −1.565375 | −1.565375 | −0.485335 |
| 6 | −1.955783 | −1.955783 | −0.922948 |
| 1 | −2.010261 | −2.010261 | −0.20263 |
| 6 | −2.25335 | −2.25335 | −2.267295 |
| 1 | −2.55582 | −2.55582 | −2.589744 |
| 6 | −2.166932 | −2.166932 | −3.196955 |
| 1 | −2.394291 | −2.394291 | −4.244395 |
| 6 | −1.776124 | −1.776124 | −2.769879 |
| 1 | −1.690982 | −1.690982 | −3.486256 |
| 6 | −1.475144 | −1.475144 | −1.424207 |
| 1 | −1.158211 | −1.158211 | −1.099749 |
| 8 | 5.304909 | 5.304909 | −1.53876 |
| 6 | 4.01909 | 4.01909 | −2.200768 |
| 1 | 3.298377 | 3.298377 | −1.543986 |
| 1 | 4.268266 | 4.268266 | −3.010419 |
| 6 | 3.486292 | 3.486292 | −2.74093 |
| 6 | 2.148389 | 2.148389 | −2.552614 |
| 1 | 1.490995 | 1.490995 | −1.966224 |
| 6 | 1.652695 | 1.652695 | −3.083052 |
| 1 | 0.617183 | 0.617183 | −2.905084 |
| 6 | 2.495571 | 2.495571 | −3.809733 |
| 1 | 2.112266 | 2.112266 | −4.22338 |

| | | | |
|---|---|---|---|
| 6 | 3.837213 | 3.837213 | −3.99827 |
| 1 | 4.497925 | 4.497925 | −4.558664 |
| 6 | 4.330435 | 4.330435 | −3.467595 |
| 1 | 5.374179 | 5.374179 | −3.603497 |
| 6 | 5.502162 | 5.502162 | −0.239569 |
| 6 | 4.395299 | 4.395299 | 0.815696 |
| 6 | 2.563208 | 2.563208 | 0.928344 |
| 6 | 3.021105 | 3.021105 | 2.232033 |
| 1 | 4.758451 | 4.758451 | 1.689921 |
| 1 | 4.101439 | 4.101439 | 2.167612 |
| 8 | 6.615464 | 6.615464 | 0.148082 |
| 8 | 2.307729 | 2.307729 | 2.364168 |
| 8 | 1.557176 | 1.557176 | 0.3796 |
| 8 | 3.128299 | 3.128299 | 0.375506 |
| 6 | 2.743617 | 2.743617 | 3.42277 |
| 1 | 3.333387 | 3.333387 | 3.400207 |
| 1 | 1.679472 | 1.679472 | 3.445541 |
| 1 | 2.981351 | 2.981351 | 4.338792 |
| 6 | 4.186922 | 4.186922 | 1.145758 |
| 1 | 3.828405 | 3.828405 | 0.275155 |
| 1 | 3.45278 | 3.45278 | 1.950622 |
| 1 | 5.134217 | 5.134217 | 1.475074 |
| 1 | 2.09574 | 2.09574 | 4.013144 |
| 1 | −1.219045 | −1.219045 | 3.847528 |
| 17 | 0.563399 | 0.563399 | 8.307874 |

**Table S51.** Cartesian coordinate of **L$_{ax}$1c.**

| The number of atom coordinates (angstroms) | X | Y | Z |
|---|---|---|---|
| 13 | 0.837864 | 0.301025 | 1.087225 |
| 8 | 0.420948 | 1.524609 | −0.251647 |
| 8 | −0.433232 | −0.890408 | 0.783961 |
| 7 | 0.474737 | 1.616683 | 2.515383 |
| 6 | −0.520109 | 3.294296 | −1.500345 |
| 1 | 0.347174 | 3.319051 | −2.170287 |
| 1 | −0.922744 | 4.304331 | −1.393378 |
| 1 | −1.276252 | 2.650783 | −1.96331 |
| 6 | −0.12096 | 2.688911 | −0.18307 |
| 6 | −0.330978 | 3.357923 | 1.015866 |
| 1 | −0.764728 | 4.349042 | 0.972593 |
| 6 | −0.023682 | 2.840925 | 2.293582 |
| 6 | −0.33677 | 3.75284 | 3.463452 |

| | | | |
|---|---|---|---|
| 1 | 0.449095 | 3.729188 | 4.220831 |
| 1 | −0.477122 | 4.780799 | 3.123293 |
| 1 | −1.260173 | 3.427714 | 3.954875 |
| 6 | 0.682519 | 1.248406 | 3.901941 |
| 6 | 1.988817 | 1.312888 | 4.43262 |
| 6 | 2.172557 | 0.965503 | 5.774446 |
| 1 | 3.165523 | 1.017692 | 6.209896 |
| 6 | 1.106889 | 0.529291 | 6.559604 |
| 6 | −0.164324 | 0.428011 | 6.004222 |
| 1 | −0.986031 | 0.062361 | 6.613293 |
| 6 | −0.403757 | 0.785871 | 4.670823 |
| 6 | −1.080224 | −1.146184 | −0.435559 |
| 1 | −0.362555 | −1.250097 | −1.262271 |
| 1 | −1.588141 | −2.1229 | −0.3487 |
| 6 | −2.123677 | −0.113517 | −0.828579 |
| 6 | −2.774653 | 0.656515 | 0.140117 |
| 1 | −2.498122 | 0.524528 | 1.180501 |
| 6 | −3.743399 | 1.592401 | −0.225423 |
| 1 | −4.229465 | 2.192407 | 0.540264 |
| 6 | −4.086691 | 1.761333 | −1.569331 |
| 1 | −4.840293 | 2.490438 | −1.855572 |
| 6 | −3.447609 | 0.989405 | −2.543995 |
| 1 | −3.701419 | 1.118371 | −3.593491 |
| 6 | −2.468897 | 0.063862 | −2.173953 |
| 1 | −1.954118 | −0.517468 | −2.936196 |
| 8 | 2.491061 | −0.031922 | 0.526411 |
| 6 | 3.005398 | 0.30683 | −0.744529 |
| 1 | 4.095692 | 0.156035 | −0.718091 |
| 1 | 2.830129 | 1.364672 | −0.987971 |
| 6 | 2.408826 | −0.561184 | −1.839348 |
| 6 | 2.662142 | −1.940987 | −1.850124 |
| 1 | 3.332375 | −2.366431 | −1.106859 |
| 6 | 2.057237 | −2.772815 | −2.792691 |
| 1 | 2.260336 | −3.840708 | −2.780727 |
| 6 | 1.198822 | −2.231384 | −3.755294 |
| 1 | 0.728194 | −2.876555 | −4.49264 |
| 6 | 0.952757 | −0.856985 | −3.764437 |
| 1 | 0.286258 | −0.427253 | −4.508291 |
| 6 | 1.549091 | −0.031114 | −2.807196 |
| 1 | 1.322917 | 1.030511 | −2.789709 |
| 6 | 2.023813 | −2.054118 | 2.483989 |
| 6 | 1.705389 | −3.003239 | 1.348542 |

| | | | |
|---|---|---|---|
| 6 | 3.633737 | −4.341231 | 1.972408 |
| 6 | 3.579087 | −3.69723 | 3.353129 |
| 1 | 1.559944 | −2.387956 | 0.464329 |
| 1 | 4.614189 | −3.482561 | 3.625964 |
| 8 | 1.449477 | −0.983979 | 2.629914 |
| 8 | 2.932504 | −2.396007 | 3.384821 |
| 1 | 1.271667 | 0.256953 | 7.599029 |
| 6 | −1.801334 | 0.614495 | 4.083395 |
| 1 | −1.843509 | 1.16398 | 3.139091 |
| 6 | −2.067505 | −0.86803 | 3.756363 |
| 1 | −3.075627 | −0.993361 | 3.341759 |
| 1 | −1.351642 | −1.232332 | 3.015826 |
| 1 | −1.992547 | −1.481486 | 4.662736 |
| 6 | −2.908857 | 1.180441 | 4.989969 |
| 1 | −3.017946 | 0.59699 | 5.911283 |
| 1 | −2.709558 | 2.219247 | 5.277255 |
| 1 | −3.872329 | 1.148966 | 4.468244 |
| 6 | 3.173236 | 1.726182 | 3.563847 |
| 1 | 2.932322 | 1.471179 | 2.528129 |
| 6 | 3.415514 | 3.246184 | 3.622716 |
| 1 | 3.611463 | 3.570483 | 4.652369 |
| 1 | 4.281853 | 3.521041 | 3.008972 |
| 1 | 2.552363 | 3.806084 | 3.249132 |
| 6 | 4.459782 | 0.956048 | 3.904052 |
| 1 | 4.876864 | 1.251455 | 4.874396 |
| 1 | 4.274144 | −0.122823 | 3.922404 |
| 1 | 5.22372 | 1.16151 | 3.145601 |
| 8 | 4.411881 | −5.236678 | 1.747169 |
| 8 | 2.816315 | −3.87248 | 1.015827 |
| 6 | 2.950033 | −4.61509 | 4.398003 |
| 1 | 3.512432 | −5.551653 | 4.435753 |
| 1 | 1.907102 | −4.839019 | 4.157817 |
| 1 | 2.988223 | −4.131011 | 5.377156 |
| 6 | 0.440904 | −3.815586 | 1.621725 |
| 1 | 0.527609 | −4.424382 | 2.526355 |
| 1 | 0.264969 | −4.476544 | 0.768767 |
| 1 | −0.39593 | −3.121346 | 1.718241 |

**Table S52.** Cartesian coordinate of **$L_{ax}$TS1c.**

| The number of atom coordinates (angstroms) | X | Y | Z |
|---|---|---|---|
| 13 | 0.910619 | 0.104662 | 1.381513 |

| | | | |
|---|---|---|---|
| 8 | 1.262082 | 1.454627 | 0.162339 |
| 8 | −0.436746 | −0.783132 | 0.692767 |
| 7 | 0.254637 | 1.418518 | 2.705666 |
| 6 | 0.778918 | 3.256024 | −1.294543 |
| 1 | 1.836066 | 3.339292 | −1.570905 |
| 1 | 0.319201 | 4.246557 | −1.315557 |
| 1 | 0.287575 | 2.61886 | −2.039079 |
| 6 | 0.653072 | 2.591849 | 0.047938 |
| 6 | −0.072001 | 3.174649 | 1.074774 |
| 1 | −0.537743 | 4.133166 | 0.88501 |
| 6 | −0.207743 | 2.624551 | 2.369887 |
| 6 | −0.918319 | 3.472386 | 3.40169 |
| 1 | −0.279809 | 3.653199 | 4.270706 |
| 1 | −1.221769 | 4.432304 | 2.979977 |
| 1 | −1.807551 | 2.952072 | 3.771829 |
| 6 | 0.178354 | 1.042559 | 4.101316 |
| 6 | 1.261828 | 1.361817 | 4.946189 |
| 6 | 1.198766 | 0.958912 | 6.284857 |
| 1 | 2.02379 | 1.196381 | 6.951359 |
| 6 | 0.102718 | 0.252985 | 6.774238 |
| 6 | −0.953897 | −0.056811 | 5.922685 |
| 1 | −1.804793 | −0.613539 | 6.305979 |
| 6 | −0.940261 | 0.329726 | 4.577001 |
| 6 | −0.817436 | −0.931796 | −0.649149 |
| 1 | 0.042427 | −1.149643 | −1.29796 |
| 1 | −1.481069 | −1.811233 | −0.718633 |
| 6 | −1.564707 | 0.259098 | −1.230452 |
| 6 | −2.304112 | 1.117599 | −0.412054 |
| 1 | −2.309928 | 0.941368 | 0.657995 |
| 6 | −3.001125 | 2.19876 | −0.95503 |
| 1 | −3.560534 | 2.862623 | −0.300104 |
| 6 | −2.975276 | 2.433157 | −2.331272 |
| 1 | −3.514193 | 3.276964 | −2.754583 |
| 6 | −2.247836 | 1.574026 | −3.160368 |
| 1 | −2.217289 | 1.748668 | −4.233325 |
| 6 | −1.54723 | 0.498161 | −2.611522 |
| 1 | −0.96771 | −0.15769 | −3.256162 |
| 8 | 2.538947 | −0.718049 | 0.950904 |
| 6 | 3.429101 | −0.642121 | −0.165753 |
| 1 | 4.273459 | −1.312641 | 0.032979 |
| 1 | 3.822501 | 0.379589 | −0.212084 |
| 6 | 2.78231 | −1.005335 | −1.485549 |

| | | | |
|---|---|---|---|
| 6 | 2.534644 | −2.343236 | −1.823652 |
| 1 | 2.831334 | −3.133905 | −1.141824 |
| 6 | 1.913795 | −2.666224 | −3.031002 |
| 1 | 1.726059 | −3.707548 | −3.279261 |
| 6 | 1.544675 | −1.656109 | −3.923692 |
| 1 | 1.065594 | −1.908184 | −4.86619 |
| 6 | 1.800001 | −0.321708 | −3.600416 |
| 1 | 1.515739 | 0.470689 | −4.287763 |
| 6 | 2.410375 | −0.001341 | −2.386805 |
| 1 | 2.58725 | 1.036001 | −2.124669 |
| 6 | 2.225633 | −1.858338 | 2.398531 |
| 6 | 1.841476 | −3.161035 | 1.720384 |
| 6 | 4.224339 | −3.59776 | 1.494112 |
| 6 | 4.48936 | −2.643394 | 2.656266 |
| 1 | 1.134485 | −2.92735 | 0.92573 |
| 1 | 5.216718 | −1.916161 | 2.281368 |
| 8 | 1.297679 | −1.11365 | 2.843797 |
| 8 | 3.356791 | −1.880492 | 3.121485 |
| 1 | 0.072992 | −0.055681 | 7.816057 |
| 6 | −2.108538 | −0.045454 | 3.67245 |
| 1 | −1.958541 | 0.443435 | 2.707202 |
| 6 | −2.132403 | −1.561184 | 3.401662 |
| 1 | −2.973906 | −1.816781 | 2.746497 |
| 1 | −1.21113 | −1.868338 | 2.903685 |
| 1 | −2.243391 | −2.124418 | 4.336677 |
| 6 | −3.459658 | 0.433563 | 4.234277 |
| 1 | −3.715956 | −0.082913 | 5.166664 |
| 1 | −3.455501 | 1.509714 | 4.443099 |
| 1 | −4.260703 | 0.230238 | 3.513892 |
| 6 | 2.489716 | 2.116679 | 4.444607 |
| 1 | 2.341693 | 2.341868 | 3.384396 |
| 6 | 2.676708 | 3.45812 | 5.178383 |
| 1 | 2.859082 | 3.303946 | 6.248375 |
| 1 | 3.536853 | 4.000657 | 4.768328 |
| 1 | 1.794711 | 4.100731 | 5.080486 |
| 6 | 3.761227 | 1.254915 | 4.555164 |
| 1 | 3.991456 | 1.022522 | 5.602136 |
| 1 | 3.641216 | 0.310467 | 4.019183 |
| 1 | 4.621532 | 1.791453 | 4.136049 |
| 8 | 5.149223 | −4.155959 | 0.950535 |
| 8 | 2.967071 | −3.797838 | 1.066139 |
| 6 | 5.073355 | −3.391164 | 3.851794 |

| | | | |
|---|---|---|---|
| 1 | 5.987789 | −3.906754 | 3.549679 |
| 1 | 4.35834 | −4.124919 | 4.236619 |
| 1 | 5.303727 | −2.677262 | 4.646959 |
| 6 | 1.229114 | −4.122261 | 2.735642 |
| 1 | 1.931874 | −4.333072 | 3.548083 |
| 1 | 0.972842 | −5.060599 | 2.236493 |
| 1 | 0.324311 | −3.680914 | 3.160569 |

**Table S53.** Cartesian coordinate of **L$_{ax}$2c.**

| The number of atom coordinates (angstroms) | X | Y | Z |
|---|---|---|---|
| 13 | 0.771796 | −0.211804 | 1.432724 |
| 8 | 1.511908 | 0.971574 | 0.279769 |
| 8 | −0.650828 | −1.079548 | 0.97391 |
| 7 | 0.107386 | 1.194417 | 2.644165 |
| 6 | 1.74193 | 2.867359 | −1.11162 |
| 1 | 2.83555 | 2.877202 | −1.024894 |
| 1 | 1.385538 | 3.890155 | −1.25306 |
| 1 | 1.491676 | 2.268154 | −1.991816 |
| 6 | 1.156112 | 2.220363 | 0.112026 |
| 6 | 0.352245 | 2.920524 | 0.984598 |
| 1 | 0.109746 | 3.946589 | 0.735957 |
| 6 | −0.094402 | 2.439298 | 2.251116 |
| 6 | −0.81183 | 3.443817 | 3.123245 |
| 1 | −0.928008 | 3.100769 | 4.150901 |
| 1 | −0.28354 | 4.401864 | 3.11852 |
| 1 | −1.809332 | 3.618979 | 2.701271 |
| 6 | −0.184519 | 0.791756 | 4.000461 |
| 6 | 0.856408 | 0.858842 | 4.953113 |
| 6 | 0.583256 | 0.430473 | 6.256482 |
| 1 | 1.364314 | 0.486404 | 7.009219 |
| 6 | −0.670851 | −0.065644 | 6.60691 |
| 6 | −1.678098 | −0.134139 | 5.649886 |
| 1 | −2.655962 | −0.519608 | 5.925779 |
| 6 | −1.458623 | 0.290348 | 4.332656 |
| 6 | −1.489347 | −1.187729 | −0.148475 |
| 1 | −1.00659 | −1.796196 | −0.927408 |
| 1 | −2.403153 | −1.716472 | 0.160238 |
| 6 | −1.870553 | 0.156505 | −0.74173 |
| 6 | −2.961079 | 0.880976 | −0.242676 |
| 1 | −3.576774 | 0.444665 | 0.539652 |
| 6 | −3.270509 | 2.146444 | −0.744311 |

| | | | |
|---|---|---|---|
| 1 | −4.119788 | 2.695068 | −0.344489 |
| 6 | −2.497262 | 2.699549 | −1.768493 |
| 1 | −2.73975 | 3.681491 | −2.166745 |
| 6 | −1.418402 | 1.978315 | −2.284622 |
| 1 | −0.819434 | 2.395925 | −3.090067 |
| 6 | −1.105446 | 0.718306 | −1.770755 |
| 1 | −0.258494 | 0.166293 | −2.165948 |
| 8 | 2.201238 | −1.640666 | 0.575747 |
| 6 | 3.129283 | −1.452352 | −0.508538 |
| 1 | 3.802029 | −2.3168 | −0.543059 |
| 1 | 3.716599 | −0.546309 | −0.338682 |
| 6 | 2.354765 | −1.364478 | −1.797684 |
| 6 | 1.424397 | −2.357359 | −2.128291 |
| 1 | 1.238979 | −3.165233 | −1.426344 |
| 6 | 0.732157 | −2.301559 | −3.338327 |
| 1 | 0.007109 | −3.073335 | −3.581451 |
| 6 | 0.969354 | −1.255583 | −4.235011 |
| 1 | 0.428985 | −1.211493 | −5.176484 |
| 6 | 1.897275 | −0.263334 | −3.911326 |
| 1 | 2.082623 | 0.556623 | −4.600068 |
| 6 | 2.581881 | −0.316848 | −2.694724 |
| 1 | 3.291435 | 0.464085 | −2.435921 |
| 6 | 2.679421 | −1.781137 | 1.972783 |
| 6 | 2.626769 | −3.259981 | 2.363253 |
| 6 | 4.897443 | −3.50504 | 1.56576 |
| 6 | 5.127282 | −2.024037 | 1.874178 |
| 1 | 1.69125 | −3.662601 | 1.973172 |
| 1 | 5.59868 | −1.62688 | 0.966879 |
| 8 | 1.785908 | −1.051427 | 2.659301 |
| 8 | 3.958552 | −1.23226 | 2.135605 |
| 1 | −0.861268 | −0.396247 | 7.624711 |
| 6 | −2.591746 | 0.221996 | 3.318134 |
| 1 | −2.203657 | 0.573717 | 2.361629 |
| 6 | −3.068521 | −1.224405 | 3.101419 |
| 1 | −3.870348 | −1.251964 | 2.352748 |
| 1 | −2.242396 | −1.841568 | 2.739442 |
| 1 | −3.464663 | −1.661917 | 4.025565 |
| 6 | −3.763344 | 1.137408 | 3.719227 |
| 1 | −4.234247 | 0.800238 | 4.650341 |
| 1 | −3.432896 | 2.171087 | 3.868545 |
| 1 | −4.533234 | 1.135814 | 2.937656 |
| 6 | 2.229306 | 1.425637 | 4.607533 |

| | | | |
|---|---|---|---|
| 1 | 2.310203 | 1.470892 | 3.519324 |
| 6 | 2.370752 | 2.861323 | 5.149915 |
| 1 | 2.293574 | 2.874247 | 6.24407 |
| 1 | 3.344204 | 3.284496 | 4.873861 |
| 1 | 1.589257 | 3.519119 | 4.75347 |
| 6 | 3.379869 | 0.533067 | 5.099956 |
| 1 | 3.426601 | 0.488655 | 6.194578 |
| 1 | 3.267325 | −0.478819 | 4.705893 |
| 1 | 4.338027 | 0.931885 | 4.746006 |
| 8 | 5.81419 | −4.194258 | 1.177845 |
| 8 | 3.659721 | −4.018384 | 1.673273 |
| 6 | 6.099893 | −1.851939 | 3.041263 |
| 1 | 7.039662 | −2.362654 | 2.816721 |
| 1 | 5.683173 | −2.26094 | 3.965913 |
| 1 | 6.291929 | −0.786133 | 3.191893 |
| 6 | 2.722029 | −3.479813 | 3.86572 |
| 1 | 3.641191 | −3.04679 | 4.270936 |
| 1 | 2.711802 | −4.551655 | 4.082755 |
| 1 | 1.873253 | −2.999206 | 4.357092 |

**Table S54.** Cartesian coordinate of **L$_{ax}$3c.**

| The number of atom coordinates (angstroms) | X | Y | Z |
|---|---|---|---|
| 13 | 4.869034 | −3.196208 | −3.85189 |
| 8 | 6.61806 | −3.615902 | −3.965925 |
| 8 | 3.822919 | −3.894538 | −5.038539 |
| 7 | 5.075502 | −1.358307 | −4.461085 |
| 6 | 8.962242 | −3.38756 | −3.795888 |
| 1 | 8.995262 | −3.88688 | −2.820017 |
| 1 | 9.774664 | −2.659774 | −3.852842 |
| 1 | 9.115236 | −4.162111 | −4.556057 |
| 6 | 7.609639 | −2.760192 | −3.99013 |
| 6 | 7.465633 | −1.402257 | −4.187643 |
| 1 | 8.364323 | −0.796688 | −4.197955 |
| 6 | 6.239083 | −0.735531 | −4.491795 |
| 6 | 6.351122 | 0.722681 | −4.868881 |
| 1 | 5.392009 | 1.156798 | −5.148996 |
| 1 | 6.75822 | 1.283074 | −4.019129 |
| 1 | 7.054602 | 0.840186 | −5.699928 |
| 6 | 3.857118 | −0.708261 | −4.890573 |
| 6 | 3.048686 | −0.044575 | −3.944614 |
| 6 | 1.84019 | 0.508801 | −4.388827 |

| | | | |
|---|---|---|---|
| 1 | 1.205159 | 1.03111 | −3.678814 |
| 6 | 1.444082 | 0.408383 | −5.718623 |
| 6 | 2.260977 | −0.248172 | −6.635832 |
| 1 | 1.952062 | −0.315644 | −7.675111 |
| 6 | 3.47558 | −0.822347 | −6.245291 |
| 6 | 2.62452 | −4.599607 | −4.815903 |
| 1 | 2.104356 | −4.24172 | −3.916078 |
| 1 | 1.951364 | −4.401907 | −5.665963 |
| 6 | 2.823495 | −6.100328 | −4.688589 |
| 6 | 3.923283 | −6.733882 | −5.275257 |
| 1 | 4.644229 | −6.129836 | −5.817482 |
| 6 | 4.108009 | −8.111628 | −5.136404 |
| 1 | 4.973635 | −8.588623 | −5.58976 |
| 6 | 3.192026 | −8.875793 | −4.40815 |
| 1 | 3.342048 | −9.945576 | −4.289571 |
| 6 | 2.083071 | −8.25092 | −3.830009 |
| 1 | 1.364842 | −8.831399 | −3.256144 |
| 6 | 1.90052 | −6.873745 | −3.97403 |
| 1 | 1.044286 | −6.39253 | −3.508185 |
| 8 | 2.721404 | −4.165442 | −1.358579 |
| 6 | 2.148646 | −5.349075 | −0.813176 |
| 1 | 2.369544 | −5.428313 | 0.260038 |
| 1 | 2.560783 | −6.236696 | −1.307125 |
| 6 | 0.653933 | −5.330621 | −1.045126 |
| 6 | 0.000087 | −4.237373 | −1.620447 |
| 1 | 0.572329 | −3.3601 | −1.899865 |
| 6 | 1.379846 | −4.280556 | −1.842024 |
| 1 | 1.876376 | −3.425564 | −2.29352 |
| 6 | 2.116844 | −5.411892 | −1.491091 |
| 1 | 3.188596 | −5.444181 | −1.667524 |
| 6 | 1.466492 | −6.506632 | −0.913271 |
| 1 | 2.030394 | −7.394282 | −0.638953 |
| 6 | 0.090615 | −6.46469 | −0.693144 |
| 1 | 0.413949 | −7.32222 | −0.252065 |
| 6 | 4.113259 | −4.054436 | −1.530756 |
| 6 | 4.900595 | −4.136602 | −0.210959 |
| 6 | 5.131951 | −6.547553 | −0.520791 |
| 6 | 5.072117 | −6.34933 | −2.032457 |
| 1 | 4.343609 | −3.579777 | 0.545479 |
| 1 | 4.350198 | −7.085063 | −2.402883 |
| 8 | 4.330007 | −2.887942 | −2.176734 |
| 8 | 4.612591 | −5.053506 | −2.448248 |

| | | | |
|---|---|---|---|
| 1 | 0.502818 | 0.845099 | −6.041899 |
| 6 | 4.359635 | −1.498944 | −7.287526 |
| 1 | 5.189074 | −1.986143 | −6.768615 |
| 6 | 3.614474 | −2.597726 | −8.063305 |
| 1 | 4.294891 | −3.080671 | −8.775099 |
| 1 | 3.24381 | −3.356903 | −7.371288 |
| 1 | 2.771491 | −2.193595 | −8.635823 |
| 6 | 4.959427 | −0.451558 | −8.246633 |
| 1 | 4.171251 | 0.056931 | −8.814813 |
| 1 | 5.523688 | 0.314663 | −7.703174 |
| 1 | 5.636446 | −0.930182 | −8.964231 |
| 6 | 3.462299 | 0.13117 | −2.487273 |
| 1 | 4.384156 | −0.429876 | −2.322173 |
| 6 | 3.749375 | 1.613921 | −2.178819 |
| 1 | 2.847491 | 2.226223 | −2.297551 |
| 1 | 4.098518 | 1.728931 | −1.14565 |
| 1 | 4.517667 | 2.021185 | −2.845794 |
| 6 | 2.414437 | −0.442582 | −1.517071 |
| 1 | 1.451854 | 0.074779 | −1.607662 |
| 1 | 2.266005 | −1.509581 | −1.698862 |
| 1 | 2.758031 | −0.322713 | −0.48226 |
| 8 | 5.328228 | −7.64728 | −0.055223 |
| 8 | 4.923148 | −5.4993 | 0.301954 |
| 6 | 6.430479 | −6.635055 | −2.671647 |
| 1 | 6.725569 | −7.661997 | −2.441449 |
| 1 | 7.196581 | −5.946696 | −2.306708 |
| 1 | 6.343546 | −6.513692 | −3.752504 |
| 6 | 6.322029 | −3.605516 | −0.330018 |
| 1 | 6.846162 | −4.08074 | −1.163723 |
| 1 | 6.865519 | −3.804135 | 0.597984 |
| 1 | 6.30028 | −2.529638 | −0.514979 |

**Table S55.** Cartesian coordinate of **L$_{ax}$TS2c.**

| The number of atom coordinates (angstroms) | X | Y | Z |
|---|---|---|---|
| 13 | 4.791073 | −3.383855 | −3.8334 |
| 8 | 6.620973 | −3.742369 | −3.888776 |
| 8 | 4.30176 | −4.21982 | −5.28654 |
| 7 | 5.116663 | −1.526278 | −4.405489 |
| 6 | 8.67206 | −4.098108 | −5.012849 |
| 1 | 9.210011 | −4.175201 | −4.060669 |
| 1 | 9.333238 | −3.661951 | −5.765387 |

| | | | |
|---|---|---|---|
| 1 | 8.405376 | −5.11771 | −5.31529 |
| 6 | 7.413797 | −3.297798 | −4.811196 |
| 6 | 7.154949 | −2.154021 | −5.549535 |
| 1 | 7.870697 | −1.862164 | −6.308103 |
| 6 | 6.080286 | −1.267305 | −5.291386 |
| 6 | 6.065652 | 0.012235 | −6.100053 |
| 1 | 5.616478 | 0.84523 | −5.558932 |
| 1 | 7.074805 | 0.287651 | −6.414645 |
| 1 | 5.466953 | −0.156591 | −7.002892 |
| 6 | 4.193173 | −0.468891 | −4.063227 |
| 6 | 4.493766 | 0.331082 | −2.935925 |
| 6 | 3.589491 | 1.341207 | −2.587008 |
| 1 | 3.795266 | 1.975577 | −1.731129 |
| 6 | 2.419693 | 1.545374 | −3.317403 |
| 6 | 2.13797 | 0.736772 | −4.413288 |
| 1 | 1.220708 | 0.895803 | −4.973834 |
| 6 | 3.012165 | −0.283716 | −4.809219 |
| 6 | 3.353451 | −5.211821 | −5.540841 |
| 1 | 2.343323 | −4.891677 | −5.242607 |
| 1 | 3.312054 | −5.382699 | −6.630134 |
| 6 | 3.640043 | −6.547353 | −4.86358 |
| 6 | 4.902951 | −6.851208 | −4.342316 |
| 1 | 5.697035 | −6.115363 | −4.41592 |
| 6 | 5.137629 | −8.076277 | −3.711917 |
| 1 | 6.123283 | −8.292259 | −3.305869 |
| 6 | 4.11404 | −9.020743 | −3.600478 |
| 1 | 4.294903 | −9.970378 | −3.103532 |
| 6 | 2.855065 | −8.731499 | −4.133345 |
| 1 | 2.044385 | −9.450186 | −4.046799 |
| 6 | 2.623982 | −7.505464 | −4.759318 |
| 1 | 1.633292 | −7.2802 | −5.146628 |
| 8 | 1.960339 | −4.686285 | −2.730695 |
| 6 | 1.471351 | −5.800677 | −1.950237 |
| 1 | 1.169544 | −5.465128 | −0.954533 |
| 1 | 2.291548 | −6.516247 | −1.842089 |
| 6 | 0.308887 | −6.419532 | −2.686455 |
| 6 | −0.43915 | −5.703213 | −3.625389 |
| 1 | −0.168754 | −4.678988 | −3.860185 |
| 6 | −1.511879 | −6.312925 | −4.280575 |
| 1 | −2.079684 | −5.751007 | −5.017266 |
| 6 | −1.851995 | −7.636214 | −3.994934 |
| 1 | −2.684728 | −8.109602 | −4.507723 |

| | | | |
|---|---|---|---|
| 6 | −1.112175 | −8.350872 | −3.048649 |
| 1 | −1.365676 | −9.383207 | −2.823413 |
| 6 | −0.035207 | −7.746029 | −2.401719 |
| 1 | 0.554214 | −8.3128 | −1.684935 |
| 6 | 2.694799 | −3.687404 | −2.2579 |
| 6 | 2.72288 | −3.411206 | −0.753362 |
| 6 | 4.317887 | −5.243353 | −0.072602 |
| 6 | 5.237328 | −5.077332 | −1.288417 |
| 1 | 1.679341 | −3.276316 | −0.440009 |
| 1 | 5.384125 | −6.101151 | −1.663781 |
| 8 | 3.012364 | −2.80404 | −3.100463 |
| 8 | 4.612365 | −4.294715 | −2.254281 |
| 1 | 1.729164 | 2.33334 | −3.028093 |
| 6 | 2.657278 | −1.148626 | −6.015537 |
| 1 | 3.464902 | −1.867745 | −6.174569 |
| 6 | 1.37568 | −1.968945 | −5.769629 |
| 1 | 1.168164 | −2.609135 | −6.635557 |
| 1 | 1.489671 | −2.602547 | −4.888125 |
| 1 | 0.507605 | −1.314523 | −5.620998 |
| 6 | 2.505725 | −0.301485 | −7.294358 |
| 1 | 1.644436 | 0.373737 | −7.224655 |
| 1 | 3.39057 | 0.314592 | −7.485447 |
| 1 | 2.347681 | −0.951264 | −8.163072 |
| 6 | 5.774832 | 0.116075 | −2.127866 |
| 1 | 5.983239 | −0.961134 | −2.125156 |
| 6 | 6.991056 | 0.811337 | −2.776164 |
| 1 | 6.798147 | 1.883815 | −2.899181 |
| 1 | 7.876026 | 0.6967 | −2.138913 |
| 1 | 7.235468 | 0.393695 | −3.754526 |
| 6 | 5.667522 | 0.576491 | −0.66359 |
| 1 | 5.661319 | 1.670142 | −0.586454 |
| 1 | 4.765433 | 0.201476 | −0.173134 |
| 1 | 6.536404 | 0.218596 | −0.100253 |
| 8 | 4.580828 | −6.025683 | 0.811322 |
| 8 | 3.139919 | −4.554687 | 0.029396 |
| 6 | 6.593218 | −4.520183 | −0.833109 |
| 1 | 7.014635 | −5.156969 | −0.050336 |
| 1 | 6.481303 | −3.503214 | −0.440949 |
| 1 | 7.266092 | −4.487397 | −1.691078 |
| 6 | 3.510125 | −2.162791 | −0.405712 |
| 1 | 4.54924 | −2.268101 | −0.718332 |
| 1 | 3.469447 | −1.997287 | 0.674163 |

| | | | |
|---|---|---|---|
| 1 | 3.085072 | −1.300218 | −0.924193 |

**Table S56.** Cartesian coordinate of **$L_{ax}$4c.**

| The number of atom coordinates (angstroms) | X | Y | Z |
|---|---|---|---|
| 13 | 4.838081 | −3.415035 | −3.853415 |
| 8 | 6.567112 | −3.962608 | −4.008908 |
| 8 | 3.938367 | −4.184026 | −5.096087 |
| 7 | 5.140111 | −1.619843 | −4.456669 |
| 6 | 8.550514 | −4.382659 | −5.228655 |
| 1 | 9.122692 | −4.509789 | −4.302302 |
| 1 | 9.200484 | −3.964344 | −6.000222 |
| 1 | 8.216077 | −5.379721 | −5.538671 |
| 6 | 7.350042 | −3.522514 | −4.955809 |
| 6 | 7.124001 | −2.344217 | −5.647104 |
| 1 | 7.834992 | −2.072504 | −6.416922 |
| 6 | 6.100092 | −1.40019 | −5.354848 |
| 6 | 6.136956 | −0.096903 | −6.117946 |
| 1 | 5.938627 | 0.757171 | −5.467465 |
| 1 | 7.099281 | 0.041801 | −6.613833 |
| 1 | 5.351874 | −0.107109 | −6.881974 |
| 6 | 4.243603 | −0.534391 | −4.112462 |
| 6 | 4.555961 | 0.245429 | −2.975384 |
| 6 | 3.709656 | 1.316128 | −2.669463 |
| 1 | 3.923712 | 1.943265 | −1.810889 |
| 6 | 2.579796 | 1.585793 | −3.440202 |
| 6 | 2.272409 | 0.777921 | −4.528266 |
| 1 | 1.374861 | 0.97698 | −5.106797 |
| 6 | 3.094154 | −0.297436 | −4.889224 |
| 6 | 3.029878 | −5.22572 | −5.303575 |
| 1 | 2.03937 | −4.950048 | −4.919653 |
| 1 | 2.913854 | −5.364144 | −6.390714 |
| 6 | 3.416569 | −6.564967 | −4.691168 |
| 6 | 4.699678 | −6.827073 | −4.199024 |
| 1 | 5.472086 | −6.069197 | −4.274626 |
| 6 | 5.003911 | −8.062523 | −3.618908 |
| 1 | 6.003651 | −8.241665 | −3.230979 |
| 6 | 4.032354 | −9.060901 | −3.538228 |
| 1 | 4.265968 | 10.018715 | −3.081004 |
| 6 | 2.755503 | −8.816874 | −4.052156 |
| 1 | 1.985598 | −9.581665 | −3.992558 |
| 6 | 2.453132 | −7.581164 | −4.623265 |

| | | | |
|---|---|---|---|
| 1 | 1.449298 | −7.394087 | −4.995815 |
| 8 | 1.705064 | −4.300749 | −2.492817 |
| 6 | 1.384757 | −5.567668 | −1.89485 |
| 1 | 1.104879 | −5.445659 | −0.845071 |
| 1 | 2.271699 | −6.208682 | −1.93476 |
| 6 | 0.24741 | −6.197956 | −2.665792 |
| 6 | −0.470638 | −5.505592 | −3.64416 |
| 1 | −0.217863 | −4.473733 | −3.862167 |
| 6 | −1.493944 | −6.145904 | −4.349918 |
| 1 | −2.039196 | −5.60029 | −5.115606 |
| 6 | −1.814055 | −7.476125 | −4.07689 |
| 1 | −2.607232 | −7.973034 | −4.628921 |
| 6 | −1.107638 | −8.166456 | −3.086505 |
| 1 | −1.349075 | −9.202853 | −2.865803 |
| 6 | −0.083386 | −7.530537 | −2.388006 |
| 1 | 0.481544 | −8.078204 | −1.637063 |
| 6 | 2.19691 | −3.228103 | −1.833018 |
| 6 | 2.475139 | −3.318413 | −0.328472 |
| 6 | 4.29981 | −5.049679 | −0.1611 |
| 6 | 5.159619 | −4.50316 | −1.304419 |
| 1 | 1.488664 | −3.354169 | 0.152313 |
| 1 | 5.479001 | −5.414202 | −1.830515 |
| 8 | 2.195434 | −2.158624 | −2.40599 |
| 8 | 4.433636 | −3.652692 | −2.15979 |
| 1 | 1.932956 | 2.419704 | −3.180372 |
| 6 | 2.695485 | −1.187535 | −6.06176 |
| 1 | 3.504847 | −1.896548 | −6.251391 |
| 6 | 1.450601 | −2.022825 | −5.702332 |
| 1 | 1.221634 | −2.727542 | −6.510751 |
| 1 | 1.613259 | −2.581501 | −4.778887 |
| 1 | 0.576919 | −1.375249 | −5.556205 |
| 6 | 2.454431 | −0.385869 | −7.354233 |
| 1 | 1.58836 | 0.279512 | −7.260542 |
| 1 | 3.317708 | 0.234492 | −7.621086 |
| 1 | 2.255762 | −1.068515 | −8.188573 |
| 6 | 5.769547 | −0.073214 | −2.100236 |
| 1 | 5.832816 | −1.165797 | −2.029581 |
| 6 | 7.095077 | 0.432345 | −2.707871 |
| 1 | 7.043357 | 1.511306 | −2.897221 |
| 1 | 7.921404 | 0.25172 | −2.010082 |
| 1 | 7.345962 | −0.068396 | −3.645033 |
| 6 | 5.648294 | 0.473203 | −0.667074 |

| | | | |
|---|---|---|---|
| 1 | 5.781918 | 1.561293 | −0.639924 |
| 1 | 4.679648 | 0.24198 | −0.216856 |
| 1 | 6.43172 | 0.035158 | −0.038477 |
| 8 | 4.697137 | −5.980404 | 0.504844 |
| 8 | 3.068193 | −4.554905 | 0.129455 |
| 6 | 6.411371 | −3.828922 | −0.720212 |
| 1 | 6.934492 | −4.518953 | −0.052055 |
| 1 | 6.14456 | −2.927701 | −0.159635 |
| 1 | 7.077937 | −3.548112 | −1.539608 |
| 6 | 3.224393 | −2.098251 | 0.179058 |
| 1 | 4.166283 | −1.991714 | −0.355956 |
| 1 | 3.409395 | −2.201534 | 1.252033 |
| 1 | 2.629992 | −1.200335 | −0.002939 |

**Table S57.** Cartesian coordinate of **L$_{ax}$5c.**

| The number of atom coordinates (angstroms) | X | Y | Z |
|---|---|---|---|
| 13 | 0.769012 | −0.608368 | 1.359333 |
| 8 | 0.843464 | 0.100162 | −0.303068 |
| 8 | −0.685771 | −1.46358 | 1.820611 |
| 7 | 0.368054 | 1.130329 | 2.243108 |
| 6 | 0.410175 | 1.461058 | −2.185523 |
| 1 | 1.407188 | 1.238073 | −2.57929 |
| 1 | 0.119227 | 2.474086 | −2.47414 |
| 1 | −0.287554 | 0.745398 | −2.634479 |
| 6 | 0.410354 | 1.267498 | −0.69477 |
| 6 | −0.012178 | 2.269297 | 0.154086 |
| 1 | −0.341266 | 3.201076 | −0.291176 |
| 6 | 0.010935 | 2.213796 | 1.579349 |
| 6 | −0.400482 | 3.489356 | 2.282362 |
| 1 | −0.2945 | 3.429622 | 3.364665 |
| 1 | 0.197939 | 4.327205 | 1.909343 |
| 1 | −1.447019 | 3.710675 | 2.04288 |
| 6 | 0.421289 | 1.143044 | 3.687005 |
| 6 | 1.647036 | 1.448031 | 4.320486 |
| 6 | 1.694454 | 1.413413 | 5.718675 |
| 1 | 2.624698 | 1.656771 | 6.22419 |
| 6 | 0.571816 | 1.081586 | 6.473098 |
| 6 | −0.626853 | 0.789008 | 5.830735 |
| 1 | −1.504862 | 0.54345 | 6.421908 |
| 6 | −0.730752 | 0.813096 | 4.433408 |
| 6 | −1.419812 | −2.402444 | 1.077293 |

| | | | |
|---|---|---|---|
| 1 | −0.859367 | −3.340514 | 0.941838 |
| 1 | −2.326091 | −2.652165 | 1.651968 |
| 6 | −1.829871 | −1.873541 | −0.28685 |
| 6 | −2.571436 | −0.687372 | −0.378878 |
| 1 | −2.851358 | −0.168164 | 0.534371 |
| 6 | −2.935105 | −0.167357 | −1.620017 |
| 1 | −3.509115 | 0.754284 | −1.674598 |
| 6 | −2.560943 | −0.829503 | −2.794759 |
| 1 | −2.846163 | −0.427221 | −3.76347 |
| 6 | −1.815433 | −2.007526 | −2.714518 |
| 1 | −1.512244 | −2.524215 | −3.621845 |
| 6 | −1.449962 | −2.520726 | −1.466183 |
| 1 | −0.851424 | −3.426527 | −1.406351 |
| 8 | 5.528583 | −3.507081 | −0.993994 |
| 6 | 4.578383 | −4.039097 | −1.971981 |
| 1 | 3.826541 | −4.66433 | −1.497861 |
| 1 | 5.194126 | −4.658699 | −2.63018 |
| 6 | 3.932609 | −2.907171 | −2.723817 |
| 6 | 2.569159 | −2.639376 | −2.567911 |
| 1 | 1.973235 | −3.235036 | −1.88542 |
| 6 | 1.971619 | −1.586459 | −3.263217 |
| 1 | 0.916459 | −1.382947 | −3.113156 |
| 6 | 2.736547 | −0.797308 | −4.122734 |
| 1 | 2.27314 | 0.021109 | −4.667652 |
| 6 | 4.102238 | −1.05722 | −4.281431 |
| 1 | 4.700291 | −0.442511 | −4.948724 |
| 6 | 4.696915 | −2.108217 | −3.585661 |
| 1 | 5.75866 | −2.310353 | −3.703889 |
| 6 | 5.474836 | −3.654713 | 0.33554 |
| 6 | 4.327935 | −4.416474 | 1.028654 |
| 6 | 2.449525 | −2.869682 | 0.985624 |
| 6 | 2.914476 | −2.106407 | 2.231745 |
| 1 | 4.431049 | −4.167085 | 2.082623 |
| 1 | 4.008336 | −1.989408 | 2.199946 |
| 8 | 6.368373 | −3.206227 | 1.022153 |
| 8 | 2.297762 | −0.869085 | 2.218717 |
| 1 | 0.63013 | 1.059105 | 7.558353 |
| 6 | −2.081375 | 0.532324 | 3.782963 |
| 1 | −1.936101 | 0.506504 | 2.701963 |
| 6 | −2.634947 | −0.845643 | 4.184755 |
| 1 | −3.566925 | −1.048523 | 3.642429 |
| 1 | −1.913682 | −1.625635 | 3.931795 |

| | | | |
|---|---|---|---|
| 1 | −2.858975 | −0.897721 | 5.257209 |
| 6 | −3.095194 | 1.645159 | 4.112462 |
| 1 | −3.302788 | 1.685752 | 5.188651 |
| 1 | −2.727332 | 2.631048 | 3.809136 |
| 1 | −4.045426 | 1.461975 | 3.595869 |
| 6 | 2.882818 | 1.872517 | 3.534539 |
| 1 | 2.709908 | 1.63816 | 2.482749 |
| 6 | 3.103981 | 3.393415 | 3.656365 |
| 1 | 3.289606 | 3.67909 | 4.699157 |
| 1 | 3.970754 | 3.705412 | 3.061134 |
| 1 | 2.232018 | 3.957068 | 3.308051 |
| 6 | 4.145878 | 1.106474 | 3.960632 |
| 1 | 4.449997 | 1.352116 | 4.985231 |
| 1 | 3.976664 | 0.029363 | 3.893963 |
| 1 | 4.981805 | 1.365368 | 3.299987 |
| 8 | 1.462261 | −2.462244 | 0.384811 |
| 8 | 3.023242 | −3.997225 | 0.545954 |
| 6 | 2.506216 | −2.898327 | 3.494896 |
| 1 | 2.990262 | −3.877207 | 3.580646 |
| 1 | 1.420111 | −3.034717 | 3.495857 |
| 1 | 2.77465 | −2.293901 | 4.365209 |
| 6 | 4.419348 | −5.929564 | 0.870052 |
| 1 | 4.327925 | −6.245927 | −0.171188 |
| 1 | 3.621419 | −6.401615 | 1.450342 |
| 1 | 5.384383 | −6.272232 | 1.255172 |
